# N-acetyl-l-leucine lowers **α**-synuclein levels and improves synaptic function in Parkinson’s disease models

**DOI:** 10.1172/JCI196137

**Published:** 2026-03-02

**Authors:** Pingping Song, Chuyu Chen, Rossella Franchini, Bryan Duong, Yi-Zhi Wang, Robert Coukos, Zhong Xie, Jeffrey N. Savas, Yueqin Zhou, Mariarita Bertoldi, D. James Surmeier, Loukia Parisiadou, Dimitri Krainc

**Affiliations:** 1 Ken and Ruth Davee Department of Neurology and; 2 Department of Pharmacology, Feinberg School of Medicine, Northwestern University, Chicago, Illinois, USA.; 3 Department of Neuroscience, Biomedicine and Movement Sciences, University of Verona, Verona, Italy.; 4 Department of Neuroscience, Feinberg School of Medicine, Northwestern University, Chicago, Illinois, USA.

**Keywords:** Cell biology, Neuroscience, Neurodegeneration, Parkinson disease

## Abstract

N-acetyl-l-leucine (NALL), a derivative of the branched-chain amino acid leucine, has shown therapeutic potential for neurodegenerative diseases, including in prodromal stages of Parkinson’s disease (PD). However, the mechanism of its protective effects has been largely unknown. Using human induced pluripotent stem cell–derived dopaminergic neurons from patients carrying *GBA1*, *LRRK2*, or *VPS35* mutations, as well as from sporadic PD cases, we found that NALL treatment markedly reduced Ser129 phosphorylated α-synuclein (pS129-syn). Discovery-based proteomic analysis revealed that NALL treatment upregulated lysosomal, mitochondrial, and synaptic proteins without inducing cytotoxicity. The reduction of pS129-syn was dependent on serine protease HTRA1, which was robustly induced by NALL. Moreover, NALL increased the expression of wild-type parkin in mutant dopaminergic neurons, leading to increased glycosylated dopamine transporter, elevated synaptic membrane-associated synaptojanin-1, and accelerated synaptic vesicle endocytosis, suggesting improved synaptic function. Furthermore, in *LRRK2*^R1441C^ knockin mice, NALL administration decreased pS129-syn, elevated parkin levels, and ameliorated dopamine-dependent motor learning deficits. These findings highlight the therapeutic potential of NALL for PD by its protective effects on α-synuclein pathology and synaptic function in vulnerable dopaminergic neurons.

## Abstract 2

N-acetyl-l-leucine is a promising therapeutic candidate for Parkinson’s disease, by targeting alpha-synuclein accumulation and synaptic dysfunction in PD.

## Introduction

Parkinson’s disease (PD) affects 1%–2% of the population and is characterized by resting tremor, rigidity, and bradykinesia. These PD motor symptoms are primarily due to the progressive loss of dopaminergic neurons in the substantia nigra pars compacta (1, 2). The pathogenesis of PD involves complex interactions between genetic and environmental factors. Among the genetic factors, mutations in the genes encoding leucine-rich repeat kinase 2 (LRRK2), glucocerebrosidase (GBA), and parkin all have been identified as significant contributors that affect mitochondrial, lysosomal, and synaptic pathways (3).

A key pathological feature of PD is the age-dependent accumulation of α-synuclein (α-syn) into intracellular aggregates, forming spherical inclusions within the cytoplasm and neuronal processes known as Lewy bodies and Lewy neurites (4). α-Syn is thought to play a central role in the pathobiology of both familial and sporadic PD. Several posttranslational modifications to α-syn are known to occur in PD. Among them is phosphorylation of α-syn at Ser129 (pS129-syn), a modification that plays a critical role in PD pathogenesis (5, 6). pS129-syn has been reported to enhance α-syn toxicity both in vivo and in vitro, possibly by increasing the formation of α-syn aggregates (6, 7). Several current studies seek to find drugs that lower the accumulation and aggregation of α-syn in the brains of patients with PD (8–10).

N-acetyl-l-leucine (NALL) is the l-enantiomer of N-acetyl-dl-leucine (ADLL), a derivative of the branched-chain amino acid leucine. ADLL (Tanganil) has been used clinically for many years to treat acute vertigo and related vestibular symptoms, with an excellent safety profile (11, 12). NALL has also received FDA approval for the treatment of one of the lysosomal storage disorders, Niemann-Pick disease type C1 (NPC1) (13), further supporting its safety in humans. Clinical and preclinical studies have suggested that acetyl-leucine derivatives may provide symptomatic or functional benefits in another lysosomal storage disorder, GM2 gangliosidoses (14–16), as well as in some cases of cerebellar ataxia (17–19). Limited exploratory evidence also suggests possible effects in conditions such as restless legs syndrome (20), and more recently REM sleep behavior disorder that is considered a prodrome of PD (21), though these findings require cautious interpretation and further validation. While NALL is hypothesized to be the pharmacologically active enantiomer (22, 23), robust clinical evidence directly supporting its specific therapeutic efficacy remains limited, and the underlying mechanisms of NALL’s potential neuroprotective effects are still largely unknown.

In this study, we found that NALL, but not its d-enantiomer, NADL, significantly reduced pS129-syn in human induced pluripotent stem cell– derived (iPSC-derived) dopaminergic neurons from patients carrying *GBA1*, *LRRK2*, or *VPS35* mutations, as well as from sporadic PD cases. In addition, NALL treatment enhanced the expression of parkin and the glycosylated, functionally mature form of the dopamine transporter (DAT), and enhanced synaptic vesicle endocytosis, indicating improved synaptic function. Importantly, in *LRRK2*^R1441C^ knockin mice, NALL administration ameliorated behavioral deficits, further providing in vivo evidence for its potential therapeutic benefit in PD.

## Results

### NALL leads to decreased pS129-syn in human dopaminergic neurons with GBA1 mutations.

**Figure 1:**
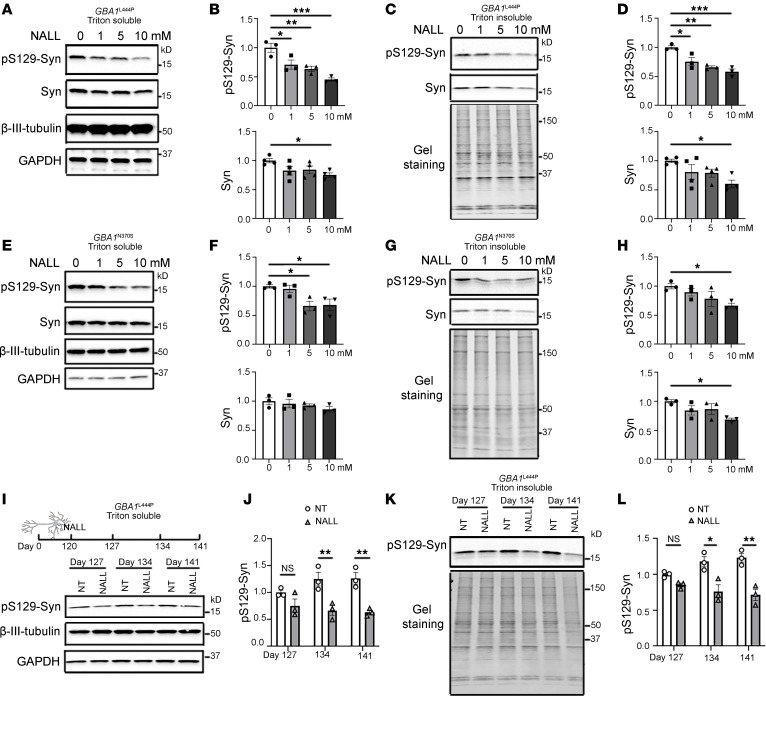
NALL leads to decreased pS129-syn in human dopaminergic neurons with *GBA1* mutations. (**A**) Representative Western blot showing pS129-syn and total α-syn (Syn) levels following 30 days of treatment with increasing concentrations of NALL in the Triton-soluble fraction of *GBA1* L444P mutant dopaminergic neurons. β-III-tubulin and GAPDH served as loading controls. (**B**) Quantification of pS129-syn (top) and total Syn (bottom) signals in **A**, normalized to β-III-tubulin and expressed relative to the 0 mM (DMSO) group (*n* = 3–4 independent experiments; 1-way ANOVA). (**C**) Western blot of pS129-syn and total Syn in the Triton-insoluble fraction of *GBA1* L444P mutant neurons following NALL treatment; total protein staining served as a loading control. (**D**) Quantification of pS129-syn (top) and total Syn (bottom) signals in **C**, normalized to total protein and expressed relative to the 0 mM (DMSO) group (*n* = 3–4 independent experiments; 1-way ANOVA). (**E**–**H**) Similar analyses performed in *GBA1* N370S mutant dopaminergic neurons. (**E** and **G**) Representative blots of soluble (**E**) and insoluble (**G**) fractions with corresponding quantifications (**F** and **H**), normalized and expressed relative to the 0 mM (DMSO) group (*n* = 3 independent experiments; 1-way ANOVA). (**I**) (Top) Schematic of the experimental design showing NALL treatment (10 mM) of *GBA1* L444P mutant dopaminergic neurons at day 120 for 7, 14, or 21 days. (Bottom) Western blot of pS129-syn in the Triton-soluble fraction with or without NALL treatment. β-III-tubulin and GAPDH were used as loading controls. (**J**) Quantification of pS129-syn levels in **I**, normalized to β-III-tubulin and expressed relative to the nontreated (NT; DMSO) group (*n* = 3 independent experiments; 2-way ANOVA). (**K**) Western blot of pS129-syn in the Triton-insoluble fraction of *GBA1* L444P neurons with or without NALL treatment; total protein staining was used as a loading control. (**L**) Quantification of pS129-syn in **K**, normalized to total protein and expressed relative to the NT (DMSO) group (*n* = 3 independent experiments; 2-way ANOVA). All data are presented as mean ± SEM. *P < 0.05, **P < 0.01, and ***P < 0.005.

To investigate the molecular mechanisms underlying the potential neuroprotective effects of NALL in PD, we treated iPSC-derived dopaminergic neurons carrying a *GBA1* mutation L444P (*GBA1*^L444P^), which impairs lysosomal function (24), with increasing concentrations of NALL on day 120 for 4 weeks. We then examined NALL’s effects on the protein levels and solubility of α-syn and its phosphorylated form, pS129-syn, a modification closely associated with PD pathology (6, 25, 26). A decrease of pS129-syn was observed in a dose-dependent manner in both Triton-soluble and -insoluble fractions (Figure 1, A–D), and total α-syn levels were decreased with the highest dose (10 mM) of NALL. The effect of NALL on pS129 and total α-syn was replicated in another *GBA1* mutant N370S (*GBA1*^N370S^) line, where we observed a decrease in pS129 and total syn upon NALL treatment (Figure 1, E–H). This excludes line-specific effects of NALL and further suggests a beneficial effect of NALL in GBA-linked PD. To assess time-dependent effects, we analyzed Triton-soluble and -insoluble fractions of α-syn in *GBA1*^L444P^ mutant neurons treated with NALL for 7, 14, and 21 days. In untreated neurons, pS129-syn levels showed an increased trend over time. In contrast, NALL treatment led to a noticeable reduction in pS129-syn after 7 days (DIV 127) and a significant decrease as early as 14 days (DIV 134) (Figure 1, I–L), indicating that NALL attenuates the progressive accumulation of α-syn in *GBA1*-mutant neurons. As a comparison, treatment with the d-enantiomer, NADL, for 14 days did not reduce either pS129-syn or total α-syn levels in Triton-soluble (Supplemental Figure 1, A and B; supplemental material available online with this article; https://doi.org/10.1172/JCI196137DS1) or -insoluble fractions (Supplemental Figure 1, C and D), further supporting NALL as the therapeutically active enantiomer.

### Decreased pS129-syn upon NALL treatment is mediated by HTRA1.

**Figure 2:**
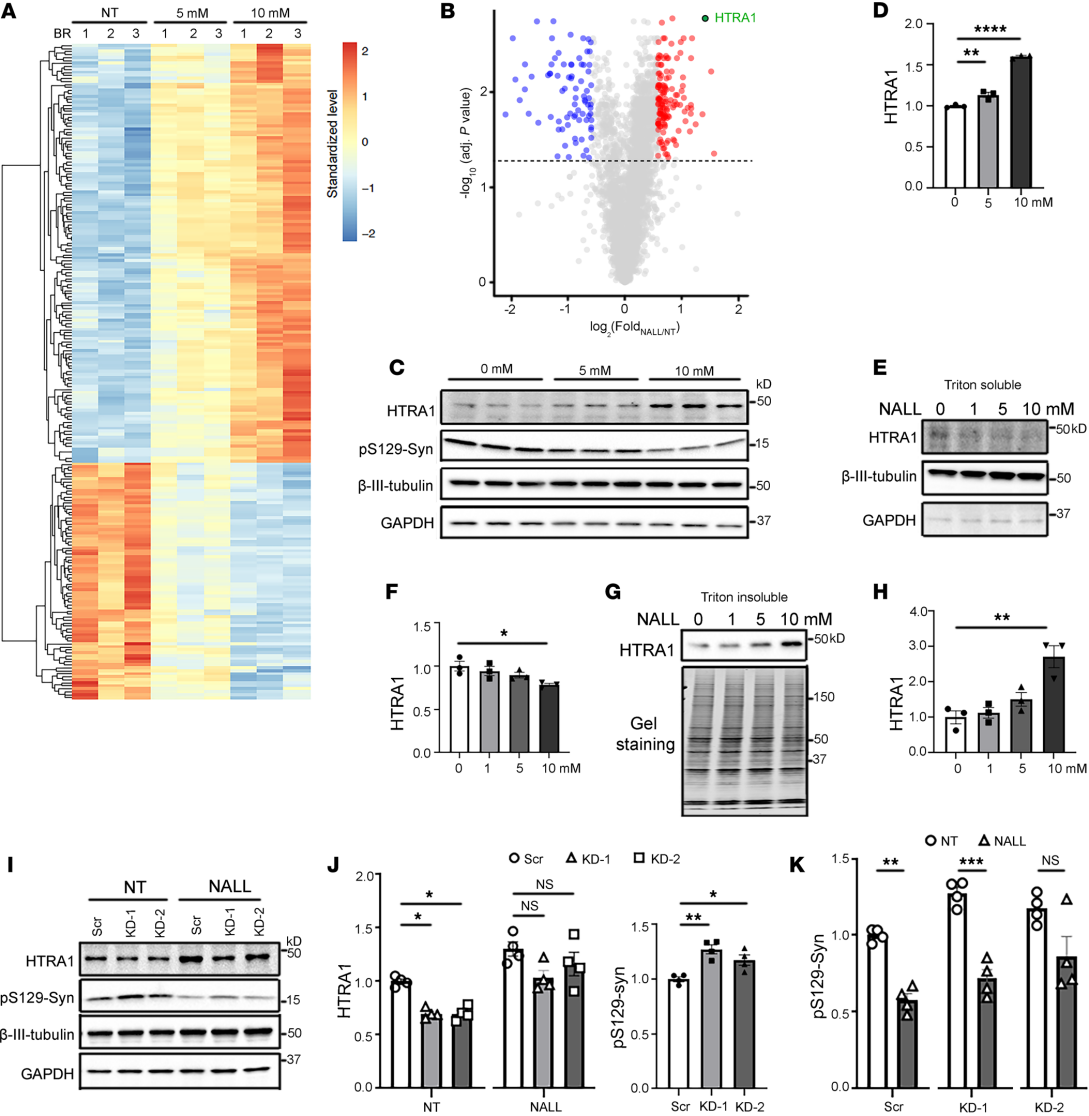
The effect of NALL on pS129-syn was mediated through increasing HTRA1. (**A**) Heatmap showing proteins significantly altered (adjusted *P* < 0.05) by 30 days of NALL treatment in *GBA1* L444P mutant neurons (*n* = 3 biological replicates; 3,801 total proteins). (**B**) Volcano plot of protein changes induced by 10 mM NALL versus nontreated (NT) controls. HTRA1 (green) and other significantly altered proteins. Dashed line, adjusted *P* = 0.05 threshold (1-way ANOVA with Benjamini-Hochberg correction). (**C**–**H**) Representative Western blots and corresponding quantification of HTRA1 and pS129-syn in total lysates (**C** and **D**), Triton-soluble fractions (**E** and **F**), and Triton-insoluble fractions (**G** and **H**) of *GBA1* L444P neurons treated with increasing NALL concentrations for 30 days. β-III-tubulin, GAPDH, or total protein staining served as loading controls (*n* = 3 independent experiments). (**I**–**K**) Western blot analysis (**I**) and quantification of HTRA1 (**J**) and pS129-syn (**K**) in *GBA1* L444P neurons following HTRA1 knockdown (KD-1 and KD-2) with or without 10 mM NALL. Data were normalized to GAPDH or β-III-tubulin and expressed relative to NT-scramble controls (*n* = 4 independent experiments). Statistical significance was determined by 1-way ANOVA with Benjamini-Hochberg correction (**B**), 1-way ANOVA (**D**, **F**, **H**, and **J** right), or 2-way ANOVA (**J** left and **K**). Data represent mean ± SEM. *P < 0.05, **P < 0.01, ***P < 0.005, and ****P < 0.001.

To further elucidate the molecular mechanisms underlying the effects of NALL, we performed tandem mass tag (TMT) mass spectrometry (MS)–based quantitative proteomic analysis in *GBA1*^L444P^ mutant dopaminergic neurons. This analysis revealed 896 proteins with significantly elevated levels and 283 proteins with decreased levels (Supplemental Table 1). The proteins that were increased by at least 1.5-fold (adjusted *P* < 0.05) are involved in mitochondrial, lysosomal, and synaptic pathways, whereas proteins that were decreased are involved in cytoskeletal function (Figure 2A, Supplemental Figure 2, and Supplemental Tables 1 and 2). Importantly, NALL-induced pathway activation did not trigger cytotoxic stress. Proteomic profiling showed that NALL upregulated proteins involved in the mitochondrial electron transport chain (Supplemental Figure 3A) and lysosomal enzymes (Supplemental Figure 3B) while downregulating the pro-apoptotic protein BAX (Supplemental Figure 3C). Moreover, expression levels of GPX4 and ACSL4 remained unchanged (Supplemental Figure 3D), suggesting that ferroptosis pathways were not affected. Consistently, NALL treatment did not activate caspase-3 (Supplemental Figure 3E), further supporting that its effects are protective rather than cytotoxic. Furthermore, we found that NALL treatment significantly enhanced both ATP-linked respiration and maximum respiratory capacity compared with untreated controls (Supplemental Figure 4, A–F) in *GBA1*-mutant neurons. Interestingly, mitochondrial DNA D-LOOP and ND1 levels remained unchanged (Supplemental Figure 4G), suggesting that the improved mitochondrial function was not due to increased mitochondrial copy number but rather to enhanced mitochondrial efficiency.

Serine protease HTRA1 emerged as one of the proteins exhibiting the most pronounced and consistently elevated fold-change (Figure 2B). It is an ATP-independent PDZ serine protease that has been reported to disaggregate α-syn amyloid fibrils in vitro and prevent the accumulation of Lewy body–like inclusions composed of hyperphosphorylated α-syn in mice (27). Based on this, we hypothesized that elevated HTRA1 might contribute to the NALL-mediated reduction of pS129-syn. Consistent with the proteomic data, biochemical analysis revealed that NALL treatment elevated HTRA1 protein levels in *GBA1*^L444P^ mutant neurons compared with untreated controls (Figure 2, C and D). This increase in HTRA1 protein was likely mediated at the transcriptional level, as evidenced by the upregulation of *HTRA1* mRNA expression (Supplemental Figure 5A). Furthermore, in agreement with prior findings that HTRA1 localizes to the insoluble fraction in the absence of α-syn and to the soluble fraction in its presence (27), we observed decreased HTRA1 in the Triton-soluble fraction and increased HTRA1 in the insoluble fraction after NALL treatment (Figure 2, E–H). To test whether HTRA1 mediates the NALL-induced reduction of pS129-syn, we used 2 independent shRNA lentiviral constructs (KD-1 and KD-2) to knock down HTRA1 under both nontreated (NT) and NALL-treated conditions (Figure 2, I and J). In control cells (scrambled shRNA), NALL treatment increased HTRA1 and decreased pS129-syn levels, consistent with previous findings. HTRA1 expression was reduced by approximately 30% following knockdown in untreated conditions (Figure 2, I and J). Also the pS129-syn was increased upon HTRA1 knockdown (Figure 2, I and J). However, NALL treatment markedly diminished knockdown efficiency, likely due to strong transcriptional induction of HTRA1 by NALL or limited viral transduction efficiency, where HTRA1 level was increased in nontransduced cells. Under such conditions, HTRA1 knockdown (KD-1) did not abolish the NALL-induced reduction of pS129-syn (Figure 2, I and K). To further validate HTRA1’s role, we examined α-syn–expressing SH-SY5Y cells, which lack endogenous HTRA1 expression (28). NALL treatment failed to reduce pS129-syn in these cells (Supplemental Figure 5, B and C). However, overexpression of HTRA1 in these cells lowered pS129-syn levels (Supplemental Figure 5, D and E), supporting that NALL’s effect on pS129-syn is mediated through HTRA1.

### NALL increases glycosylated dopamine transporter and synaptic function through parkin.

**Figure 3:**
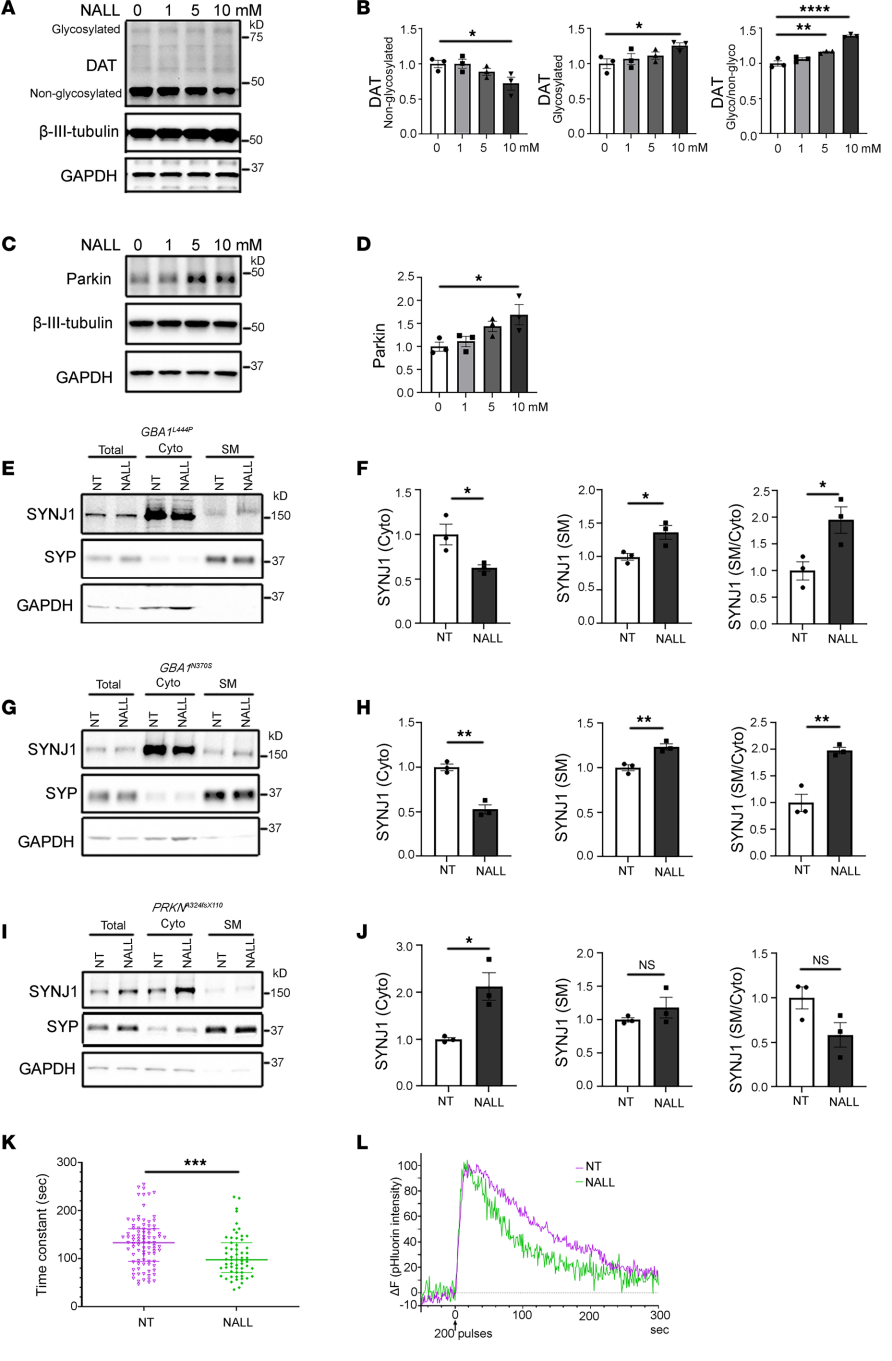
NALL increases functional dopamine transporter and synaptic function through parkin. (**A** and **B**) Representative Western blot (**A**) and quantification (**B**) of glycosylated and nonglycosylated dopamine transporter (DAT) in *GBA1* L444P mutant dopaminergic neurons treated with increasing NALL concentrations. (**C** and **D**) Western blot (**C**) and quantification (**D**) of parkin expression in NALL-treated *GBA1* L444P neurons. (**E**–**J**) Western blots and corresponding quantification of SYNJ1 redistribution from cytosolic (Cyto) to synaptic membrane (SM) fractions following NALL treatment in *GBA1* L444P (**E** and **F**), *GBA1* N370S (**G** and **H**), and parkin mutant (**I** and **J**) dopaminergic neurons. Synaptophysin (SYP) and GAPDH served as synaptic and cytosolic markers, respectively. (**K**) Scatter plot showing time constants of pHluorin fluorescence recovery following exocytosis in *GBA1* L444P neurons. NALL-treated synapses (105.3 ± 5.8 s; *n* = 59 ROIs) recovered significantly faster than NT (133.2 ± 5.3 s; *n* = 89 ROIs) (Mann-Whitney test). Lines represent means ± SD. (**L**) Representative traces of pHluorin fluorescence intensity from 50 s before to 300 s after exocytosis in *GBA1* L444P mutant neurons. For Western blots, β-III-tubulin and GAPDH served as loading controls (*n* = 3 independent experiments). Data are expressed as mean fold-change relative to 0 mM (DMSO) or nontreated (NT) groups. Statistical significance was determined by 1-way ANOVA (**B** and **D**), Student’s *t* test (**F**, **H**, and **J**), or Mann-Whitney test (**K**). Data represent mean ± SEM, except in **K** (mean ± SD). *P < 0.05, **P < 0.01, ***P < 0.005, and ****P < 0.001.

Recent findings have shown that ADLL treatment improves DAT scan signals in patients with REM sleep behavior disorder (21). To investigate the underlying mechanisms of these clinical observations, we examined DAT in iPSC-derived GBA1-mutant dopaminergic neurons. We found that nonglycosylated DAT was decreased (Figure 3, A and B), while glycosylated DAT — representing the mature, cell surface–expressed form (29, 30) — was increased upon NALL treatment (Figure 3, A and B). Consequently, the ratio of glycosylated to nonglycosylated DAT was elevated (Figure 3B), suggesting enhanced functional DAT expression. Previous studies have reported that parkin promotes the surface expression of DAT by ubiquitinating and degrading misfolded, nonglycosylated DAT, thereby facilitating proper oligomerization and membrane localization of native DAT (30). To test whether the effects of NALL on DAT might be mediated by parkin, we examined parkin levels in GBA1-mutant dopaminergic neurons. Interestingly, we found an increase in parkin levels upon treatment with NALL (Figure 3, C and D), suggesting that increased parkin might contribute to the observed increase in functional DAT. The increased parkin protein was likely mediated at the transcriptional level, as evidenced by the upregulation of parkin gene expression (Supplemental Figure 6A). As a further test of this hypothesis, dopaminergic neurons derived from patients with *PRKN*^A324fsX110^ were treated with NALL. We found that in the absence of parkin (31), NALL treatment did not alter the levels of either glycosylated or nonglycosylated DAT (Supplemental Figure 6, B and C), indicating that NALL-induced enhancement of functional DAT is at least partially dependent on parkin.

Our prior work demonstrated that parkin facilitates synaptic vesicle recycling by ubiquitination-mediated membrane recruitment of synaptojanin-1 (SYNJ1), which promotes clathrin uncoating during endocytosis (31). Consistent with this, NALL treatment decreased cytosolic SYNJ1 levels while increasing its synaptic membrane association in both *GBA1*^L444P^ (Figure 3, E and F) and *GBA1*^N370S^ (Figure 3, G and H) mutant neurons, suggesting enhanced clathrin uncoating and synaptic vesicle endocytosis. To test whether the NALL-induced increase in membrane-associated SYNJ1 depends on parkin, we treated parkin-mutant dopaminergic neurons with NALL. Strikingly, NALL failed to increase membrane-associated SYNJ1 in parkin-mutant neurons (Figure 3, I and J), indicating that the NALL-induced redistribution of SYNJ1 is parkin dependent. Functionally, we assessed synaptic vesicle cycling using a synaptophysin-pHluorin optical reporter (32, 33) and found that synaptic vesicle retrieval following action potentials was significantly faster in NALL-treated neurons compared with untreated neurons (Figure 3, K and L), indicating enhanced synaptic endocytosis efficiency. Together, these findings demonstrate that NALL increases functional DAT through parkin and promotes parkin-dependent recruitment of SYNJ1 to synaptic membrane, thereby facilitating clathrin uncoating, accelerating synaptic vesicle recycling, and improving synaptic function.

### NALL decreases pS129-syn and increases parkin across familial and sporadic forms of PD.

**Figure 4:**
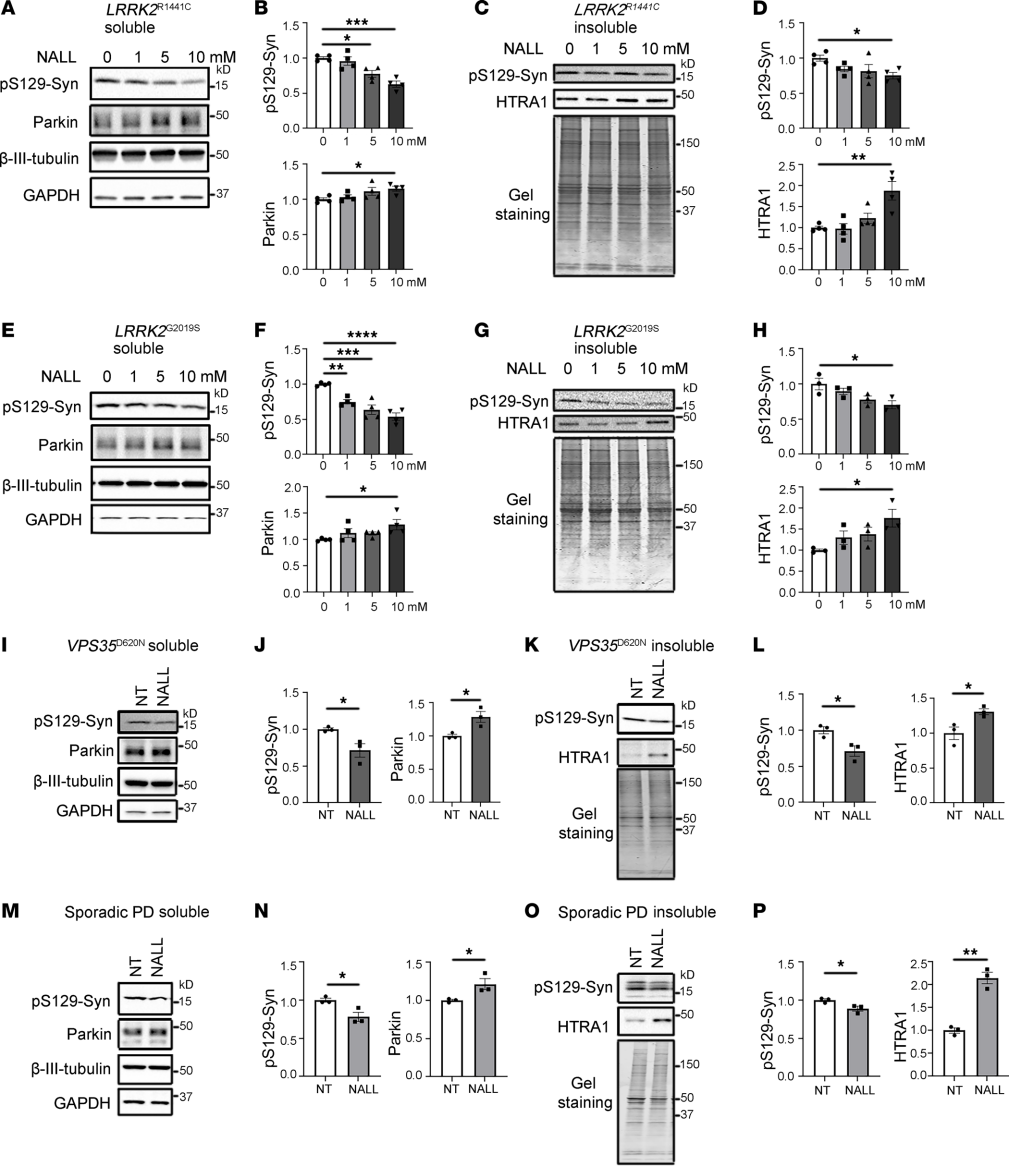
NALL exhibits broad neuroprotective effects across familial and sporadic forms of PD by reducing pS129-syn and enhancing parkin. (**A**–**D**) Representative Western blots and quantification of pS129-syn, parkin, and HTRA1 in *LRRK2* R1441C mutant dopaminergic neurons treated with increasing NALL concentrations for 14 days. Analysis was performed in Triton-soluble (**A** and **B**) and Triton-insoluble (**C** and **D**) fractions. (**E**–**H**) Western blot and quantification of pS129-syn, parkin, and HTRA1 in soluble and insoluble fractions of *LRRK2* G2019S mutant neurons treated with NALL. (**I**–**L**) Western blot and quantification of pS129-syn, parkin, and HTRA1 in *VPS35* D620N mutant dopaminergic neurons treated with or without NALL. (**M**–**P**) Western blot and quantification of pS129-syn, parkin, and HTRA1 in dopaminergic neurons derived from patients with sporadic PD treated with or without NALL. Data are expressed relative to the 0 mM (DMSO) or nontreated group (*n* = 3–4 independent experiments). Statistical significance was determined by 1-way ANOVA (**B**, **D**, **F**, and **H**) or Student’s *t* test (**J**, **L**, **N**, and **P**). All data represent mean ± SEM. *P < 0.05, **P < 0.01, ***P < 0.005, ****P < 0.001.

Our findings thus far suggest that NALL reduces pathogenic pS129-syn levels through HTRA1 and enhances synaptic function in *GBA1*-mutant dopaminergic neurons. To determine whether NALL confers similar benefits across other forms of PD, we examined its effects in patient-derived dopaminergic neurons carrying the *LRRK2* R1441C mutation, *LRRK2*^R1441C^, and in *LRRK2* G2019S–knockin, *LRRK2*^G2019S^, neurons on the healthy KOLF background, both of which display elevated LRRK2 kinase activity. Consistent with findings in *GBA1*-mutant neurons, 2 weeks of NALL treatment led to decreased pS129-syn and increased parkin and elevated Triton-insoluble HTRA1 levels in both *LRRK2*^R1441C^ (Figure 4, A–D) and *LRRK2*^G2019S^ (Figure 4, E–H) mutant neurons. In addition, NALL treatment enhanced synaptic vesicle endocytosis in *LRRK2*-mutant neurons (Supplemental Figure 7). Similarly, NALL reduced pS129-syn and elevated parkin and Triton-insoluble HTRA1 expression in dopaminergic neurons derived from patients harboring the *VPS35* D620N mutation, *VPS35*^D620N^ (Figure 4, I–L), as well as from sporadic PD cases (Figure 4, M–P). Collectively, these results indicate that the neuroprotective actions of NALL are not restricted to GBA1-associated PD but extend to multiple genetic and idiopathic forms of the disease, highlighting its broad therapeutic potential.

### NALL restores molecular and motor learning deficits in LRRK2ᴿ¹^44^¹^C^ mice.

**Figure 5:**
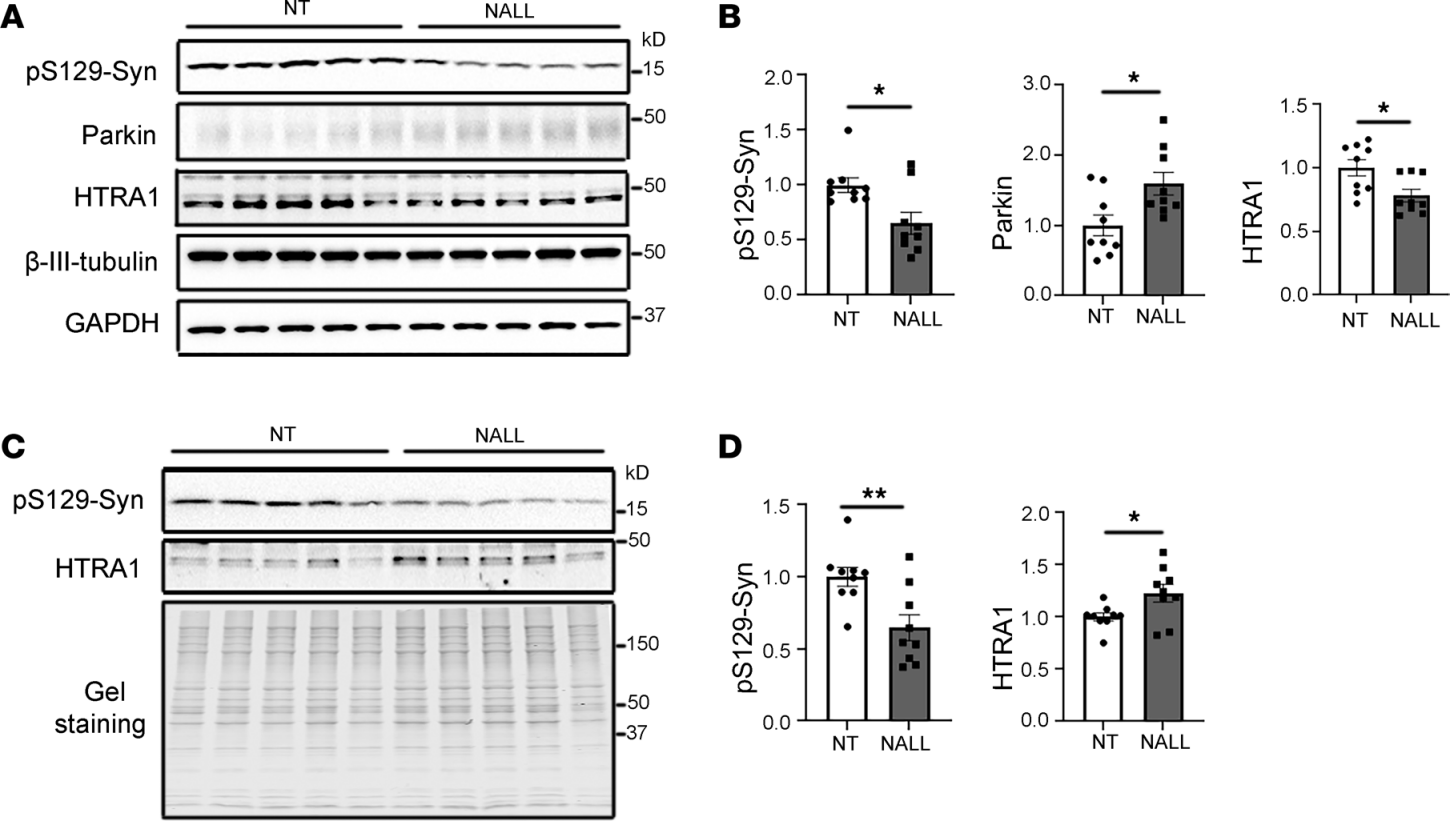
NALL leads to decreased pS129-syn but increased parkin in *LRRK2*^R1441C^ mice. (**A**) Western blots showing pS129-syn, parkin, and HTRA1 in the Triton-soluble fraction of the substantia nigra from *LRRK2*^R1441C^ mice treated with NALL (NALL) or vehicle (NT). β-III-tubulin and GAPDH were used as loading controls. (**B**) Quantification of fold-changes in pS129-syn (left), parkin (middle), and HTRA1 (right) following NALL treatment shown in **A**. Data represent average pS129-syn levels (normalized to β-III-tubulin), parkin (normalized to GAPDH), and HTRA1 (normalized to GAPDH), expressed relative to the NT (vehicle-only) group (*n* = 9 mice; *t* test). (**C**) Western blots showing pS129-syn and HTRA1 in the Triton-insoluble fraction of *LRRK2*^R1441C^ mouse substantia nigra with or without NALL treatment. Total protein staining was used as a loading control. (**D**) Quantification of fold-changes in pS129-syn and HTRA1 from **C**. Data represent average pS129-syn and HTRA1 (normalized to total protein) levels relative to the NT (vehicle-only) group (*n* = 9 mice; *t* test). Data are shown as mean ± SEM; *P < 0.05, **P < 0.01.

**Figure 6:**
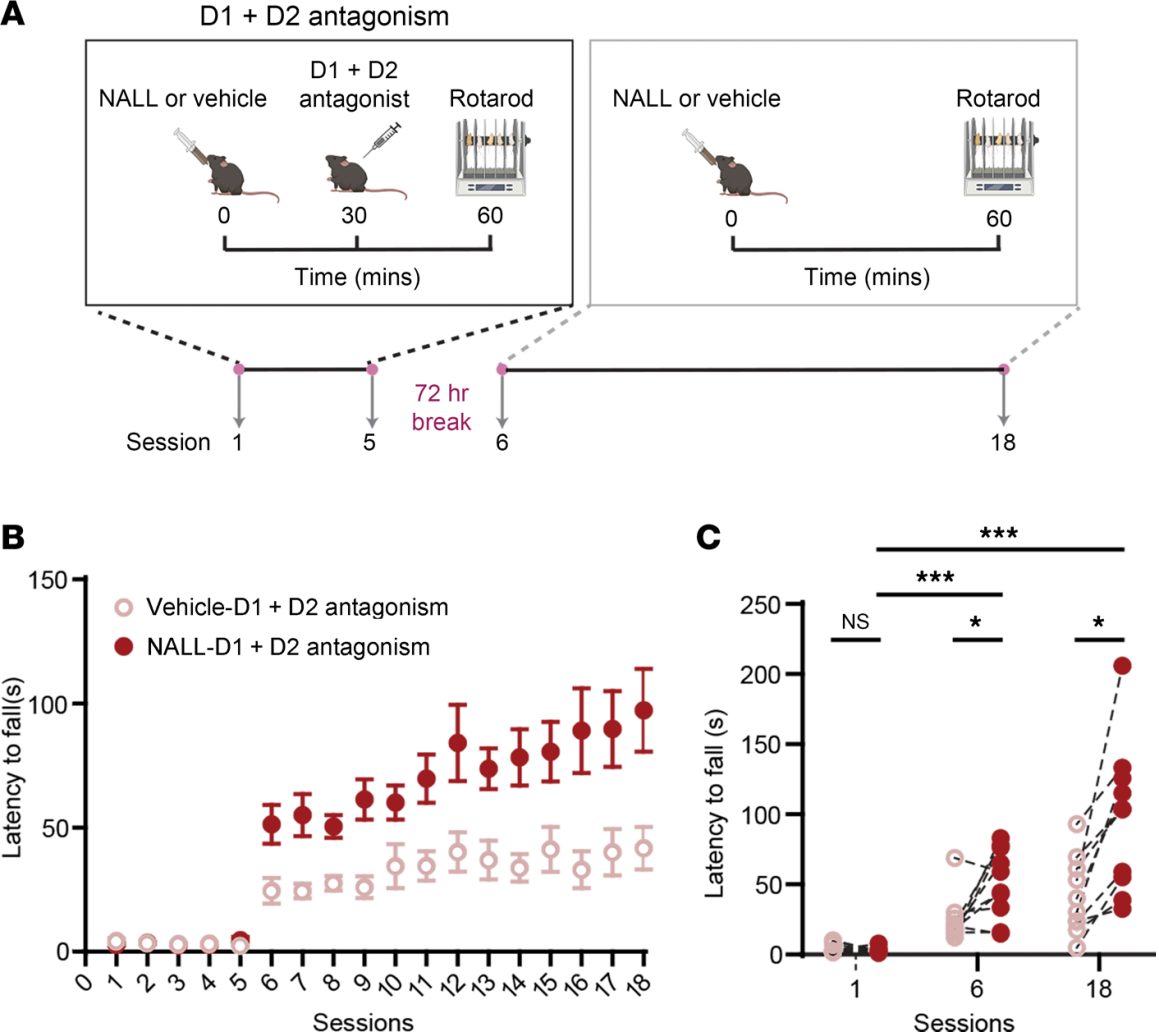
NALL improves dopamine-dependent motor learning impairments in *LRRK2*^R1441C^ mice. (**A**) Schematic of the rotarod training paradigm. *LRRK2*^R1441C^ mice were evaluated over a total of 18 daily sessions, with each session consisting of 5 trials. The mice were administered either NALL or a vehicle control intraperitoneally 30 minutes before receiving either saline or a cocktail of dopamine D1 receptor antagonists (SCH23390) and D2 receptor antagonists (eticlopride), both at a dosage of 1 mg/kg. Thirty minutes after injection, mice were trained on an accelerating rotarod for 5 consecutive days. After a 72-hour break, mice were returned to the rotarod for an additional 13 days, with either NALL or vehicle administered 60 minutes before each session. (**B**) *LRRK2*^R1441C^ mice that received NALL exhibited improved rotarod performance — indicated by the latency to fall from the rotarod — compared with vehicle-treated controls (treatment *P* = 0.0013, session *P* < 0.0001, treatment × session factor *P* < 0.0001, subject *P* < 0.0001). (**C**) Summary of average latency to fall in session 1, session 6, and session 18 from **B**. Statistical significance was determined by 2-way repeated measures ANOVA followed by Tukey’s multiple comparisons test (*P < 0.05, ***P < 0.005). *n* = 10 mice per treatment group.

Building on these findings from patient-derived neurons, we next sought to determine whether NALL exerts similar protective effects in vivo. To this end, we treated *LRRK2*^R1441C^ knockin mice — a well-established model exhibiting dopamine-dependent motor learning deficits (34) —with NALL and examined its biochemical and behavioral impact. We observed a significant reduction in pS129-syn levels in both Triton-soluble (Figure 5, A and B) and Triton-insoluble (Figure 5, C and D) fractions following NALL treatment. Consistent with our findings in patient-derived dopaminergic neurons (Figure 2, E–H), HTRA1 levels decreased in the Triton-soluble fraction but increased in the Triton-insoluble fraction (Figure 5, A–D), further supporting the role of HTRA1 in mediating NALL’s effect on pS129-syn. In parallel, NALL treatment significantly elevated parkin protein levels in *LRRK2*^R1441C^ mice (Figure 5, A and B), consistent with the upregulation observed in patient-derived neurons (Figure 3, C and D, and Figure 4). Given that NALL similarly modulated α-syn and parkin levels in both *LRRK2*-mutant neurons and mice, we next assessed whether it could ameliorate the observed dopamine-mediated motor learning deficits in *LRRK2*^R1441C^ mice (34). Previous studies have shown that disrupting dopamine signaling during motor skill acquisition in specific contexts can impair future performance, even after dopamine signaling (35–37). Relatedly, in our earlier work, we used a 2-stage accelerating rotarod test that included D1-like and D2-like receptor blockers during the first phase to temporarily inhibit dopamine signaling and assess dopamine-dependent motor learning in *LRRK2*^R1441C^ mice (Figure 6A) (34), We showed that while wild-type (*LRRK2*^WT^) mice improved their performance during the antagonist-free phase, *LRRK2*^R1441C^ mutants failed to show similar improvement, confirming disrupted dopamine-mediated motor learning (34). Remarkably, here, we showed that when NALL was administered throughout the task (Figure 6), *LRRK2*^R1441C^ mice exhibited a significant enhancement in motor performance compared with vehicle-treated controls during the second, antagonist-free phase (treatment × session interaction *P* = 0.0013, 2-way repeated measures ANOVA). Specifically, at sessions 6 and 18, NALL-treated mice showed greater latency to fall than vehicle-treated mice (*P* = 0.0123 and *P* = 0.0108, respectively; Tukey’s multiple comparisons). Interestingly, NALL treatment did not alter pS129-syn or HTRA1 levels and had no measurable effect on their motor learning performance in wild-type cohorts (Supplemental Figure 8, E and F). Together, these results demonstrate that NALL improves dopamine-dependent motor learning in *LRRK2*^R1441C^ mice and that its effects on pS129-syn and HTRA1 are context dependent, likely requiring the presence of pathogenic mutations or underlying cellular stress to manifest.

## Discussion

We found that treatment of dopaminergic neurons derived from patients carrying *GBA1*, *LRRK2*, or *VPS35* mutations, as well as a sporadic PD case with NALL, led to a reduction in pS129-syn. To elucidate the underlying mechanism, we performed discovery-based proteomic analysis, which revealed a significant upregulation of the serine protease HTRA1 in human dopaminergic neurons following NALL exposure. HTRA1 has previously been reported to protect against α-syn aggregation and hyperphosphorylation (27). Consistent with this, our data demonstrate that NALL-mediated reduction of pS129-syn is dependent on HTRA1 expression, indicating that activation of this protease may represent a novel therapeutic strategy for mitigating α-syn pathology. Notably, previous studies have shown that HTRA1 also degrades other aggregation-prone proteins, including FUS, TDP-4 (27), and fibrillar tau (38, 39), raising the intriguing possibility that NALL may exert protective effects in other neurodegenerative disorders, such as amyotrophic lateral sclerosis, frontotemporal dementia, and Alzheimer’s disease.

A recent clinical study reported that acetyl-leucine reversed the loss of striatal DAT binding in the nigrostriatal system of patients with prodromal PD (21). However, the mechanisms underlying these effects were not defined. Our results suggested that NALL may enhance synaptic function by increasing parkin levels and stabilizing glycosylated DAT in PD patient–derived dopaminergic neurons. This aligns with previous studies indicating that parkin facilitates DAT surface expression by ubiquitinating and degrading misfolded, nonglycosylated DAT (30).

Emerging evidence suggests that parkin plays a crucial role in synaptic function, particularly in the regulation of synaptic vesicle recycling (31, 40). Neuronal activity-dependent activation of parkin in human dopaminergic neurons leads to ubiquitination of SYNJ1, facilitating its interaction with endophilin A1 and promoting synaptic vesicle endocytosis (31). In agreement with these findings, NALL-mediated increase in parkin expression enhanced the localization of SYNJ1 at synaptic membrane and improved synaptic vesicle endocytosis, suggesting that NALL may facilitate dopamine neurotransmission. This notion is supported by our findings in *LRRK2*^R1441C^ knockin mice that exhibit impaired dopamine-dependent motor learning (34), indicating striatal dopamine signaling dysfunction. The treatment of the *LRRK2*^R1441C^ mice with NALL led to upregulation of parkin in nigral dopaminergic neurons and improvements in their motor learning performance. Given the known effects of parkin (31), these findings suggest that NALL-mediated parkin upregulation may help restore synaptic vesicle dynamics (41, 42) and DAT function in *LRRK2*-mutant neurons (43, 44). While *PRKN* mutations are relatively uncommon, parkin function is reduced in sporadic PD as well, via several mechanisms, including nitrosylation, oxidation, phosphorylation, and aggregation (45). Furthermore, overexpression of parkin improves neuronal survival (46–48), while reducing parkin levels favors cell death (49). Therefore, interventions that upregulate parkin may promote neuronal survival and could serve as targets for therapeutic intervention in PD.

Although *LRRK2* mutations, including R1441C, are not typically associated with early-onset PD, the *LRRK2*^R1441C^ knockin mouse model (34, 50, 51) provides a valuable in vivo model for studying the early-stage pathogenic mechanisms of PD, as they display dopamine-related functional impairments preceding overt dopaminergic neuron loss. The restoration of dopamine-dependent striatal motor learning by NALL likely reflects the correction of dopaminergic circuit dysfunctions similar to those present in asymptomatic *LRRK2* mutation carriers (52, 53). Although the doses of both dopamine antagonists were relatively high and not fully selective for D1 or D2 receptors, this design was intentional. Given the critical role of dopamine in motor learning, and previous studies indicating that dopamine receptor blockade during skill acquisition impairs subsequent performance, we aimed to use high doses to create a condition of significant, broad dopaminergic signaling dysfunction — regardless of receptor specificity — that could also be reversible. This experimental design allowed us to evaluate the effects of NALL under conditions of severe dopamine receptor blockade. A recent work focusing on a progressive PD model provides additional evidence linking dopamine-dependent motor learning deficits with early synaptic dopamine neuron dysfunctions during PD progression (54). This study found that decreased dopamine release from dopaminergic axons results in striatal motor learning deficits in the absence of PD motor impairments, which are only observed upon a significant loss of dopamine neurons. These findings further support the potential neuroprotective effects of NALL that have been reported in a clinical setting (21). In addition, we found a reduction of pS129-syn in human dopaminergic neurons carrying *LRRK2* mutation and in *LRRK2*-mutant mice, further supporting the role of NALL in enhancing α-syn clearance mechanisms.

Our proteomic results indicate that NALL treatment of human dopaminergic neurons primarily increased the level of mitochondrial, synaptic, and lysosomal proteins. While there could be multiple mechanisms underlying this observation, it is also possible that NALL-mediated elevation of parkin level, at least in part, contributed to these proteomic changes. It has been well-established that parkin plays an important role in the mitochondrial quality control pathway (55–58), as well as in lysosomal function by regulating direct contacts and transfer of amino acids between lysosomes and mitochondria (59). It is also possible that high levels of intracellular leucine affect mTOR function (60) and autophagy (61, 62) that in turn impact mitochondrial and lysosomal function. Further mechanistic studies will be required to address these questions.

NALL appears to increase HTRA1 expression in PD models, likely through transcriptional regulation. In contrast, NALL treatment did not elevate HTRA1 levels or reduce pS129-syn in wild-type mice, suggesting that its effects on HTRA1 and pS129-syn are context dependent. One possible explanation is that neurons harboring PD-related mutations experience higher baseline cellular stress, rendering them more responsive to NALL-induced HTRA1 upregulation. However, further mechanistic studies are needed to clarify these pathways.

Finally, considering translational relevance, NALL has already been evaluated clinically and received FDA approval for NPC1, confirming a favorable safety profile in humans. While our study focused on elucidating cellular mechanisms rather than pharmacokinetics, the in vivo NALL dose (100 mg/kg/d) was chosen based on prior studies showing central nervous system exposure and behavioral efficacy in lysosomal storage disease models (16). Clinical dosing (2–5 g/d, ~0.1 g/kg/d) has demonstrated symptomatic improvement and dopaminergic imaging benefits (20, 21, 63, 64), suggesting adequate CNS bioavailability. Although the effective in vitro concentrations (5–10 mM) are higher than estimated brain exposures in vivo, these experiments are intended to define the cellular capacity and underlying mechanisms of NALL action rather than to model absolute exposure levels. Importantly, oral NALL treatment at 100 mg/kg/d in *LRRK2*-mutant mice produced changes — reduced pS129-syn and increased parkin and insoluble HTRA1 — that closely mirrored the responses observed in human iPSC-derived neurons. This cross-system consistency suggests that, despite the higher nominal in vitro dose, the key pathways NALL engages are conserved in vivo, supporting the biological relevance of the mechanisms identified in cell-based assays. Together, our findings support NALL as a promising therapeutic candidate for PD, by targeting α-syn and presynaptic terminals.

## Methods

### Sex as a biological variable

Our study included both male and female animals, and similar findings were reported for both sexes.

### Cells and animals

Human iPSC *GBA1* L444P line was a gift from Thomas Gasser, Hertie Institute for Clinical Brain Research, University of Tübingen; German Center for Neurodegenerative Diseases (DZNE), Tübingen, Germany (65); *GBA1* N370S line, *LRRK2* R1441C line, *PRKN* A324fsX110 line, and the sporadic PD line were from the Northwestern University Biorepository; *LRRK2* G2019S line was generated from the KOLF line (Jax: JIPSC001000); and *VPS35* line was a gift from Christine Klein, Institute of Neurogenetics, University of Lübeck and University Hospital Schleswig-Holstein, Lübeck, Germany (66, 67). The α-syn stable-expression SH-SY5Y cell line was a gift from Joe Mazzulli, Ken and Ruth Davee Department of Neurology, Northwestern University Feinberg School of Medicine. SH-SY5Y cells were grown in DMEM/F12 with 10% FBS media and transfected with HTRA1 plasmid (GenScript) using the Neon electroporation system, following the manufacturer’s instructions. Three-month-old C57BL/6 (Jax 000664, RRID:IMSR_JAX:000664) and homozygous *LRRK2*^R1441C^ knockin mice (RRID:IMSR_JAX:009346) were group-housed on a standard 12-hour light/dark cycle with standard feeding. Littermates were randomly assigned to the experimental procedures. Both males and females were used.

### iPSC culture and neuronal differentiation

Human iPSCs were cultured and maintained as described previously (68). Directed differentiation toward dopaminergic neurons was conducted as described previously (68–71). Briefly, undifferentiated iPSCs were plated onto Cultrex-coated (BD Biosciences) 6-well plates in mTeSR media (STEMCELL Technologies). Differentiation was started by adding knockout serum replacement medium (Invitrogen) containing Noggin (R&D Systems) and SB431542 (Tocris Bioscience). To help control neuralization variability, cells were passaged by chunking manually (en bloc: size of 1–2 mm) and plated onto 10 cm dishes precoated with poly-d-lysine (Sigma) and laminin (Roche) on day 13. Differentiation into dopaminergic neurons was conducted by adding neuralization growth factors. All the experiments were done between day 80 and day 150, unless otherwise specified. For NALL (Sigma, 441511) treatment, NALL was dissolved in DMSO at 1 M for stock and diluted into cell culture media to get final concentrations as indicated in the figures. Neurons were treated with different concentrations of NALL twice a week for the indicated days. Both 0 mM NALL and nontreated (NT) groups were treated with DMSO at the same concentration of the 10 mM NALL group.

### Mouse motor learning

NALL was dissolved in ethanol to prepare 50 mg/mL solution, which was then diluted in water to get 10 mg/mL. A total of 0.25 mL of NALL solution (10 mg/mL) was orally administered in mice at 100 mg/kg/d dose (2.5 mg NALL/25 g mouse) (72). Motor learning was assessed using an accelerating rotarod with 8- to 9-week-old *LRRK2*^R1441C^ mice. The rotarod apparatus (Panlab) is equipped with a mouse rod (3 cm diameter) and set to 4–40 rpm acceleration over 300 sec. The task consisted of 18 daily sessions (5 trials per session; intertrial interval = 15 s, max trial duration = 300 s) divided into 2 phases. In the first phase — sessions 1–5 — mice were administered either NALL or vehicle via oral gavage 30 min before an i.p. injection of a cocktail of D1 receptor (SCH23390, Sigma D054) and D2 receptor (eticlopride, Sigma E101). Each antagonist was administered a dose of 1 mg/kg for 30 min. After a 72-hour break, the mice were tested over 13 sessions without dopamine antagonists. Instead, they received either NALL or vehicle via oral gavage 60 minutes before the rotarod test.

### Lentivirus

Lentiviral shRNA constructs (MISSION pLKO.1-puro) for nontarget control (scramble) and HTRA1 were obtained from MilliporeSigma. Lentiviral synaptophysin-pHluorin–expressing construct was a gift from Volker Haucke, Leibniz-Forschungsinstitut für Molekulare Pharmakologie (FMP), Berlin, Germany. For lentiviral packaging, human embryonic kidney (HEK) 293FT cells (Thermo Fisher Scientific) were transfected with lentiviral expression constructs together with psPAX2 (Addgene, 12260) and pLP3 (Invitrogen) using X-tremeGENE HP DNA Transfection Reagent (Roche). A media change was performed the day after transfection. At 48 hours after transfection, virus-containing supernatant was collected and cleared by centrifuge at 500*g* for 10 min. Virus particles were concentrated using Lenti-X Concentrator (Takara Bio, 631232) following manufacturer’s instructions. The concentration of virus particles was determined by ELISA using RETRO-TEK HIV-1 p24 Antigen ELISA Kit (ZeptoMetrix, 0801111). Neurons were transfected with lentivirus at an MOI of 5 at day 60 or day 90 postdifferentiation for 30 days.

### Western blot analysis

Cells were scraped in cold PBS and centrifuged at 300*g* for 5 min. Pellets were resuspended in Triton buffer (containing 1% Triton X-100) with Halt Protease and Phosphatase Inhibitor Cocktail (Thermo Fisher Scientific) and incubated on ice for 30 min followed by homogenization for 2 min. Lysates were cleared by centrifugation at 100,000*g* for 30 min. Protein concentration of the supernatants, as Triton-soluble fraction, was measured using the Bicinchoninic Acid (BCA) assay (Sigma). Pellets were resolved in 2% SDS buffer, followed by sonication and centrifugation at 150,000*g* for 30 min. The supernatant was the Triton-insoluble fraction. Both Triton-soluble and insoluble fractions were denatured by heating in 4× Laemmli sample buffer (Bio-Rad). Equal amounts of protein were loaded in 4%–20% Tris-glycine gels. Proteins were transferred onto Nitrocellulose membrane using the Transblot Turbo transfer system (Bio-Rad). Membranes were blocked with 5% milk in TBS-T (50 mM Tris at pH 7.4, 150 mM NaCl, 0.1% Tween 20) and incubated with primary antibody overnight at 4°C. After washing, membranes were incubated with anti-rabbit or anti-mouse secondary antibody for 1 hour at room temperature. Chemiluminescence was assessed using pico or femto chemiluminescence substrates (Thermo Fisher Scientific). ChemiDoc XRS + imaging station with a 16-bit CCD camera was used for imaging. Quantification was done using ImageJ software (NIH). Neuron-enriched proteins, such as α-syn, pS129-syn, and DAT, were normalized with neuronal marker β-III-tubulin. Other proteins were normalized with GAPDH, unless otherwise specified. Antibodies used for immunoblotting include anti-pS129-syn antibody (Cell Signaling Technology: 23706S), anti-SYP antibody (MilliporeSigma: ab9272), anti-α-syn antibody (Santa Cruz Biotechnology: sc-7011-R), anti-β-III-tubulin antibody (BioLegend: 801202), anti-SYNJ1 antibody (LsBio: 65169), anti-parkin antibody (Santa Cruz Biotechnology: sc-32282), anti-HTRA1 (R&D Systems: MAB2916), anti-HTRA1 (Proteintech: 55011-1-AP), anti-caspase 3 (Cell Signaling Technology: 9662), anti-cleaved caspase-3 (Cell Signaling Technology: 9661S), anti-DAT antibody (Santa Cruz Biotechnology: sc-32259), anti-GAPDH antibody (MilliporeSigma: MAB374), Goat Anti-Rat IgG (H+L) (Peroxidase AffiniPure: 112-035-062), Goat Anti-Rabbit IgG (H+L) (Peroxidase-AffiniPure: 111-035-144), and Goat Anti-Mouse IgG (H+L) (Peroxidase-AffiniPure: 115-035-146).

### Synaptic membrane enrichment assay

Cells grown in 6-well plates were washed with cold PBS and scraped in Syn-PER Synaptic Protein Extraction Reagent (Syn-PER) (Thermo Fisher Scientific) followed by centrifuging at 1,200*g* for 10 min at 4°C. Supernatants were collected as homogenates (total) and further centrifuged at 15,000*g* for 20 min at 4°C. The supernatants from the second spin contained cytosolic proteins (cyto). The pellets were resolubilized in Syn-PER and centrifuged at 70,000*g* for 45 min at 4°C. The supernatants from this spin were added into the cyto fraction, and the pellets were washed with Syn-PER buffer twice and finally resuspended in Syn-PER as the synaptic membrane fraction, which contained synaptic vesicle membrane and synaptic plasma membrane. Equal amounts of protein from each fraction were loaded and detected by Western blot using indicated antibodies.

### Label-free quantitative proteomics

#### Sample preparation.

iPSC-derived midbrain dopaminergic neurons were treated with 0, 5, or 10 mM of NALL twice a week for 4 weeks. Cells were harvested for label-free quantitative proteomic analysis. Briefly, neurons were harvested in 1× PBS and pelleted at 3,000*g* for 5 min at 4°C. RIPA buffer containing complete protease inhibitor cocktail was added to the pellet at a volume of 1 mL of lysis buffer per 250 μL cell pellet volume. Samples were homogenized using a tip sonicator 3 times for 30 sec with rests of 1 min in between. Lysates were centrifuged at 10,000*g* for 10 min at 4°C. Supernatant was collected for protein quantification using the BCA assay. Protein pellets were resuspended in 8 M urea (Thermo Fisher Scientific, catalog 29700) prepared in 100 mM ammonium bicarbonate solution (Fluka, catalog 09830). DTT (DOT Scientific Inc, catalog DSD11000) was applied to a final concentration of 5 mM. After incubation at room temperature (RT) for 20 min, iodoacetamide (IAA, Sigma-Aldrich, catalog I1149) was added to a final concentration of 15 mM and incubated for 20 min at RT in the dark. Excess IAA was quenched with DTT for 15 min. Samples were diluted with 100 mM ammonium bicarbonate solution and digested for 3 hours with Lys-C protease (1:100, Thermo Fisher Scientific, catalog 90307_3668048707) at 37°C. Trypsin (1:100, Promega, catalog V5280) was then added for overnight incubation at 37°C with intensive agitation (1,000 rpm). The next day, the reaction was quenched by adding 1% trifluoroacetic acid (Thermo Fisher Scientific, catalog O4902-100). The samples were desalted using Peptide Desalting Spin Columns (Thermo Fisher Scientific, catalog 89852). All samples were vacuum-centrifuged to dry.

#### TMT labeling.

Our protocol was based on previously reported methods (73–75). C18 column–desalted peptides were resuspended with 100 mM HEPES pH 8.5, and the concentrations were measured by micro BCA kit (Thermo Fisher Scientific, catalog PI23235). For each sample, 100 mg of peptide was labeled with TMT reagent (0.4 mg, dissolved in 40 mL anhydrous acetonitrile, Thermo Fisher Scientific, catalog A44520) and made at a final concentration of 30% (v/v) acetonitrile (ACN). Following incubation at RT for 2 hours with agitation, hydroxylamine (to a final concentration of 0.3% [v/v]) was added to quench the reaction for 15 min. TMT-tagged samples were mixed at a 1:1:1:1:1:1:1:1:1 ratio. Combined sample was vacuum-centrifuged to dryness, resuspended, and subjected to Peptide Desalting Spin Columns (Thermo Fisher Scientific, catalog 89852).

#### Peptide fractionation.

We used a high-pH reverse-phase peptide fractionation kit (Thermo Fisher Scientific, catalog 84868) to get 8 fractions (10.0%, 12.5%, 15.0%, 17.5%, 20.0%, 22.5%, 25.0%, and 50% of ACN in 0.1% triethylamine solution). The high-pH peptide fractions were directly loaded into the autosampler for MS analysis without further desalting.

#### Tandem MS.

Two micrograms of each fraction or sample were autosampler-loaded with a Thermo Fisher Scientific Vanquish Neo UHPLC system onto a PepMap Neo Trap Cartridge (Thermo Fisher Scientific, 174500; diameter, 300 μm; length, 5 mm; particle size, 5 μm; pore size, 100 Å; stationary phase, C18) coupled to a nanoViper analytical column (Thermo Fisher Scientific, 164570; diameter, 0.075 mm; length, 500 mm; particle size, 3 μm; pore size, 100 Å; stationary phase, C18) with a stainless-steel emitter tip assembled on the Nanospray Flex Ion Source with a spray voltage of 2,000 V. An Orbitrap Ascend (Thermo Fisher Scientific) was used to acquire all the MS spectral data. Buffer A contained 99.9% H_2_O and 0.1% formic acid, and buffer B contained 80.0% ACN, 19.9% H_2_O, with 0.1% formic acid. For each fraction, the chromatographic run was for 4 hours in total with the following profile: 0%–7% for 7, 10% for 6, 25% for 160, 33% for 40, 50% for 7, 95% for 5, again 95% for 15 min, respectively.

We used a multiNotch MS^3^-based TMT method to analyze all the TMT samples (75–77). The scan sequence began with an MS^1^ spectrum (Orbitrap analysis, resolution 120,000, 400–1,400 Th, automatic gain control target 2 × 10^5^, maximum injection time 200 ms). The parameters for MS^2^ analysis were “top speed” (2 s), collision-induced dissociation (quadrupole ion trap analysis, automatic gain control 4 × 10^3^, normalized collision energy 35, maximum injection time 150 ms). The parameters for MS^3^ analysis were top 10 precursors, fragmented by higher-energy collisional dissociation prior to Orbitrap analysis (normalized collision energy 55, max automatic gain control 5 × 10^4^, maximum injection time 250 ms, isolation specificity 0.5 Th, resolution 60,000).

#### MS data analysis and quantification.

Protein identification/quantification and analysis were performed with Integrated Proteomics Pipeline - IP2 using ProLuCID (78, 79), DTASelect2 (80, 81), and Census and Quantitative Analysis (for TMT-MS experiments). Spectrum raw files were extracted into MS1, MS2, and MS3 (for TMT experiments) files using RawConverter (http://fields.scripps.edu/downloads.php). The tandem mass spectra were searched against UniProt human (downloaded on January 1, 2014) protein databases (82) and matched to sequences using the ProLuCID/SEQUEST algorithm (ProLuCID version 3.1) with 5 ppm peptide mass tolerance for precursor ions and 600 ppm for fragment ions. The search space included all fully and half-tryptic peptide candidates within the mass tolerance window with no-miscleavage constraint, assembled, and filtered with DTASelect2 through IP2. To estimate peptide probabilities and FDRs accurately, we used a target/decoy database containing the reversed sequences of all the proteins appended to the target database (82). Each protein identified was required to have a minimum of 1 peptide of minimal length of 6 amino acid residues; however, this peptide had to be an excellent match with an FDR < 1% and at least 1 excellent peptide match. After the peptide/spectrum matches were filtered, we estimated that the peptide FDRs were ≤1% for each sample analysis. Resulting protein lists include subset proteins to allow for consideration of all possible protein forms implicated by at least 2 given peptides identified from the complex protein mixtures. Then, we used Census and Quantitative Analysis in IP2 for protein quantification of TMT-MS experiments, and protein quantification was determined by summing all TMT report ion counts. For TMT experiments, we used static modification: 57.02146 C for carbamidomethylation, 304.2071 for 16-plex TMT tagging; differential modifications: 304.2071 for N-terminal 16-plex TMT tagging, 42.0106 for N-terminal acetylation. Resulting protein lists include subset proteins to allow for consideration of all possible protein isoforms implicated by at least 2 given peptides identified from the complex protein mixtures. Each possible protein isoform was quantified individually (83).

Spyder (MIT, Python 3.7, libraries, ‘pandas,’ ‘numpy,’ ‘scipy,’ ‘statsmodels,’ ‘bioinfokit’) was used for data statistical analyses. RStudio (version, 2024.09.1 Build 394, packages, ‘tidyverse,’ ‘pheatmap’) was used for data virtualization. The Database for Annotation, Visualization and Integrated Discovery was used for protein functional annotation analysis.

### pHluorin fluorescence

#### Live imaging.

Neurons grown on glass coverslips were transfected with synaptophysin-pHluorin–expressing lentivirus for 4 weeks, and cells were treated with 0 or 10 mM of NALL for 2 weeks before the experiment. Cells were transferred to an imaging chamber equipped with field stimulation (RC-49MFSH, Warner Instruments) and mounted on an inverted epifluorescence microscope (Nikon TE300) with a 40×/1.35 NA oil-immersion objective. The imaging chamber was perfused with artificial cerebrospinal fluid containing the following: 125 mM NaCl, 3 mM KCl, 1.25 mM NaH_2_PO_4_, 25 mM NaHCO_3_, 1 mM MgCl_2_, 2 mM CaCl_2_, 25 mM D(+)-glucose, pH 7.4, osmolality 310 mOsm/L, at a flow rate of 0.5 mL/min. Recording chamber temperature was maintained at 31°C–33°C. pHluorin fluorescence was excited at 475–485 nm by Polychrome V (TILL Photonics), and emissions were captured with a cooled CCD camera (Hamamatsu ImagEM) controlled by Slidebook imaging software (Intelligent Imaging Innovations), with a 495dcxru dichroic mirror and a 530/50 emission filter (Chroma). Time-lapse images (512 × 512 pixels) of pHluorin fluorescence were captured at 1 Hz. Each capture consisted of an average of two 50 ms exposures. Electrical field stimulation was generated by a constant current isolated stimulator (Digitimer DS3) controlled by a pulse stimulator (AMPI). A 10-second, 20 Hz train of 1 ms, 20 mA pulse was delivered to evoke exocytosis at the synapses.

#### Image analysis.

Time-lapse images were deconvolved using Slidebook software. Active synapses were identified by comparing images immediately after and before a stimulus train. Ellipse ROIs encompassing the active synapses were then drawn and analyzed. For each ROI, baseline fluorescence calculated by averaging the 10 frames immediately before stimulus was subtracted for all time points. Peak pHluorin fluorescence intensity following stimulation was identified using a 3-frame window. To calculate the recovery time constant of pHluorin fluorescence, ROI data from peak intensity to the end of recording were imported into GraphPad Prism software and fitted (least squares regression) with a 1-phase decay model with plateau constrained to 0.

### Seahorse respirometry

Neurons were plated at full confluence on XF24 microplates (Agilent 100777-004). Cells were treated with 0 (DMSO only) or 10 mM of NALL for 10 days before analysis, with 6 technical replicates performed for each biological replicate of the experiment. Experiments were conducted on day 60 differentiated neurons, before any signs of neuronal clumping appeared. The night before respirometry measurements, an XF24 Extracellular Flux Assay Kit (Agilent 100850-001) was equilibrated with 1 mL XF Calibrant (Agilent 100840-000) in each well at 37°C without CO_2_. The following morning, 50 mL Seahorse medium was prepared using XF DMEM (Agilent 103575-100), supplemented with 10 mM glucose (Agilent 103577-100), 1 mM pyruvate (Gibco 11360-070), and 2 mM l-glutamine (Gibco 25030-081), with pH adjusted to 7.4. After heating to 37°C, the Seahorse medium was used for 1 wash (800 μL) and medium replacement (500 μL) of the neurons, which were then incubated at 37°C for 1 hour without CO_2_. While incubating, a Mito Stress Test assay was initiated on a Seahorse XF24 Analyzer (Agilent 100736) and calibrated with the Extracellular Flux Assay Kit. The Mito Stress Test assay was then performed, using final concentrations of 2 μM oligomycin, 2 μM FCCP, and 0.75 μM each of antimycin A and rotenone, freshly prepared from an XF Cell Mito Stress Test Kit (Agilent 103015-100).

After respirometry measurements, protein content for each technical replicate was measured by gently removing the medium from each well and resuspending the contents in 30 μL RIPA buffer. Samples were then stored for 10 minutes at –80°C, thawed, transferred to Eppendorf tubes, and centrifuged for 18,000*g* for 10 min at 4°C. Supernatant was collected from the tubes, and total protein content was measured by BCA assay (Pierce 23225), using a Molecular Devices SpectraMax i3 plate reader with SoftMax Pro 6.3. Software used in collecting and analyzing the respirometry data included XFReader (v1.8.1.1), Wave (v2.6.3.5), and R (v4.5.1).

### Statistics

Statistical analyses were performed using GraphPad Prism 9. Two-group comparisons were conducted using 2-tailed *t* tests. For datasets with more than 2 groups, 1-way ANOVA followed by Tukey’s post hoc test was applied unless otherwise specified. For multiple comparisons, each group was compared with the designated control group (0 mM, NT, or vehicle, unless indicated). Behavior tests were analyzed using 2-way repeated measures ANOVA followed by Tukey’s post hoc test. *P* < 0.05 was considered statistically significant.

### Study approval

All research complied with regulatory committees for research safety (Safety Protocol ID: BIO20230025). All mouse experiments were performed in compliance with Northwestern University Animal Care and Use Committee guidelines. Northwestern University Institutional Animal Care and Use Committee (Chicago, Illinois, USA) approved the study.

### Data availability

The MS data presented in this study were deposited in Mass Spectrometry Interactive Virtual Environment under the identifier MSV000097594 (https://massive.ucsd.edu/ProteoSAFe/dataset.jsp?task=67a299d5b94249d888c48b1cfb0cff4a) and ProteomeXchange under the identifier PXD062821 (https://proteomecentral.proteomexchange.org/cgi/GetDataset?ID=PXD062821).

Values for all data points represented in graphs and reported as means throughout the manuscript and supporting material are included in the Supporting Data Values file.

## Author contributions

PS and DK designed the overall study; PS performed all neuronal experiments with the assistance of BD and analyzed the data, except for LRRK2-mutant neuron experiments that were performed and analyzed by RF and MB; LP and CC performed and analyzed LRRK2 mouse behavioral experiments; YZW and JNS performed proteomics and analyzed the proteomic data; ZX and DJS performed and analyzed pHluorin experiments; RC performed and analyzed Seahorse experiments; YZ provided the VPS35 neurons; and PS and DK wrote the manuscript with input from all authors.

## Funding support

This work is the result of NIH funding, in whole or in part, and is subject to the NIH Public Access Policy. Through acceptance of this federal funding, the NIH has been given a right to make the work publicly available in PubMed Central.

- National Institutes of Health grants R37 NS096241
- and R35 NS122257 (to DK) and S10 OD032464 (to JNS).

## Supplementary Material

Supplemental data

Unedited blot and gel images

Supplemental table 1

Supplemental table 2

Supporting data values

## Version 1 03/02/2026

Electronic publication

## References

[1] Klein C, Krainc D (2012). Movement disorders in 2011: translating new research findings into clinical practice. Nat Rev Neurol.

[2] Surmeier DJ (2017). Selective neuronal vulnerability in Parkinson disease. Nat Rev Neurosci.

[3] Nguyen M (2019). Synaptic, mitochondrial, and lysosomal dysfunction in Parkinson’s Disease. Trends Neurosci.

[4] Spillantini MG (1997). Alpha-synuclein in Lewy bodies. Nature.

[5] Chen L (2009). Tyrosine and serine phosphorylation of alpha-synuclein have opposing effects on neurotoxicity and soluble oligomer formation. J Clin Invest.

[6] Fujiwara H (2002). alpha-Synuclein is phosphorylated in synucleinopathy lesions. Nat Cell Biol.

[7] Smith WW (2005). Alpha-synuclein phosphorylation enhances eosinophilic cytoplasmic inclusion formation in SH-SY5Y cells. J Neurosci.

[8] Rogers JT (2011). The alpha-synuclein 5’untranslated region targeted translation blockers: anti-alpha synuclein efficacy of cardiac glycosides and Posiphen. J Neural Transm (Vienna).

[9] Sen S, West AB (2009). The therapeutic potential of LRRK2 and alpha-synuclein in Parkinson’s disease. Antioxid Redox Signal.

[10] Scherzer CR (2008). GATA transcription factors directly regulate the Parkinson’s disease-linked gene alpha-synuclein. Proc Natl Acad Sci U S A.

[11] Bremova T (2015). Acetyl-dl-leucine in Niemann-Pick type C: a case series. Neurology.

[12] Vibert N, Vidal PP (2001). In vitro effects of acetyl-DL-leucine (tanganil) on central vestibular neurons and vestibulo-ocular networks of the guinea-pig. Eur J Neurosci.

[13] van Gool R (2025). Levacetylleucine (N-acetyl-l-leucine) for Niemann-Pick disease type C. Trends Pharmacol Sci.

[14] Martakis K (2023). Efficacy and safety of N-Acetyl-l-leucine in children and adults with GM2 gangliosidoses. Neurology.

[15] Kaya E (2021). Acetyl-leucine slows disease progression in lysosomal storage disorders. Brain Commun.

[16] Kaya E (2020). Beneficial effects of Acetyl-DL-leucine (ADLL) in a mouse model of sandhoff disease. J Clin Med.

[17] Strupp M (2013). Effects of acetyl-DL-leucine in patients with cerebellar ataxia: a case series. J Neurol.

[18] Schniepp R (2016). Acetyl-DL-leucine improves gait variability in patients with cerebellar ataxia-a case series. Cerebellum Ataxias.

[19] Martakis K (2025). N-Acetyl-leucine in progressive CACNA1A ataxia: a case series. Eur J Paediatr Neurol.

[20] Fields T (2021). Acetyl-DL-leucine improves restless legs syndrome: a case report. J Neurol.

[21] Oertel WH (2024). Acetyl-DL-leucine in two individuals with REM sleep behavior disorder improves symptoms, reverses loss of striatal dopamine-transporter binding and stabilizes pathological metabolic brain pattern-case reports. Nat Commun.

[22] Gunther L (2015). N-acetyl-L-leucine accelerates vestibular compensation after unilateral labyrinthectomy by action in the cerebellum and thalamus. PLoS One.

[23] Tighilet B (2015). Comparative analysis of pharmacological treatments with N-acetyl-DL-leucine (Tanganil) and its two isomers (N-acetyl-L-leucine and N-acetyl-D-leucine) on vestibular compensation: Behavioral investigation in the cat. Eur J Pharmacol.

[24] Mazzulli JR (2011). Gaucher disease glucocerebrosidase and α-synuclein form a bidirectional pathogenic loop in synucleinopathies. Cell.

[25] Anderson JP (2006). Phosphorylation of Ser-129 is the dominant pathological modification of alpha-synuclein in familial and sporadic Lewy body disease. J Biol Chem.

[26] Kahle PJ (2000). Physiology and pathophysiology of alpha-synuclein. Cell culture and transgenic animal models based on a Parkinson’s disease-associated protein. Ann N Y Acad Sci.

[27] Chen S (2024). HTRA1 disaggregates α-synuclein amyloid fibrils and converts them into non-toxic and seeding incompetent species. Nat Commun.

[28] Khazeem MM (2022). TOP2B is required to maintain the adrenergic neural phenotype and for ATRA-induced differentiation of SH-SY5Y neuroblastoma cells. Mol Neurobiol.

[29] Li LB (2004). The role of N-glycosylation in function and surface trafficking of the human dopamine transporter. J Biol Chem.

[30] Jiang H (2004). Parkin increases dopamine uptake by enhancing the cell surface expression of dopamine transporter. J Biol Chem.

[31] Song P (2023). Parkinson’s disease-linked parkin mutation disrupts recycling of synaptic vesicles in human dopaminergic neurons. Neuron.

[32] Sankaranarayanan S, Ryan TA (2000). Real-time measurements of vesicle-SNARE recycling in synapses of the central nervous system. Nat Cell Biol.

[33] Villarreal S Measuring synaptic vesicle endocytosis in cultured hippocampal neurons. J Vis Exp.

[34] Xenias HS (2022). R1441C and G2019S LRRK2 knockin mice have distinct striatal molecular, physiological, and behavioral alterations. Commun Biol.

[35] Beeler JA (2012). A role for dopamine-mediated learning in the pathophysiology and treatment of Parkinson’s disease. Cell Rep.

[36] Zhuang X (2013). The role of neuroplasticity in dopaminergic therapy for Parkinson disease. Nat Rev Neurol.

[37] Koranda JL (2016). Chronic nicotine mitigates aberrant inhibitory motor learning induced by motor experience under dopamine deficiency. J Neurosci.

[38] Poepsel S (2015). Determinants of amyloid fibril degradation by the PDZ protease HTRA1. Nat Chem Biol.

[39] Tennstaedt A (2012). Human high temperature requirement serine protease A1 (HTRA1) degrades tau protein aggregates. J Biol Chem.

[40] Jiang H (2012). Parkin controls dopamine utilization in human midbrain dopaminergic neurons derived from induced pluripotent stem cells. Nat Commun.

[41] Nguyen M, Krainc D (2018). LRRK2 phosphorylation of auxilin mediates synaptic defects in dopaminergic neurons from patients with Parkinson’s disease. Proc Natl Acad Sci U S A.

[42] Pan PY (2017). Parkinson’s disease-associated LRRK2 hyperactive kinase mutant disrupts synaptic vesicle trafficking in ventral midbrain neurons. J Neurosci.

[43] Longo F (2017). Age-dependent dopamine transporter dysfunction and Serine129 phospho-α-synuclein overload in G2019S LRRK2 mice. Acta Neuropathol Commun.

[44] Volta M (2017). Initial elevations in glutamate and dopamine neurotransmission decline with age, as does exploratory behavior, in LRRK2 G2019S knock-in mice. Elife.

[45] Dawson TM, Dawson VL (2014). Parkin plays a role in sporadic Parkinson’s disease. Neurodegener Dis.

[46] Petrucelli L (2002). Parkin protects against the toxicity associated with mutant alpha-synuclein: proteasome dysfunction selectively affects catecholaminergic neurons. Neuron.

[47] Wang DB (2013). Declines in Drp1 and parkin expression underlie DNA damage-induced changes in mitochondrial length and neuronal death. J Neurosci.

[48] Yasuda T (2011). Parkin-mediated protection of dopaminergic neurons in a chronic MPTP-minipump mouse model of Parkinson disease. J Neuropathol Exp Neurol.

[49] Yang H (2007). Downregulation of parkin damages antioxidant defenses and enhances proteasome inhibition-induced toxicity in PC12 cells. J Neuroimmune Pharmacol.

[50] Chen C (2020). Pathway-specific dysregulation of striatal excitatory synapses by LRRK2 mutations. Elife.

[51] Tong Y (2009). R1441C mutation in LRRK2 impairs dopaminergic neurotransmission in mice. Proc Natl Acad Sci U S A.

[52] Nandhagopal R (2008). Progression of dopaminergic dysfunction in a LRRK2 kindred: a multitracer PET study. Neurology.

[53] Simuni T (2020). Clinical and dopamine transporter imaging characteristics of non-manifest LRRK2 and GBA mutation carriers in the Parkinson’s Progression Markers Initiative (PPMI): a cross-sectional study. Lancet Neurol.

[54] Cornejo-Olivas M (2022). Disruption of mitochondrial complex I induces progressive Parkinsonism. Mov Disord.

[55] Wang H (2011). Parkin ubiquitinates Drp1 for proteasome-dependent degradation: implication of dysregulated mitochondrial dynamics in Parkinson disease. J Biol Chem.

[56] Shin JH (2011). PARIS (ZNF746) repression of PGC-1α contributes to neurodegeneration in Parkinson’s disease. Cell.

[57] Wang X (2011). PINK1 and Parkin target Miro for phosphorylation and degradation to arrest mitochondrial motility. Cell.

[58] Narendra D (2008). Parkin is recruited selectively to impaired mitochondria and promotes their autophagy. J Cell Biol.

[59] Peng W (2023). Parkin regulates amino acid homeostasis at mitochondria-lysosome (M/L) contact sites in Parkinson’s disease. Sci Adv.

[60] Zhang Y (2007). Increasing dietary leucine intake reduces diet-induced obesity and improves glucose and cholesterol metabolism in mice via multimechanisms. Diabetes.

[61] Klionsky DJ, Emr SD (2000). Autophagy as a regulated pathway of cellular degradation. Science.

[62] Yamamoto A (2006). Autophagy-mediated clearance of huntingtin aggregates triggered by the insulin-signaling pathway. J Cell Biol.

[63] Bremova-Ertl T (2024). Trial of *N*-Acetyl-l-Leucine in Niemann-pick disease type C. N Engl J Med.

[64] Fields T (2023). N-acetyl-L-leucine for Niemann-Pick type C: a multinational double-blind randomized placebo-controlled crossover study. Trials.

[65] Schondorf DC (2014). iPSC-derived neurons from GBA1-associated Parkinson’s disease patients show autophagic defects and impaired calcium homeostasis. Nat Commun.

[66] Kumar KR (2012). Frequency of the D620N mutation in VPS35 in Parkinson disease. Arch Neurol.

[67] Munsie LN (2015). Retromer-dependent neurotransmitter receptor trafficking to synapses is altered by the Parkinson’s disease VPS35 mutation p.D620N. Hum Mol Genet.

[68] Song P (2016). Parkin modulates endosomal organization and function of the endo-lysosomal pathway. J Neurosci.

[69] Kriks S (2011). Dopamine neurons derived from human ES cells efficiently engraft in animal models of Parkinson’s disease. Nature.

[70] Burbulla LF (2017). Dopamine oxidation mediates mitochondrial and lysosomal dysfunction in Parkinson’s disease. Science.

[71] Song P, Krainc D (2024). Protocol to investigate Parkinson’s patient-derived dopaminergic neurons by live-cell microscopy and oxidized dopamine assays. STAR Protoc.

[72] Hegdekar N (2021). N-Acetyl-L-leucine improves functional recovery and attenuates cortical cell death and neuroinflammation after traumatic brain injury in mice. Sci Rep.

[73] Wang YZ (2024). Notch receptor-ligand binding facilitates extracellular vesicle-mediated neuron-to-neuron communication. Cell Rep.

[74] Wang YZ (2024). Neuron type-specific proteomics reveals distinct Shank3 proteoforms in iSPNs and dSPNs lead to striatal synaptopathy in Shank3B^-/-^ mice. Mol Psychiatry.

[75] Weekes MP (2014). Quantitative temporal viromics: an approach to investigate host-pathogen interaction. Cell.

[76] McAlister GC (2014). MultiNotch MS3 enables accurate, sensitive, and multiplexed detection of differential expression across cancer cell line proteomes. Anal Chem.

[77] Ting L (2011). MS3 eliminates ratio distortion in isobaric multiplexed quantitative proteomics. Nat Methods.

[78] Eng JK (1994). An approach to correlate tandem mass spectral data of peptides with amino acid sequences in a protein database. J Am Soc Mass Spectrom.

[79] Xu T (2015). ProLuCID: an improved SEQUEST-like algorithm with enhanced sensitivity and specificity. J Proteomics.

[80] Cociorva D (2007). Validation of tandem mass spectrometry database search results using DTASelect. Curr Protoc Bioinformatics.

[81] Tabb DL (2002). DTASelect and Contrast: tools for assembling and comparing protein identifications from shotgun proteomics. J Proteome Res.

[82] UniProt C (2015). UniProt: a hub for protein information. Nucleic Acids Res.

[83] Raj B, Blencowe BJ (2015). Alternative splicing in the mammalian nervous system: recent insights into mechanisms and functional roles. Neuron.

