# Supplementary material for: N-acetyl-l-leucine lowers α-synuclein levels and improves synaptic function in Parkinson’s disease models
Source: J Clin Invest. 2026 Mar 2;136(5):e196137. doi: 10.1172/JCI196137 (PMC12948429; doi:10.1172/JCI196137)
Supplement: Supplemental data [file jci-136-196137-s170.pdf]

S_Figure 1

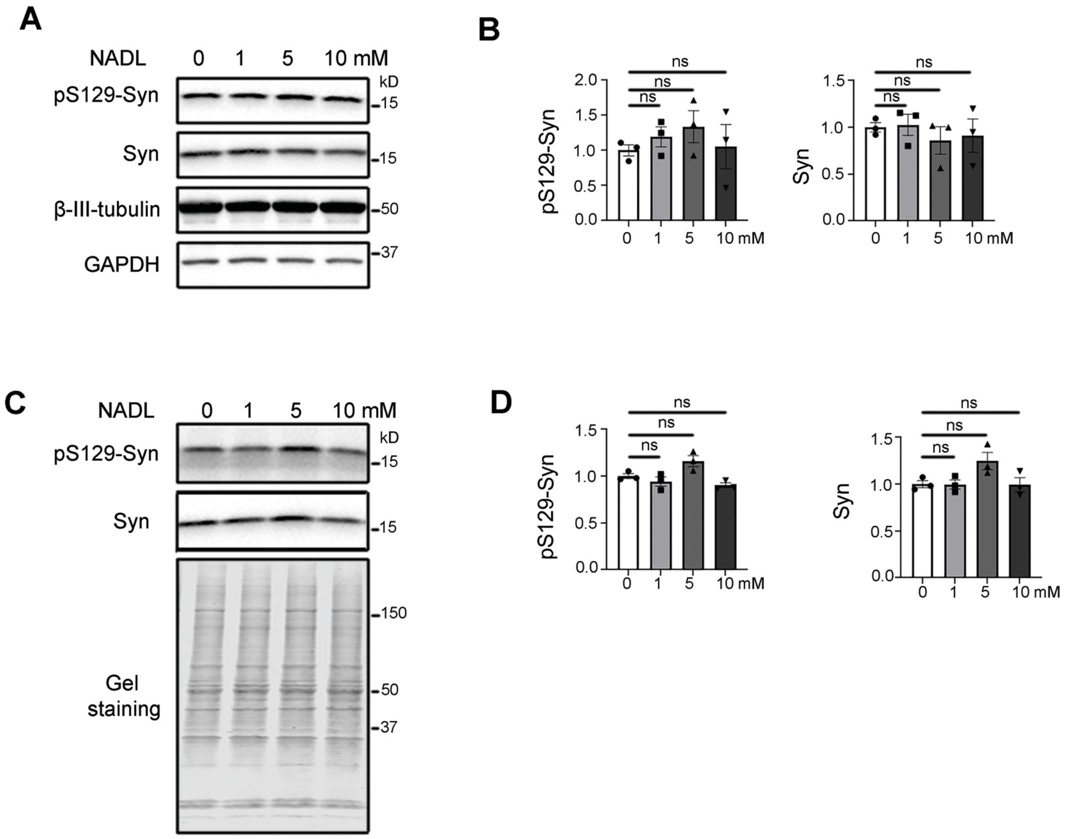
A
NADL 0 1 5 10 mM
kD
pS129-Syn
15
Syn
15
β-III-tubulin
50
GAPDH
37
B
pS129-Syn
ns
ns
ns
2.0
1.5
1.0
0.5
0.0
0 1 5 10 mM
Syn
1.5
1.0
0.5
0.0
C
NADL 0 1 5 10 mM
kD
pS129-Syn
15
Syn
15
Gel staining
150
50
37
D
pS129-Syn
Syn

S_Figure 1. NADL does not reduce pS129-syn levels in human dopaminergic neurons carrying GBA1 mutations.

(A) Representative Western blot showing pS129-syn and total α-syn (Syn) levels following 14 days of treatment with increasing concentrations of NADL in the Triton-soluble fraction of *GBA1* L444P mutant dopaminergic neurons. β-III-Tubulin and GAPDH served as loading controls.

(B) Quantification of pS129-syn (left) and total Syn (right) signals in (A), normalized to β-III-Tubulin and expressed relative to the 0 mM (DMSO) group (n = 3 independent experiments; One-way ANOVA).

(C) Western blot of pS129-syn and total Syn in the Triton-insoluble fraction of *GBA1* L444P mutant neurons following 14 days of NADL treatment; total protein staining served as a loading control.

(D) Quantification of pS129-syn (left) and total Syn (right) signals in (C), normalized to total protein and expressed relative to the 0 mM (DMSO) group (n = 3 independent experiments; One-way ANOVA).

ns, not significant.

S_Figure 2

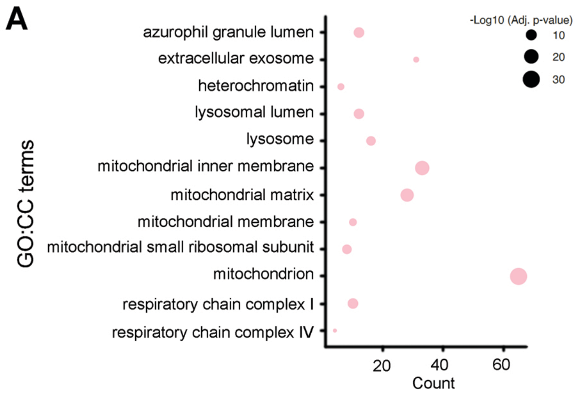
A
-Log10 (Adj. p-value)
10
20
30
azurophil granule lumen
extracellular exosome
heterochromatin
lysosomal lumen
lysosome
mitochondrial inner membrane
mitochondrial matrix
mitochondrial membrane
mitochondrial small ribosomal subunit
mitochondrion
respiratory chain complex I
respiratory chain complex IV
GO:CC terms
20
40
60
Count

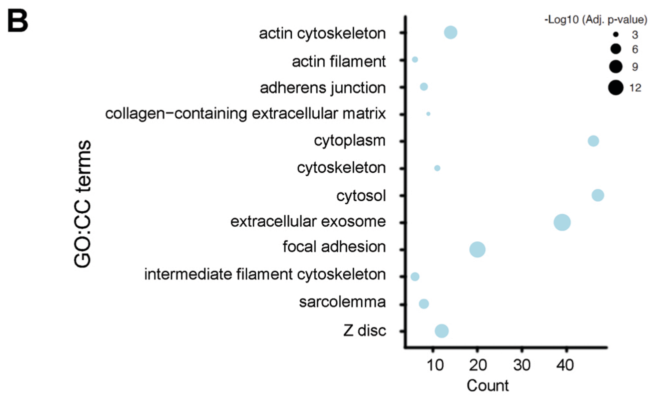
B
-Log10 (Adj. p-value)
3
6
9
12
actin cytoskeleton
actin filament
adherens junction
collagen−containing extracellular matrix
cytoplasm
cytoskeleton
cytosol
extracellular exosome
focal adhesion
intermediate filament cytoskeleton
sarcolemma
Z disc
GO:CC terms
10
20
30
40
Count

S_Figure 2. GO analysis of the TMT experiment.
Bubble plot showing the top 12 significantly enriched GO Cellular Component (GO:CC) terms derived from proteins whose expression levels were significantly increased (A) and decreased (B) by 10 mM NALL treatment.

S_Figure 3

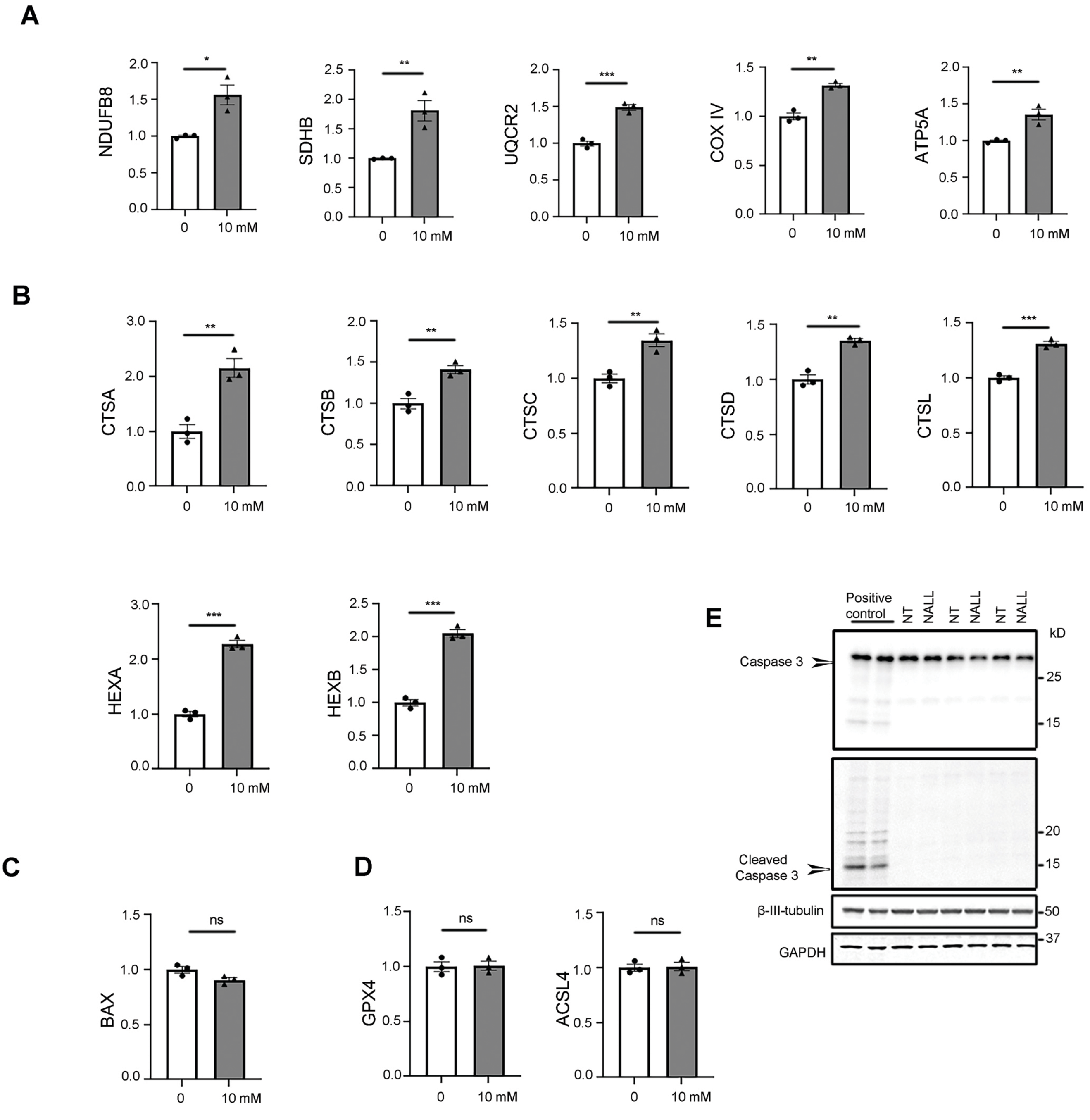

A
NDUFB8
SDHB
UQCR2
COX IV
ATP5A
0
10 mM
B
CTSA
CTSB
CTSC
CTSD
CTSL
HEXA
HEXB
C
BAX
ns
D
GPX4
ACSL4
E
Positive control
NT
NALL
kD
Caspase 3
Cleaved Caspase 3
β-III-tubulin
GAPDH
25
15
20
50
37

S_Figure 3. Selected mitochondrial and lysosomal protein levels from the TMT experiment. **(A)** Summary normalized quantification of mitochondrial electron transport chain (ETC) protein levels from the TMT-based proteomic analysis (n = 3 independent experiments). **(B)** Summary normalized quantification of mitochondrial lysosomal protein levels from the TMT-based proteomic analysis (n = 3 independent experiments). **(C)** Summary normalized quantification of BAX protein levels from the TMT-based proteomic analysis (n = 3 independent experiments). **(D)** Summary normalized quantification of GPX4 and ACSL4 protein levels from the TMT-based proteomic analysis (n = 3 independent experiments). **(E)** Representative Western blot showing no caspase-3 activation following NALL treatment.

p-value calculated using an unpaired t-test. ns, not significant, *$p < 0.05$, **$p < 0.01$, ***$p < 0.005$.

S_Figure 4

GBA1^{L444P}

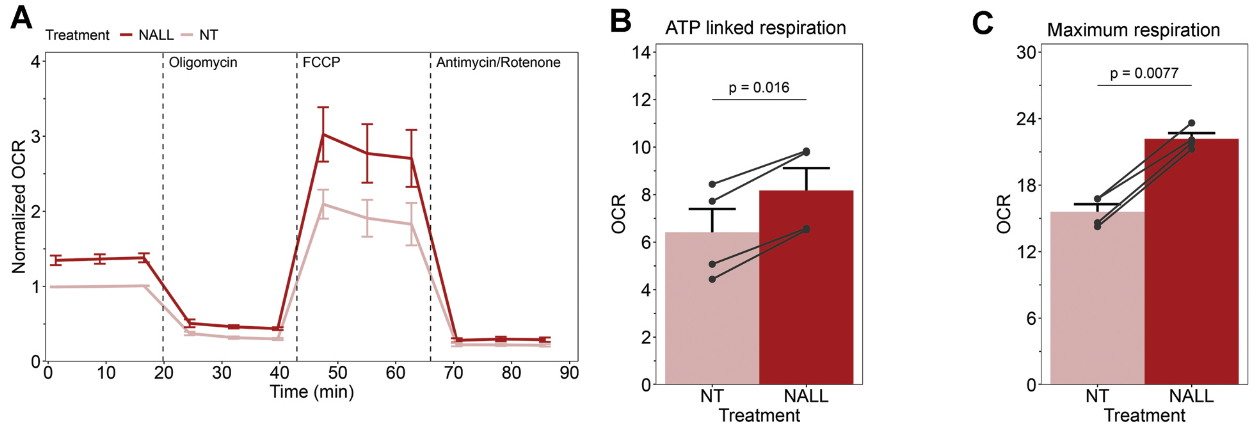

GBA1^{N370S}

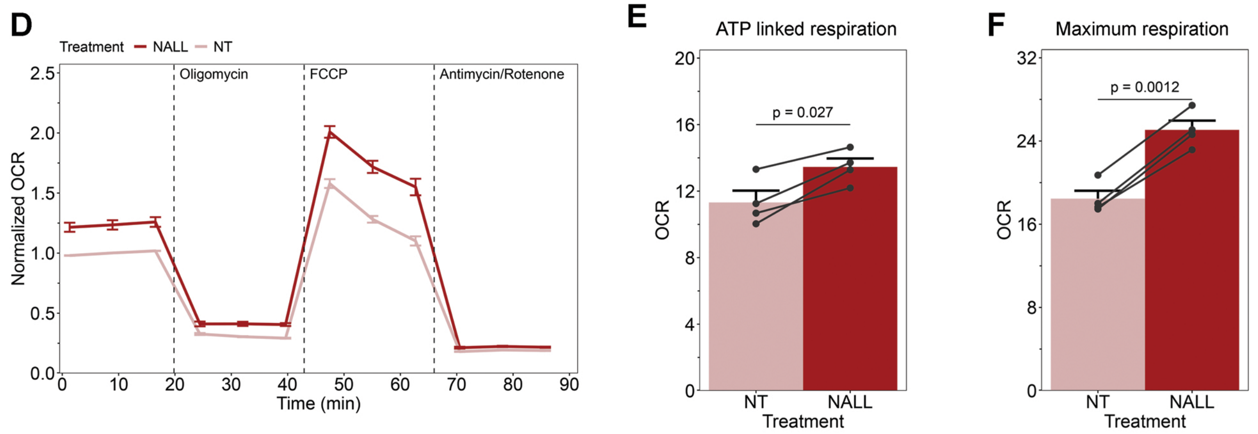

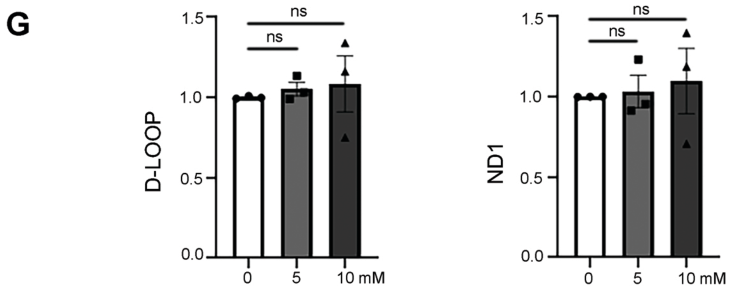

S_Figure 4. Increased mitochondrial function

(A) Seahorse respirometry trace showing oxygen consumption rate (OCR) of *GBA1* L444P mutant neurons, with or without 10 mM NALL treatment. N = 4 biological replicates. For each biological replicate, data were normalized to the mean basal OCR of the non-treated cells, as measured during the first three timepoints. Error bars = SEM.

(B) ATP-linked respiration of *GBA1* L444P mutant neurons, with or without 10 mM NALL treatment. N = 4 biological replicates. Oxygen consumption rate (OCR) was normalized to cell abundance as measured by total protein content, with units of pmol/min/mg. Error bars = SEM across 4 biological replicates. p-value calculated using paired t-test.

(C) Maximal respiration of *GBA1* L444P mutant neurons, with or without 10 mM NALL treatment. N = 4 biological replicates. Oxygen consumption rate (OCR) was normalized to cell abundance as measured by total protein content, with units of pmol/min/mg. Error bars = SEM across 4 biological replicates. p-value calculated using paired t-test.

(D) Seahorse respirometry trace showing oxygen consumption rate (OCR) of *GBA1* N370S mutant neurons, with or without 10 mM NALL treatment. N = 4 biological replicates. For each biological replicate, data were normalized to the mean basal OCR of the non-treated cells, as measured during the first three timepoints. Error bars = SEM.

(E) ATP-linked respiration of *GBA1* N370S mutant neurons, with or without 10 mM NALL treatment. N = 4 biological replicates. Oxygen consumption rate (OCR) was normalized to cell abundance as measured by total protein content, with units of pmol/min/mg. Error bars = SEM across 4 biological replicates. p-value calculated using paired t-test.

(F) Maximal respiration of *GBA1* N370S mutant neurons, with or without 10 mM NALL treatment. N = 4 biological replicates. Oxygen consumption rate (OCR) was normalized to cell abundance as measured by total protein content, with units of pmol/min/mg. Error bars = SEM across 4 biological replicates. p-value calculated using paired t-test.

(**G**) qPCR analysis showed no significant change of the mitochondrial DNA D-LOOP (left) and ND1 (right) upon NALL treatment (n = 3 independent experiments; One-way ANOVA). Data are shown as mean ± SEM; *ns*, not significant.

S_Figure 5

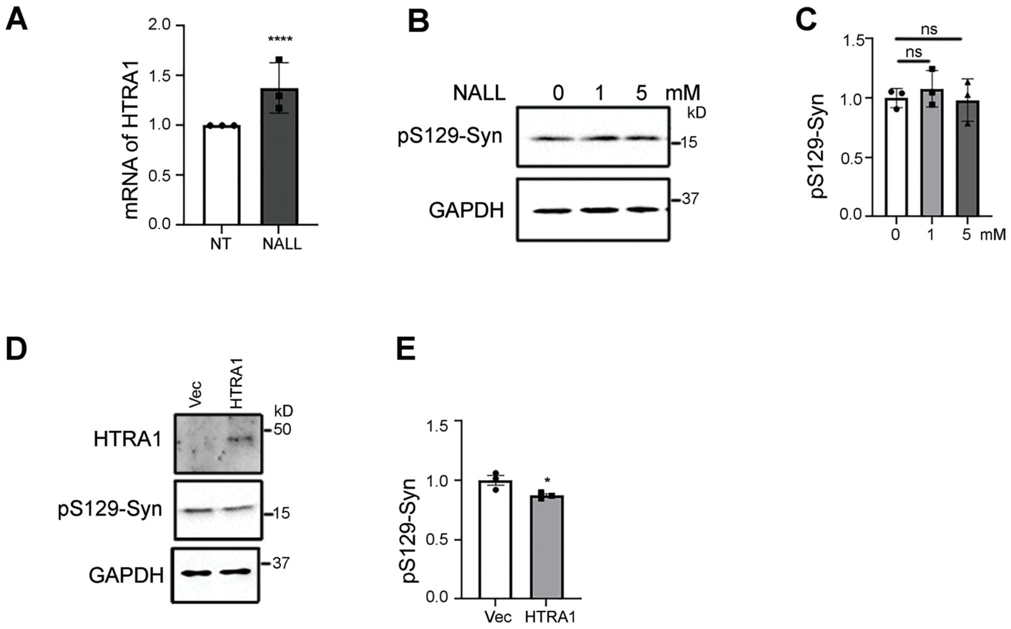

A
mRNA of HTRA1
2.0
1.5
1.0
0.5
0.0
****
NT
NALL
B
NALL
0
1
5
mM
kD
pS129-Syn
15
GAPDH
37
C
ns
ns
1.5
1.0
0.5
0.0
pS129-Syn
0
1
5
mM
D
Vec
HTRA1
kD
HTRA1
50
pS129-Syn
15
GAPDH
37
E
1.5
1.0
0.5
0.0
pS129-Syn
*
Vec
HTRA1

S_Figure 5. pS129-syn didn't decrease upon NALL treatment in α-syn expressed SH-SY5Y cells.
(A) qPCR analysis showed increased mRNA level of HTRA1 following NALL treatment in *GBA1* L444P neurons (n = 3 independent experiments; t-test).
(B) Representative Western blot showing pS129-syn levels in α-syn–stably expressing SH-SY5Y cells treated with increasing concentrations of NALL for 7 days. GAPDH served as a loading control.
(C) Quantification of pS129-syn levels from (B), presented as the average pS129-syn signal normalized to GAPDH and expressed relative to the 0 mM (DMSO) group (n = 3 independent experiments; One-way ANOVA).
(D) Representative Western blot showing HTRA1 and pS129-syn levels in α-syn–stably expressing SH-SY5Y cells transfected with HTRA1 or empty vector (Vec). GAPDH served as a loading control.

(E) Quantification of pS129-syn levels from (B), presented as the average pS129-syn signal normalized to GAPDH and expressed relative to Vector (n = 3 independent experiments; t-test). All data are shown as mean ± SEM; ns, not significant. ns, not significant, *p < 0.05, ****p < 0.001.

Figure S6

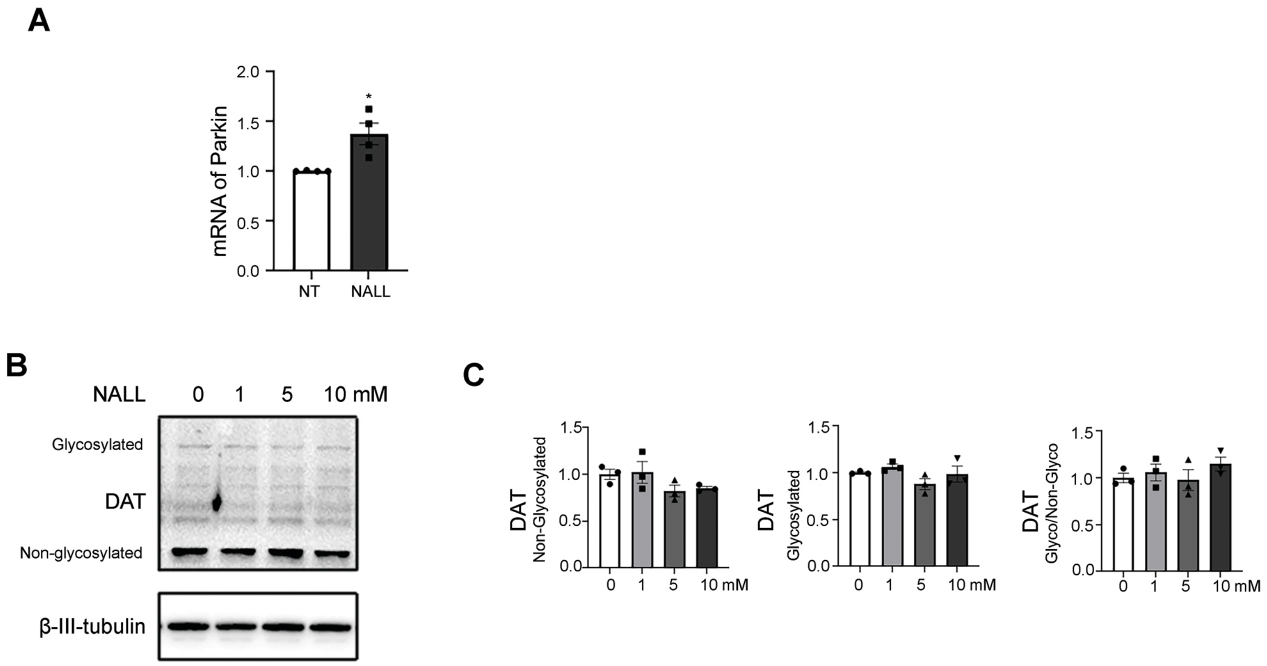

A
mRNA of Parkin
2.0
1.5
1.0
0.5
0.0
*
NT
NALL
B
NALL 0 1 5 10 mM
Glycosylated
DAT
Non-glycosylated
β-III-tubulin
C
DAT
Non-Glycosylated
DAT
Glycosylated
DAT
Glyco/Non-Glyco
0 1 5 10 mM

S_Figure 6. There was no significant change in DAT upon NALL treatment in parkin mutant dopaminergic neurons

(A) qPCR analysis showed increased parkin mRNA level following NALL treatment in *GBA1* L444P neurons (n = 3 independent experiments; t-test).

(B) Western blot of DAT upon different concentrations of NALL treatment in *parkin* mutant neurons. β-III-Tubulin was used as a loading control.

(C) Quantification of the fold change of DAT upon NALL treatment in A. The data is presented as the average DAT signal (normalized to β-III-Tubulin) relative to the 0 mM (DMSO only) group (n = 3 independent experiments; One-way ANOVA). All data are represented as mean ± SEM, ns: not significant. ns, not significant, *$p < 0.05$.

S_Figure 7

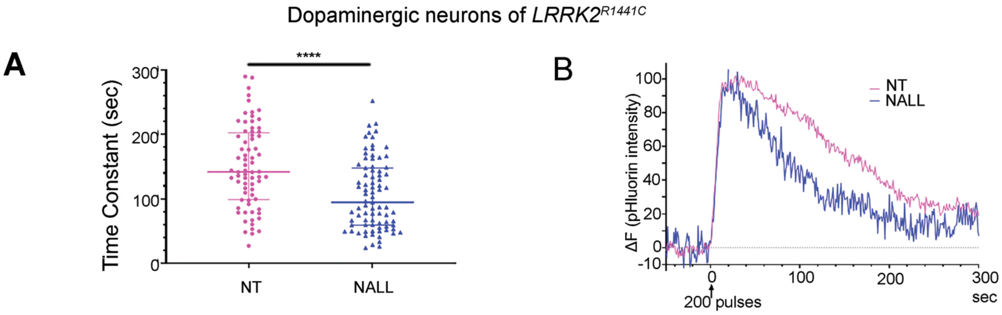

Dopaminergic neurons of *LRRK2*^{R1441C}
A
****
Time Constant (sec)
300
200
100
0
NT
NALL
B
NT
NALL
ΔF (pHluorin intensity)
100
80
60
40
20
0
-10
0
100
200
300
sec
200 pulses

S_Figure 7. NALL increases synaptic endocytosis in LRRK2 mutant neurons.

(A) Scatter plot showing time constants of pHluorin fluorescence recovery following exocytosis in *LRRK2* R1441C mutant neurons. NALL-treated synapses (105.3 ± 5.8 s; n = 59 ROIs) recovered significantly faster than NT (133.2 ± 5.3 s; n = 89 ROIs) (Mann–Whitney test). Lines represent means ± SD; ****$p < 0.001$.
(B) Representative traces of pHluorin fluorescence intensity from 50 s before to 300 s after exocytosis in *LRRK2* R1441C mutant neurons (n = 59 ROIs).

S_Figure 8

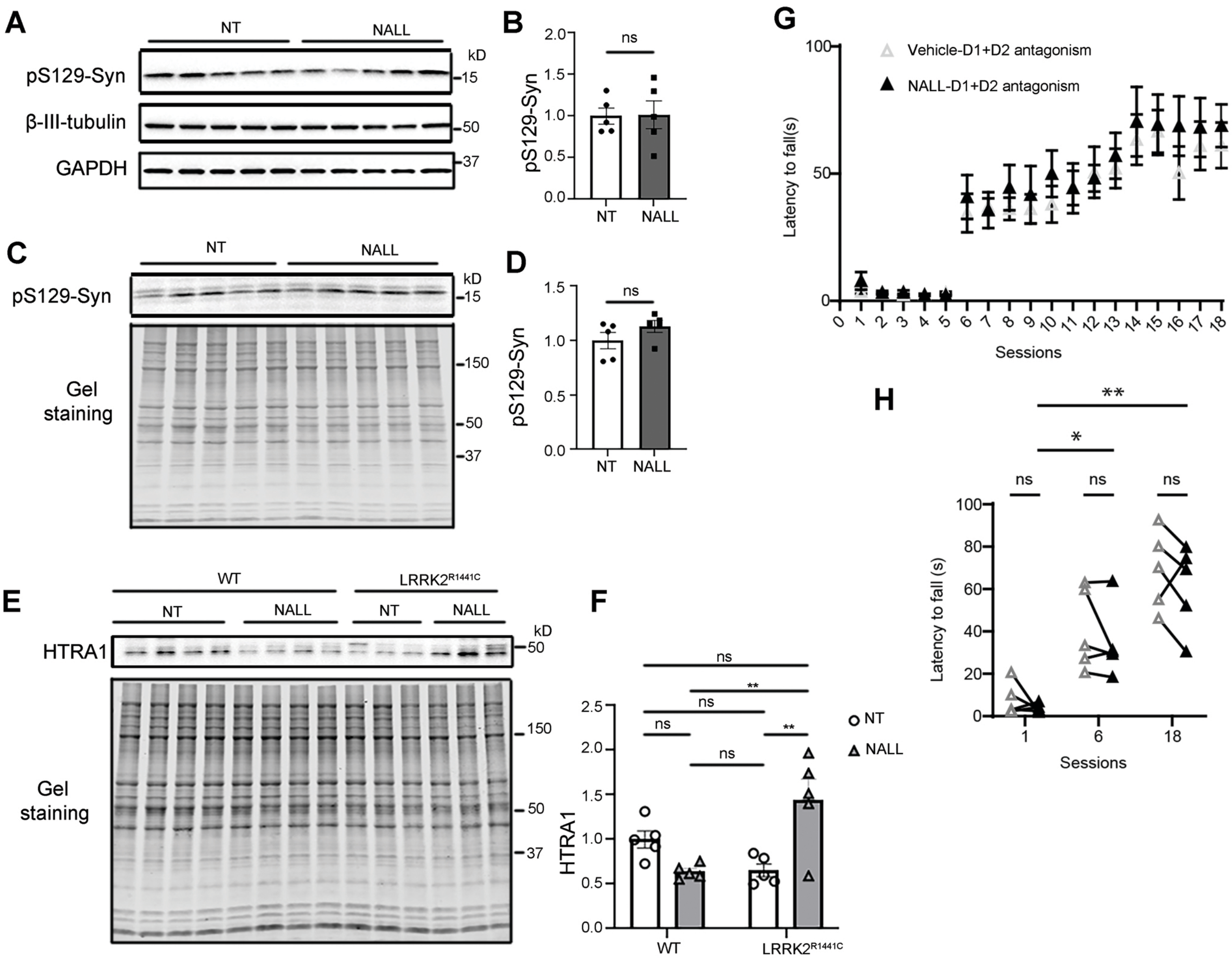

A
NT
NALL
kD
pS129-Syn
β-III-tubulin
GAPDH
15
50
37
B
pS129-Syn
ns
NT
NALL
C
NT
NALL
kD
pS129-Syn
15
Gel staining
150
50
37
D
pS129-Syn
ns
NT
NALL
E
WT
LRRK2R1441C
NT
NALL
NT
NALL
kD
HTRA1
50
Gel staining
150
50
37
F
HTRA1
ns
**
ns
ns
**
ns
NT
NALL
WT
LRRK2R1441C
G
Vehicle-D1+D2 antagonism
NALL-D1+D2 antagonism
Latency to fall(s)
Sessions
H
**
*
ns
ns
ns
Latency to fall (s)
Sessions

S_Figure 8. NALL does not alter pS129-syn or motor learning in LRRK2^{WT} mice.

(A) Representative Western blots showing pS129-syn in the Triton-soluble fraction of the substantia nigra from *LRRK2* ^{WT} mice treated with NALL (NALL) or vehicle (NT). β-III-Tubulin and GAPDH were used as loading controls.

(B) Quantification of fold changes in pS129-syn following NALL treatment shown in (A). Data represent average pS129-syn levels (normalized to β-III-Tubulin), expressed relative to the NT (vehicle-only) group (*n* = 5 mice; t-test).

(C) Western blots showing pS129-syn in the Triton-insoluble fraction of *LRRK2*^{WT} mouse substantia nigra with or without NALL treatment. Total protein staining was used as a loading control.

(D) Quantification of fold changes in pS129-syn from (C). Data represent average pS129-syn normalized to total protein levels relative to the NT (vehicle-only) group (*n* = 5 mice; t-test).

(E) Western blots showing HTRA1 in the Triton-insoluble fraction of *LRRK2*^{WT} and *LRRK2*^{R1441C} mouse substantia nigra with or without NALL treatment. Total protein staining was used as a loading control.

(F) Quantification of fold changes in HTRA1. Data represent average HTRA1 normalized to total protein levels relative to the NT (vehicle-only) group (*n* = 5 mice for each group; Two-way ANOVA).

(G) There was no difference in the performance of *LRRK2*^{WT} mice that received vehicle or NALL (treatment p=0.5551, session p<0.0001, treatment x session Factor p=0.7524, Subject p<0.0001) in the same rotarod paradigm as in Figure 6.

(H) Summary of average latency to fall in sessions 1, 6, and 18 from panel G. Data are shown as mean ± SEM. ns indicates: no significance; asterisks denote statistical significance based on Tukey's multiple-comparisons test following 2-way repeated-measures ANOVA (*p < 0.05, **p < 0.01). *n* = 5 mice per treatment group. ns, not significant, *p < 0.05, **p < 0.01.

Supplementary Table Legend

Table S1: The table summarizes all the TMT-MS data and gene annotation analysis.

Sheet 1: TMT reporter intensities for each quantified protein ID across all samples, including statistical analyses.

Sheet 2: Gene annotation analysis results for proteins significantly upregulated by 10 mM NALL treatment.

Sheet 3: Gene annotation analysis results for proteins significantly downregulated by 10 mM NALL treatment.

Table S2: The table summarizes lysosomal, synaptic, and mitochondrial proteins significantly upregulated (ratio NALL/NT > 1.5 and adjusted *p*-value < 0.05) by 10 mM NALL treatment.
