# Supplementary material for: N-acetyl-l-leucine lowers α-synuclein levels and improves synaptic function in Parkinson’s disease models
Source: J Clin Invest. 2026 Mar 2;136(5):e196137. doi: 10.1172/JCI196137 (PMC12948429; doi:10.1172/JCI196137)

Full unedited blot

Fig.1 A. pS129-syn

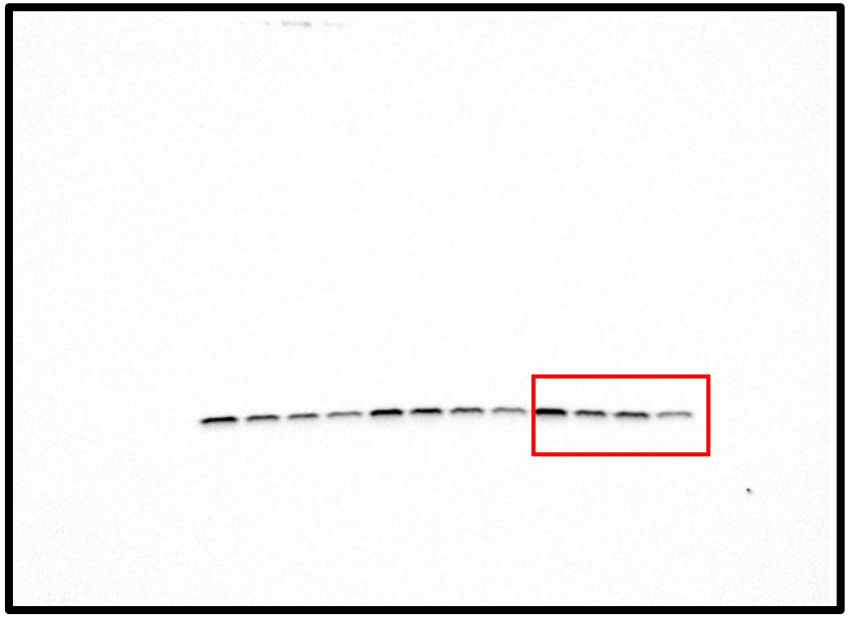

Merge with the marker

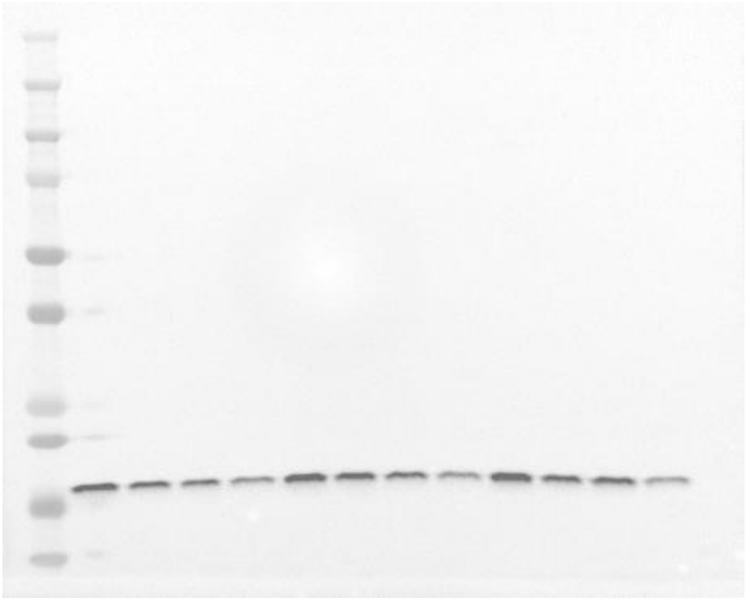

Fig.1 A. Syn

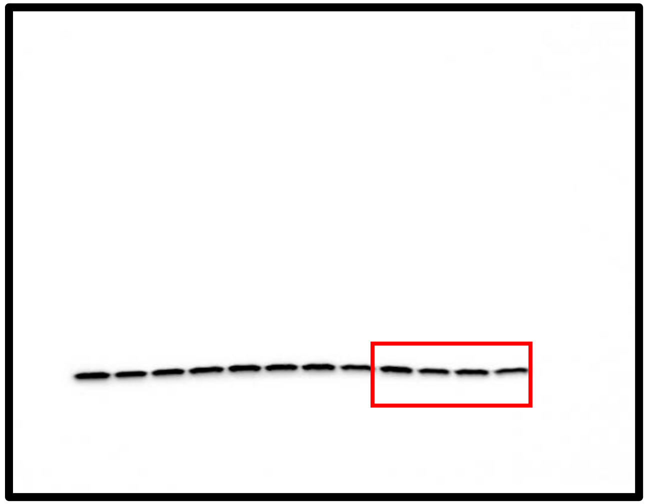

Merge with the marker

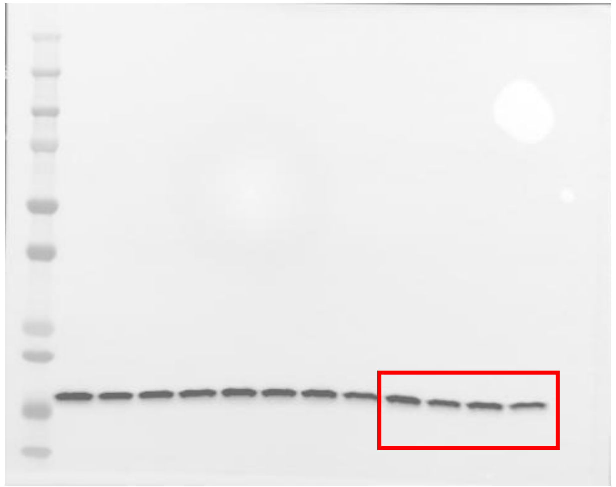

Fig.1 A. β-III-tubulin

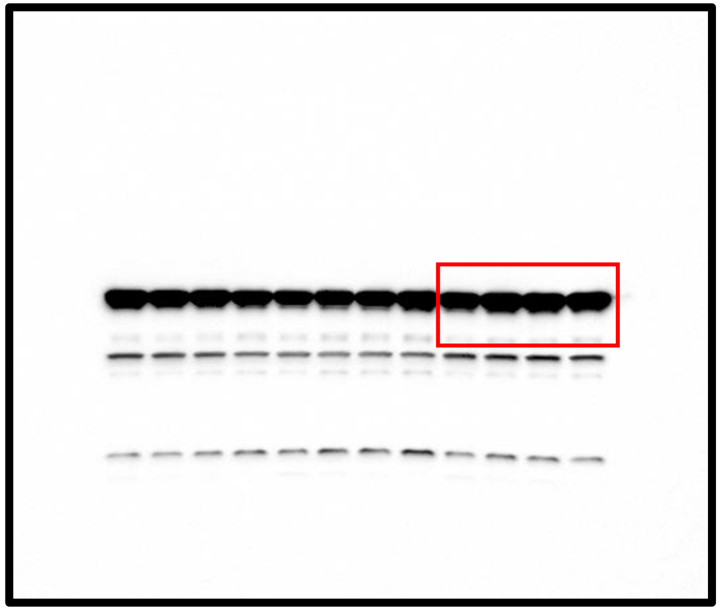

Merge with the marker

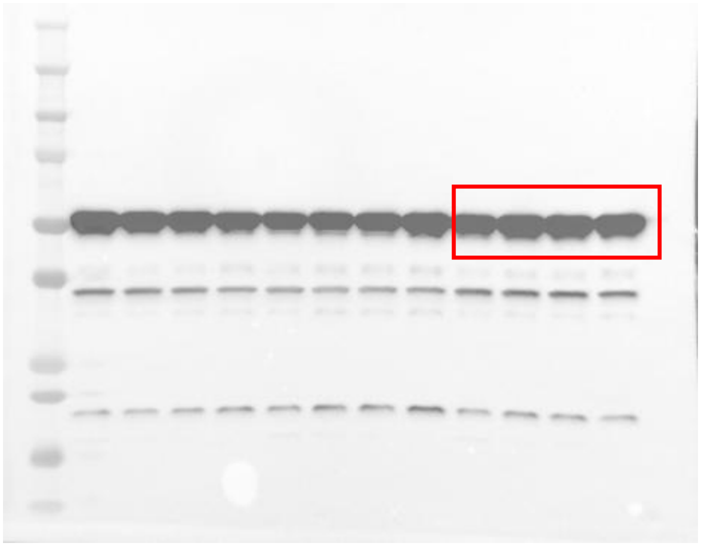

Fig.1 A. GAPDH

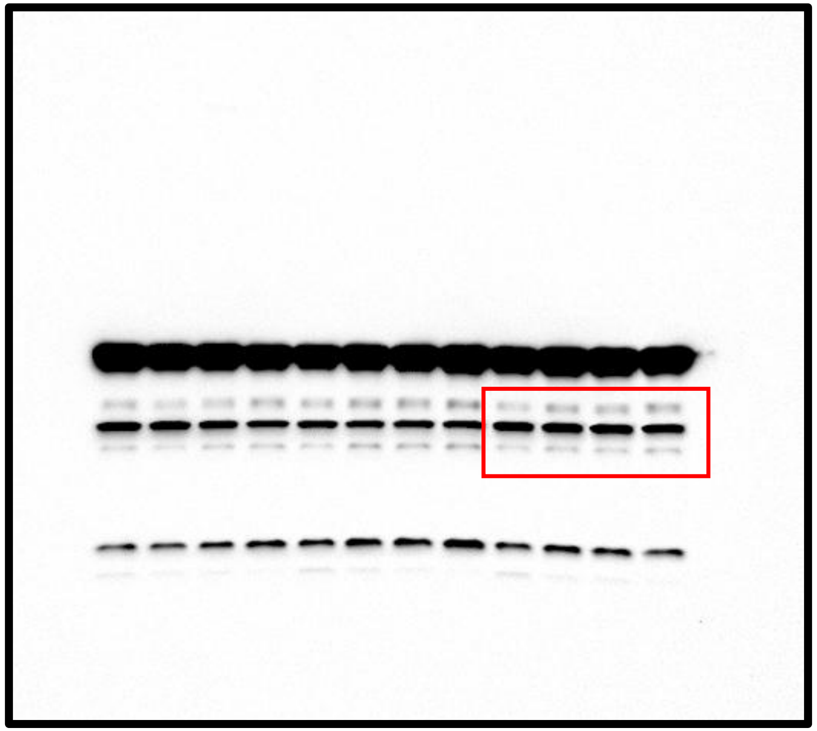

Merge with the marker

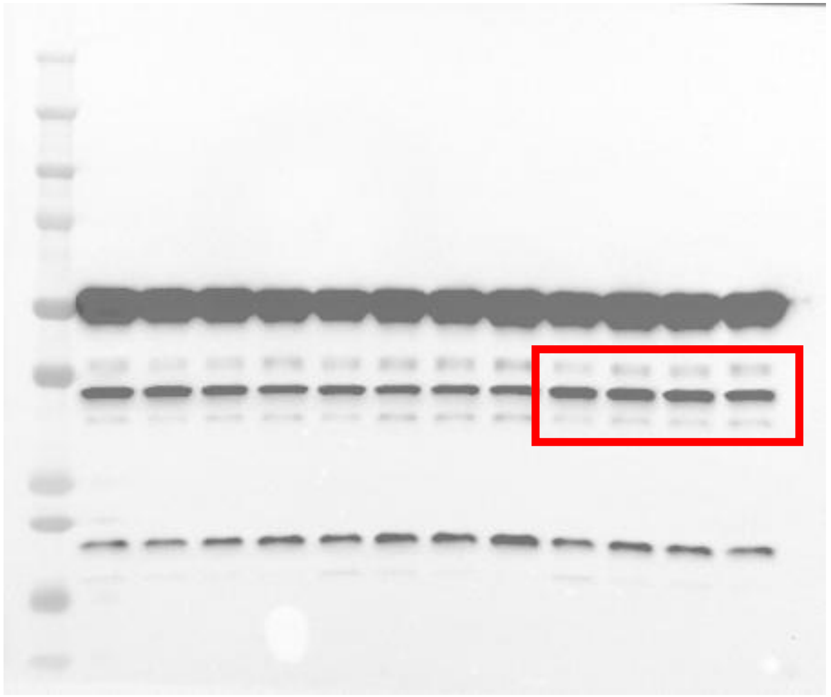

Fig.1 C. pS129-syn

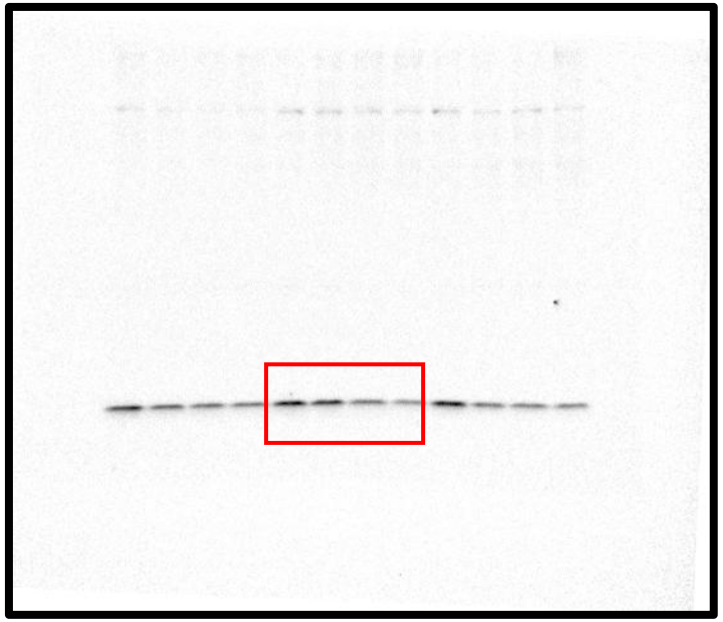

Merge with the marker

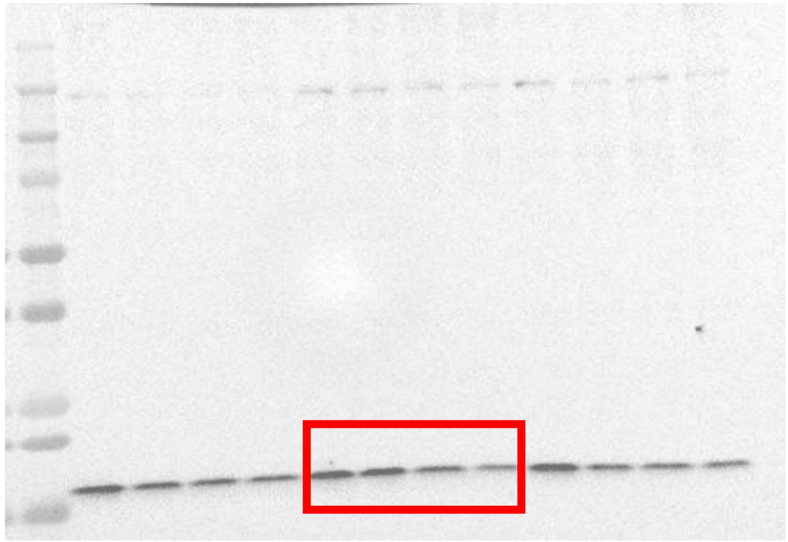

Fig.1 C. Syn

Merge with the marker

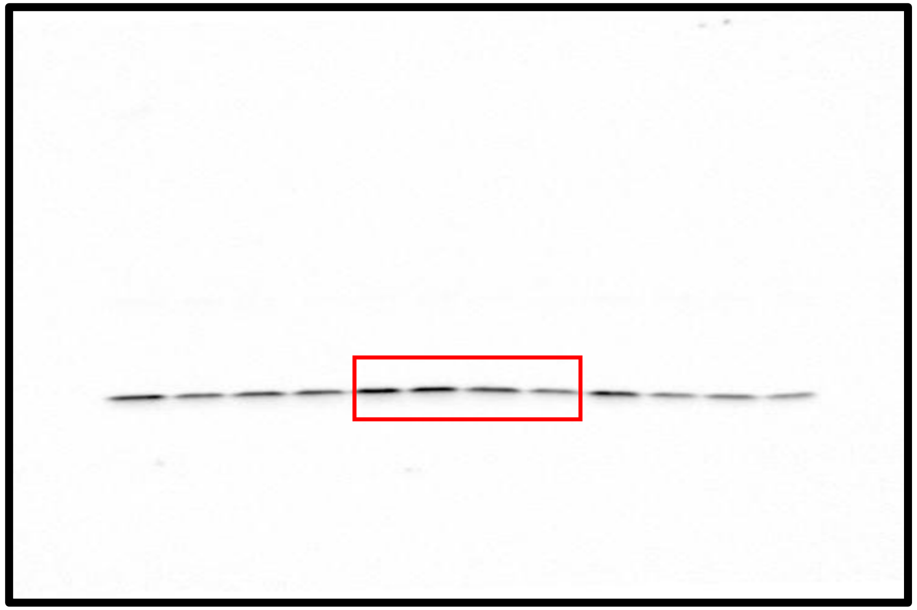

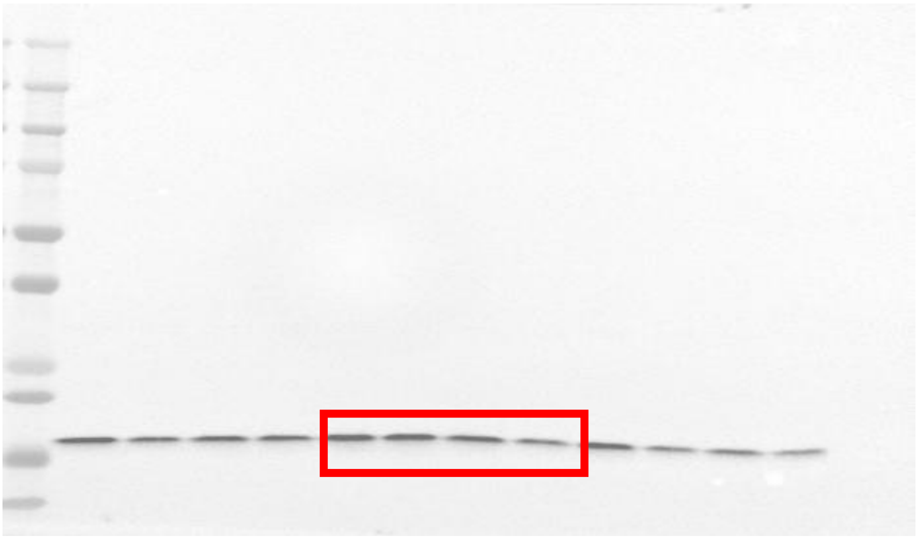

Fig.1 C. Gel staining

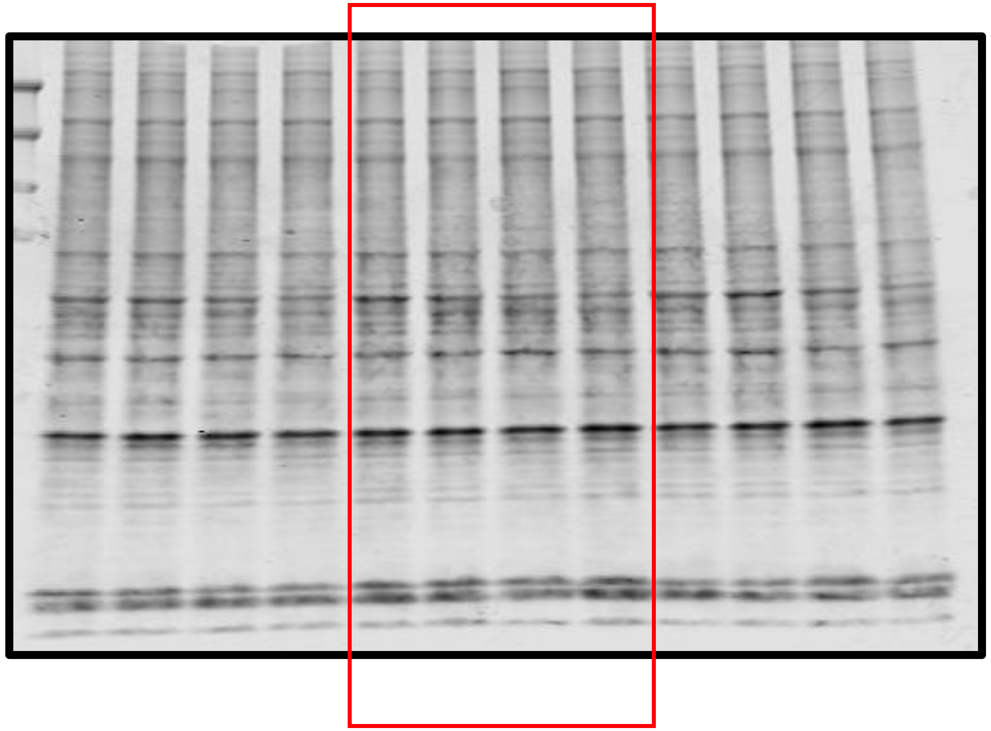

Fig.1 E. pS129-syn

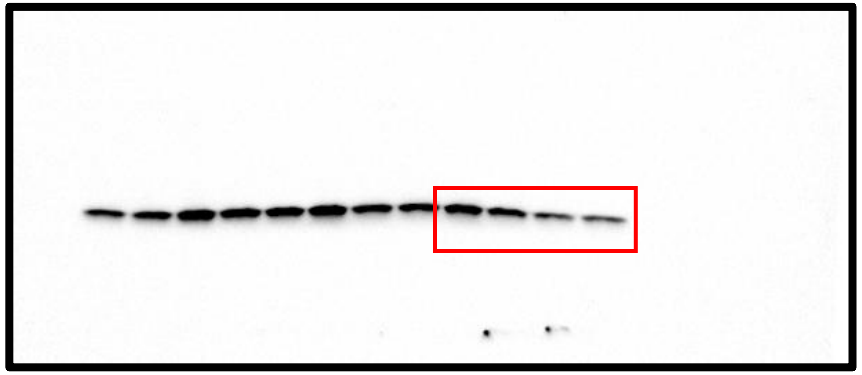

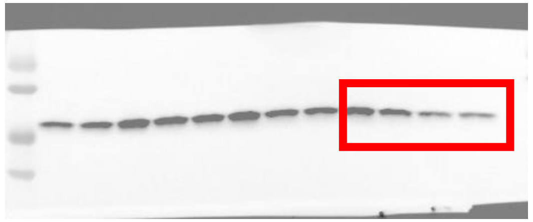

The membrane had been cut above 25kD before imaging

Fig.1 E. Syn

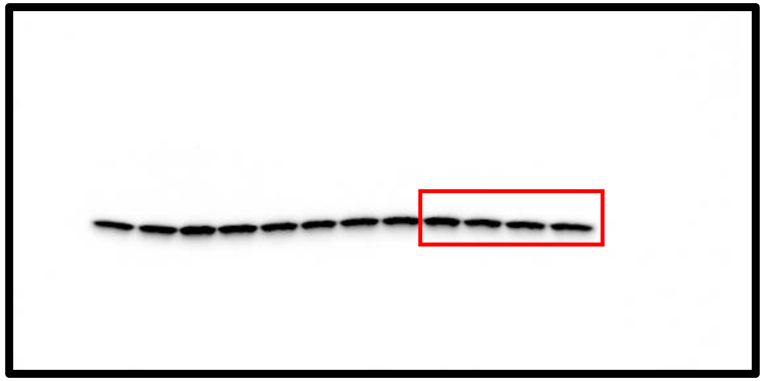

Merge with the marker

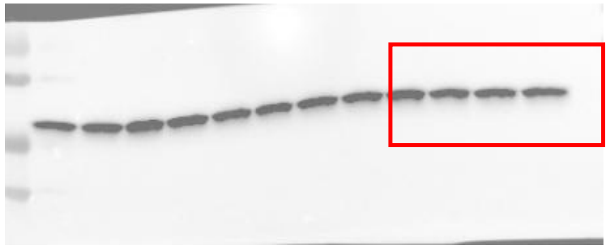

The membrane had been cut above 25kD before imaging

Fig.1 E. β-III-tubulin

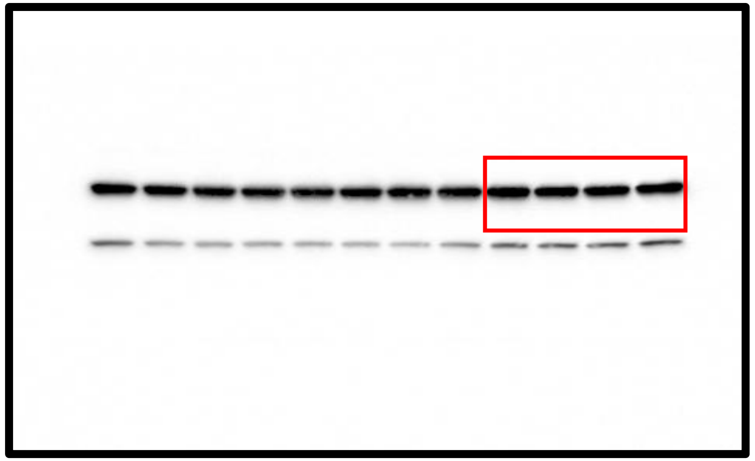

Merge with the marker

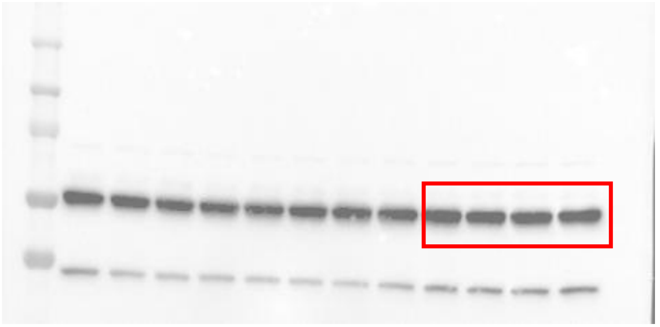

The membrane had been cut below 37kD before imaging

Fig.1 E. GAPDH

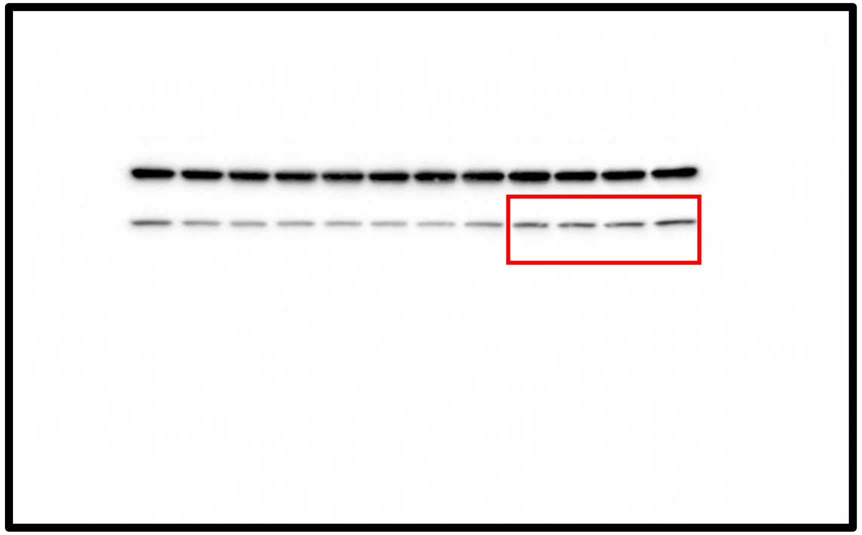

Merge with the marker

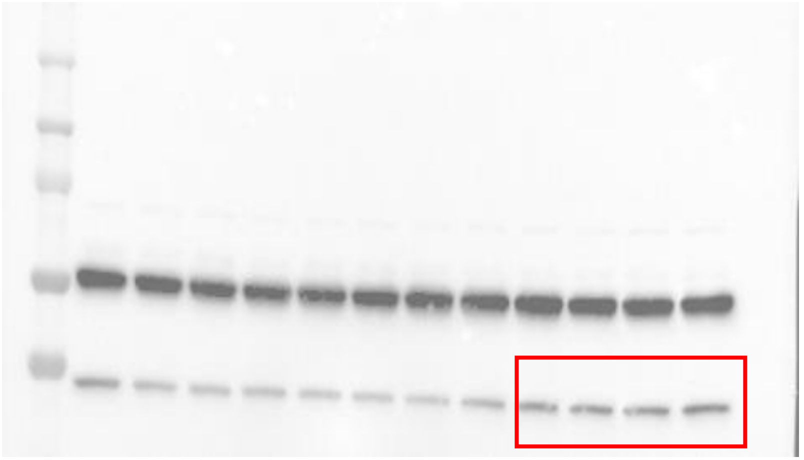

The membrane had been cut below 37kD before imaging

Fig.1 G. pS129-syn

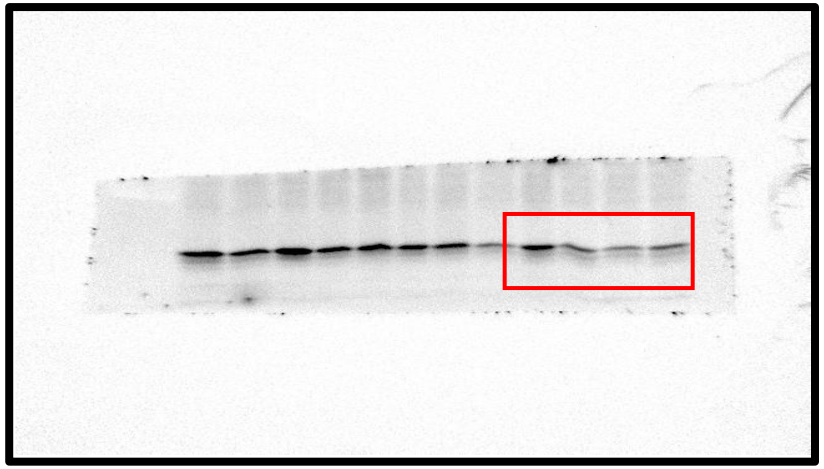

Merge with the marker

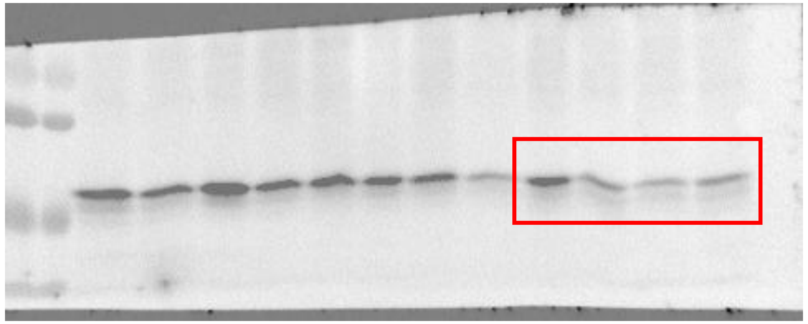

The membrane had been cut above 25kD before imaging

Fig.1 G. Syn

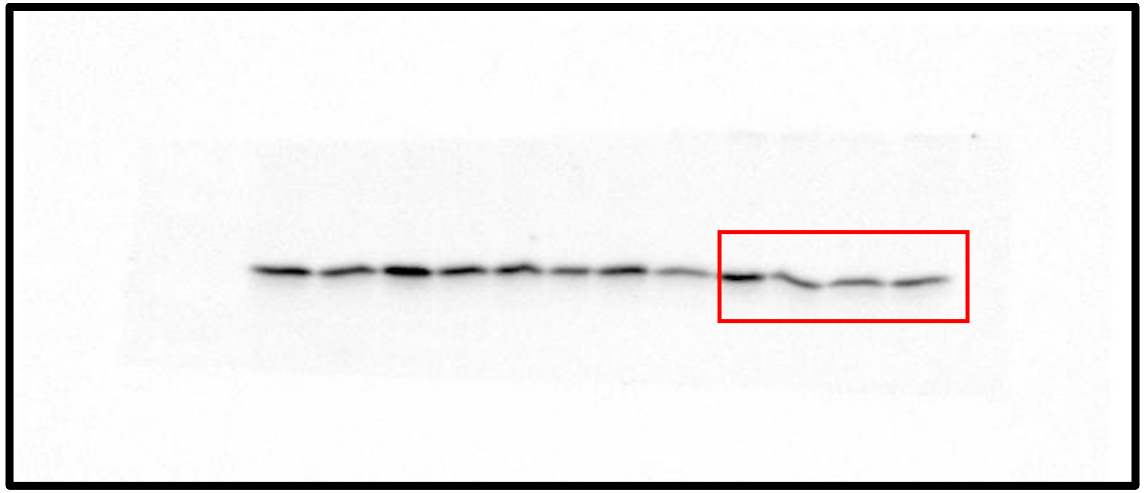

Merge with the marker

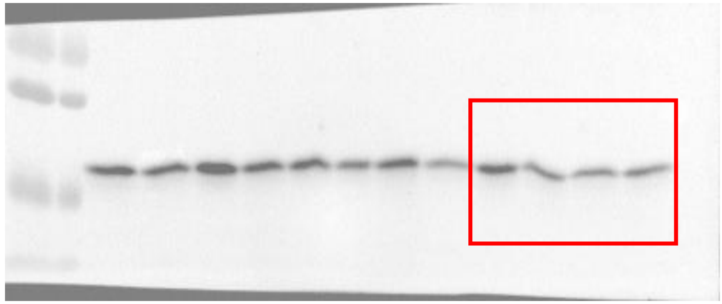

The membrane had been cut above 25kD before imaging

Fig.1 G. Gel staining

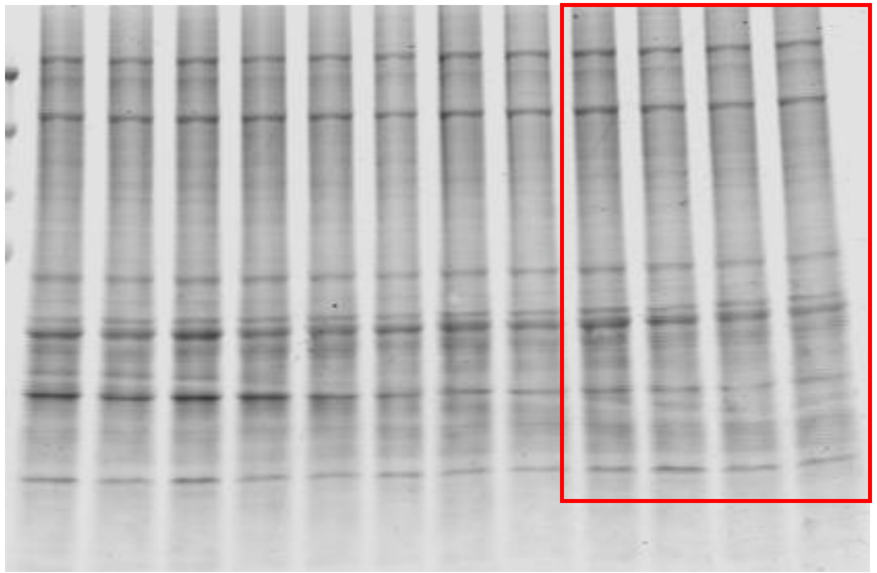

Fig.1 I. pS129-syn

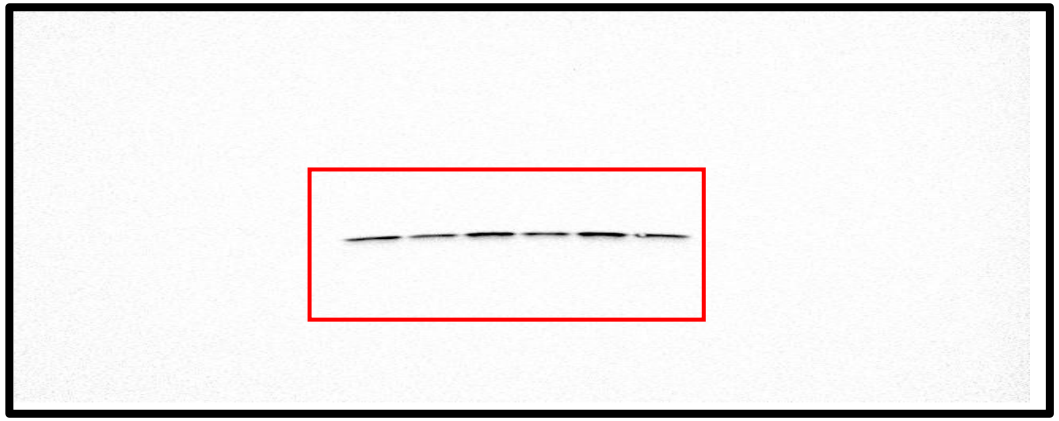

Merge with the marker

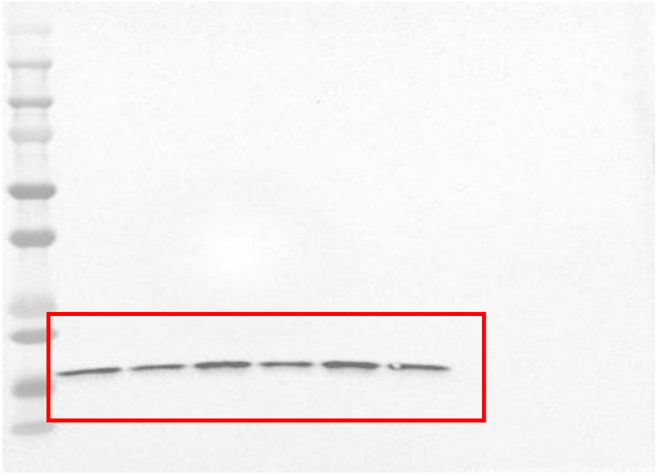

Fig.1 I. β-III-tubulin

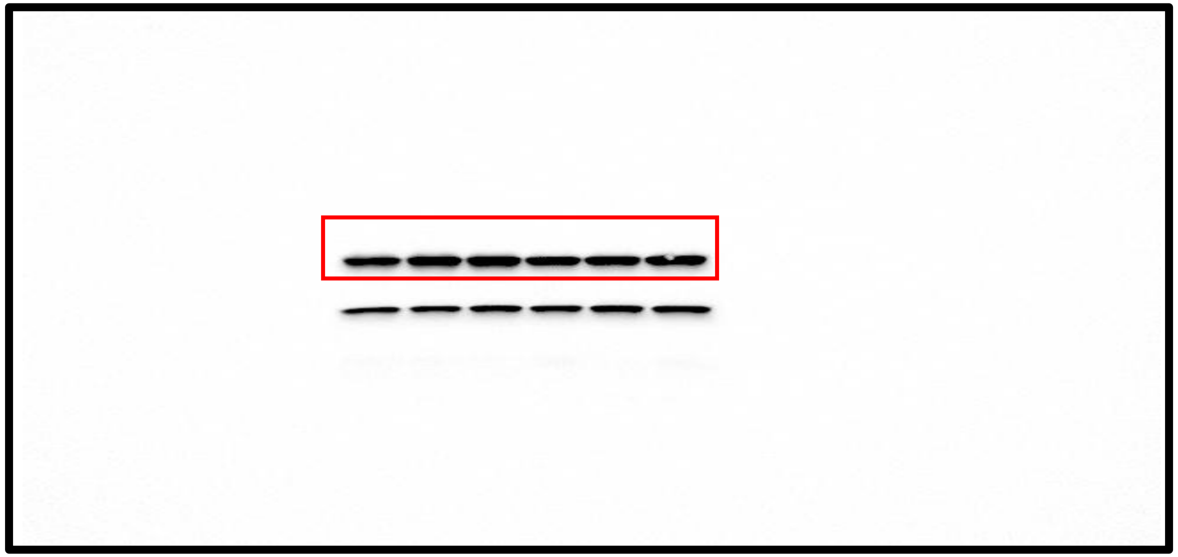

Merge with the marker

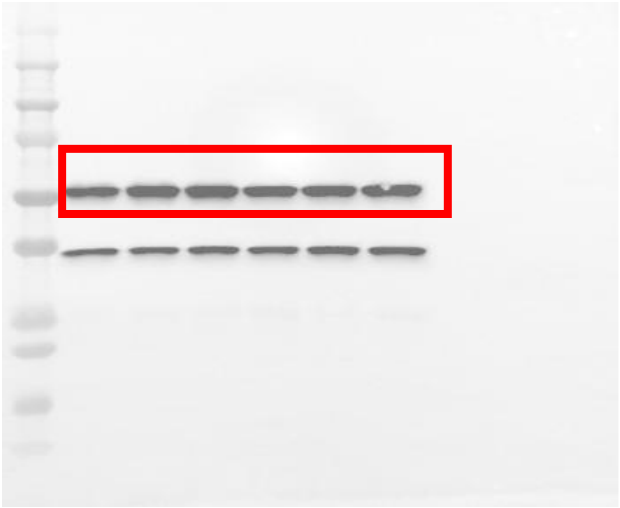

Fig.1 I. GAPDH

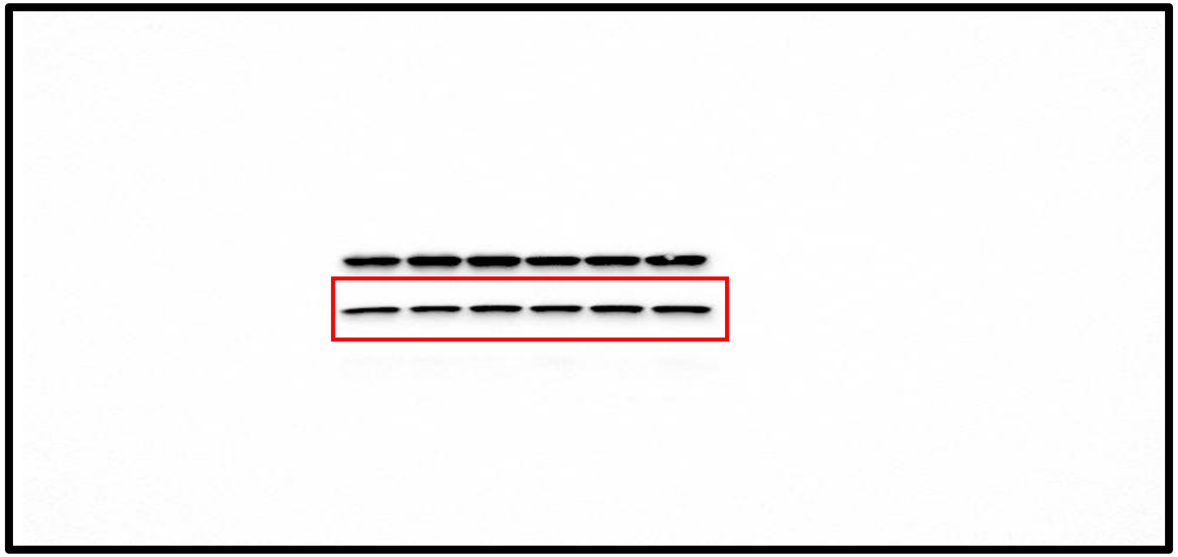

Merge with the marker

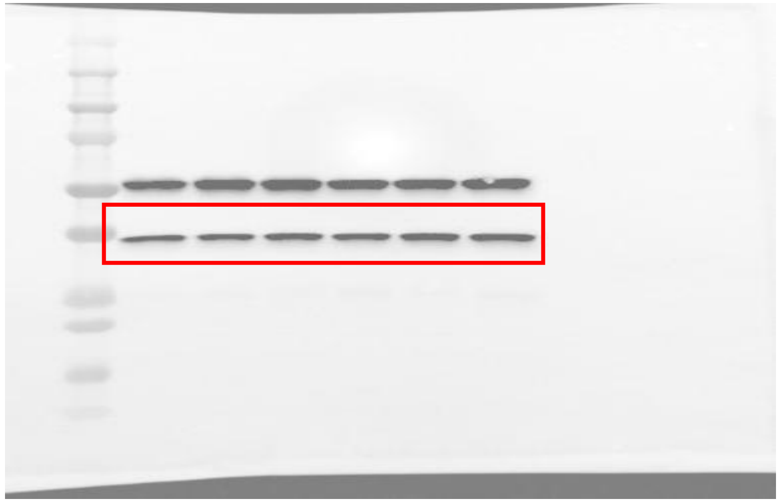

Fig.1 K. pS129-syn

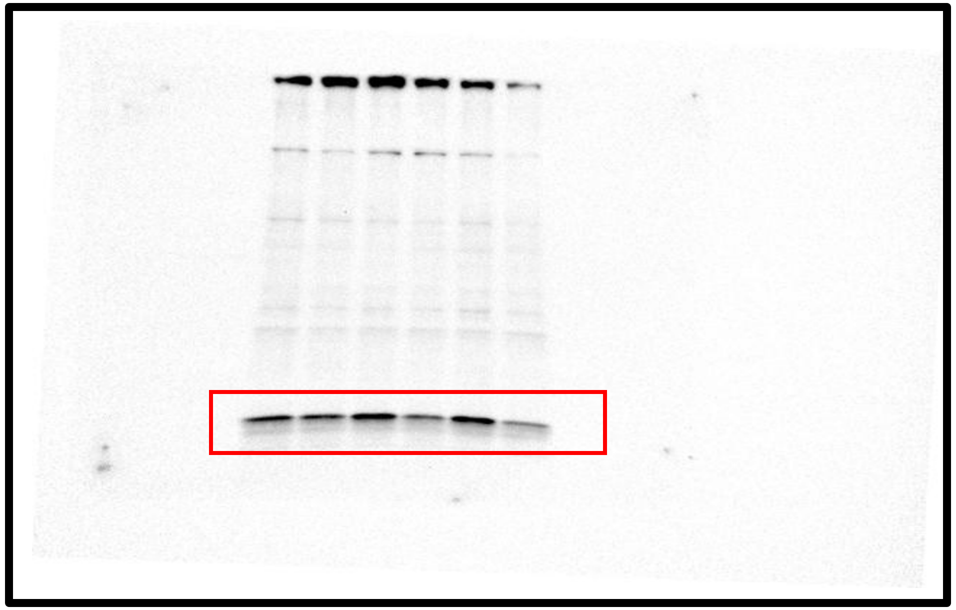

Merge with the marker

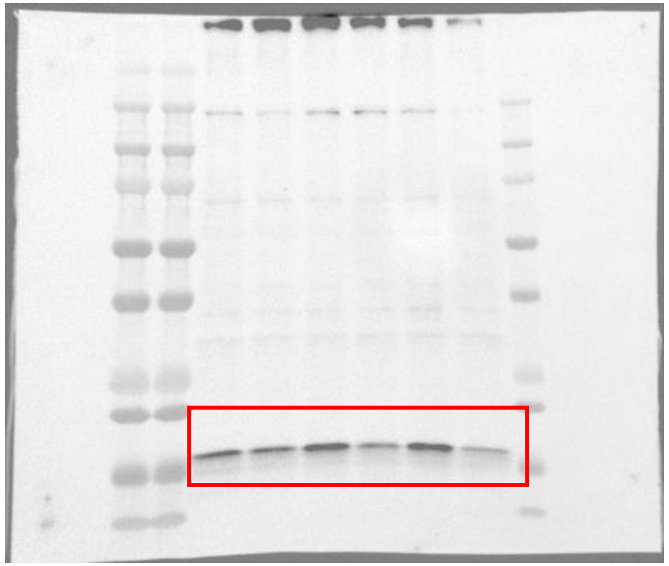

Fig.1 K. Gel staining

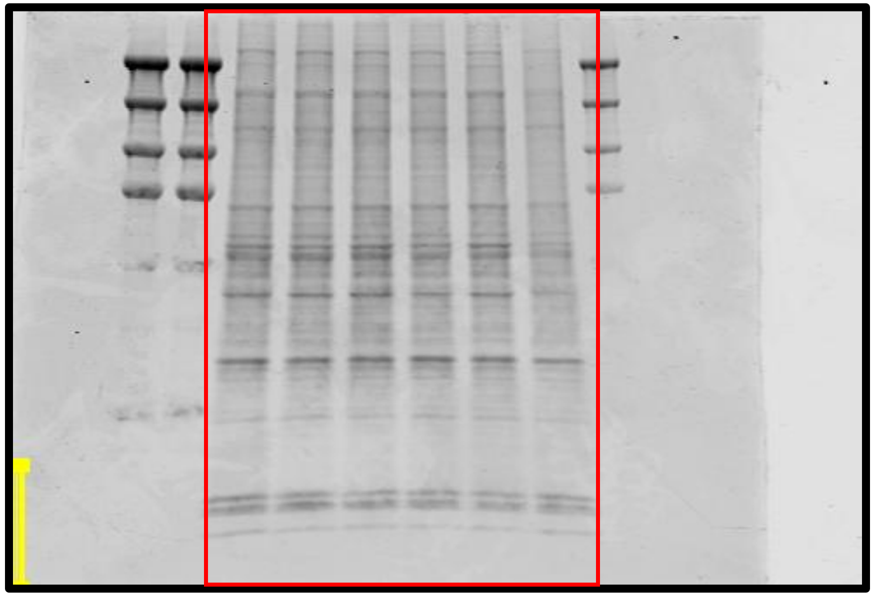

Fig.2 C. HTRA1

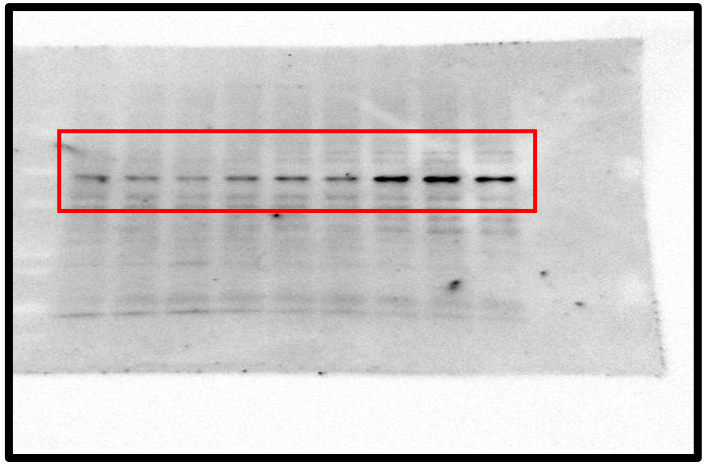

Merge with the marker

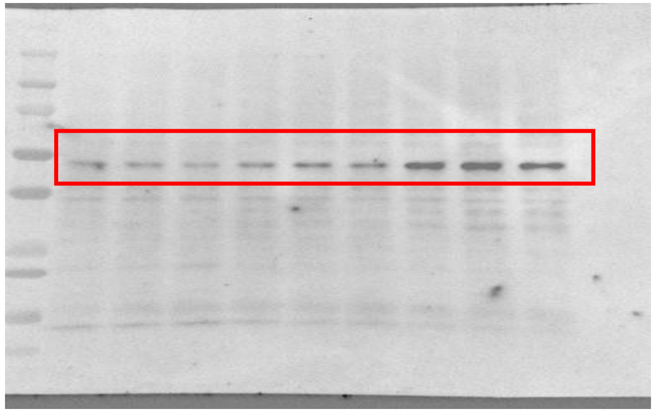

Fig.2 C. pS129-syn

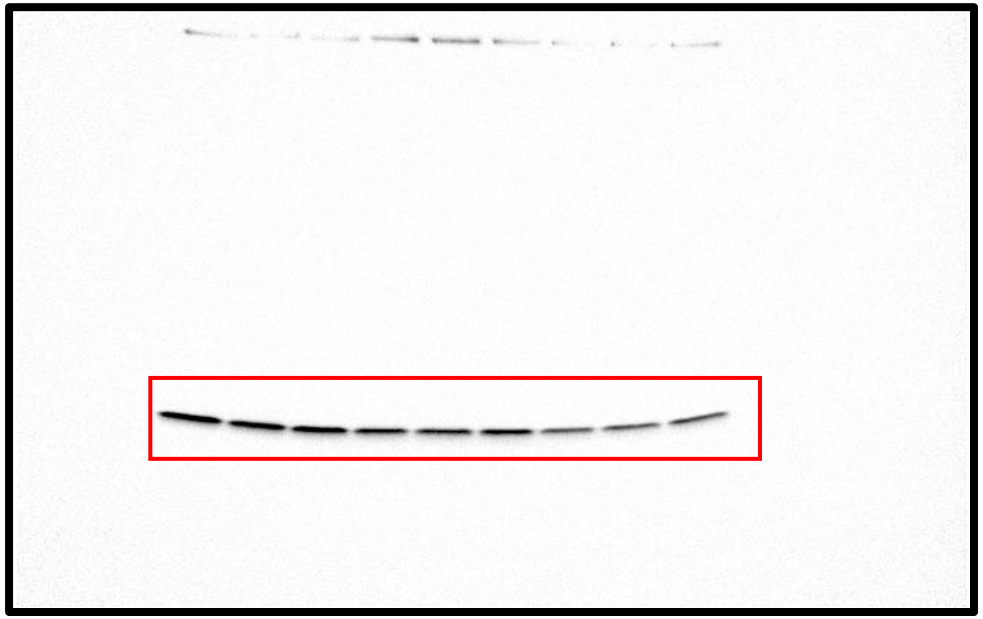

Merge with the marker

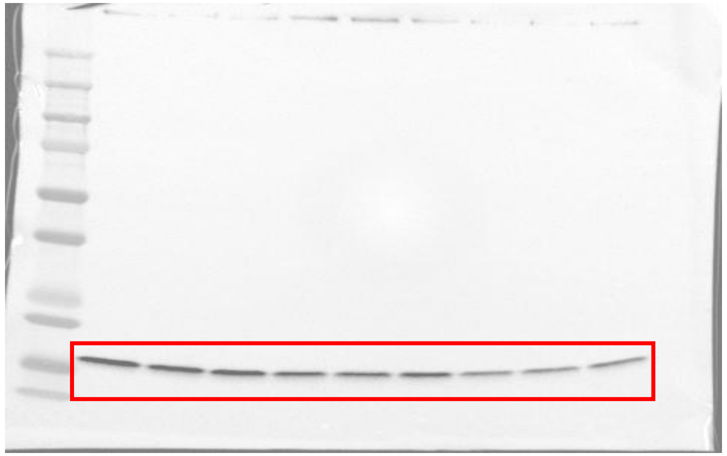

Fig.2 C. β-III-tubulin

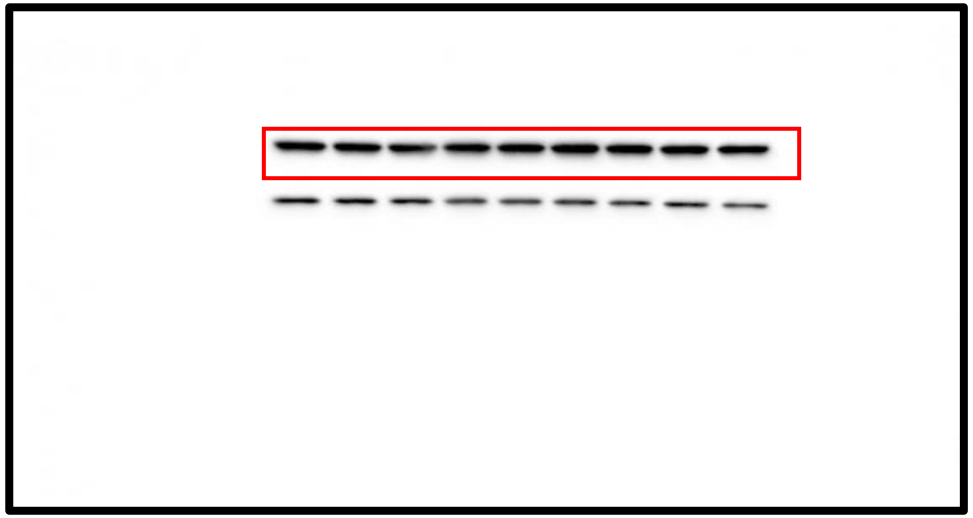

Merge with the marker

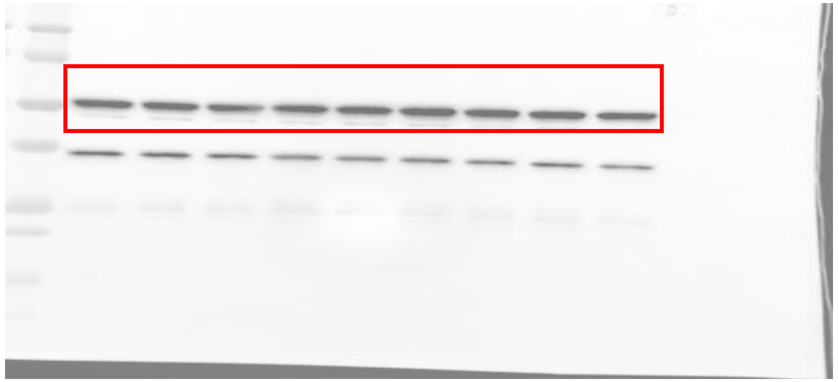

Fig.2 C. GAPDH

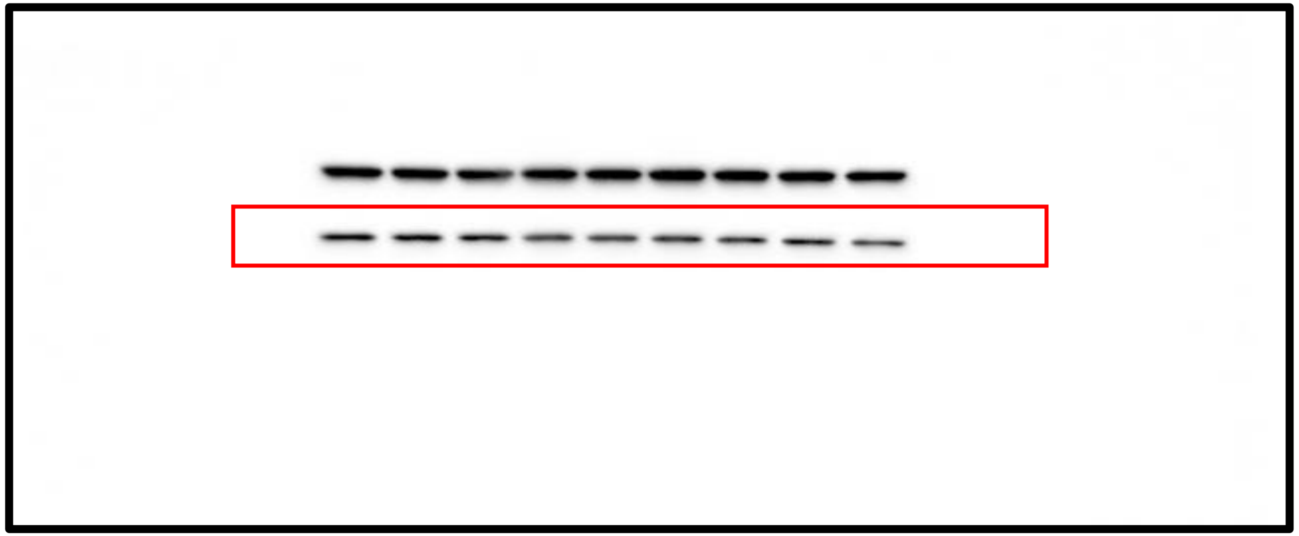

Merge with the marker

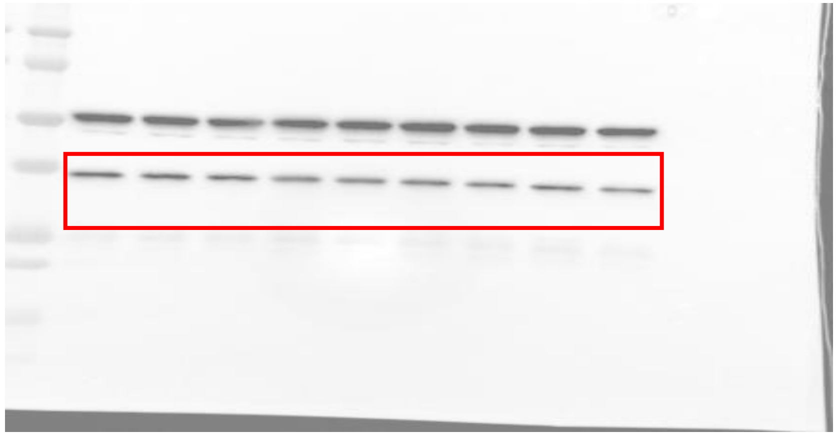

Fig.2 E. HTRA1

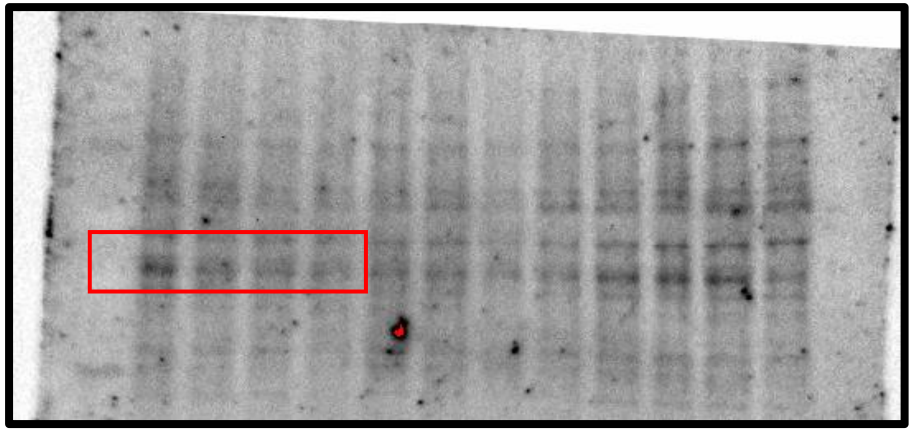

Merge with the marker

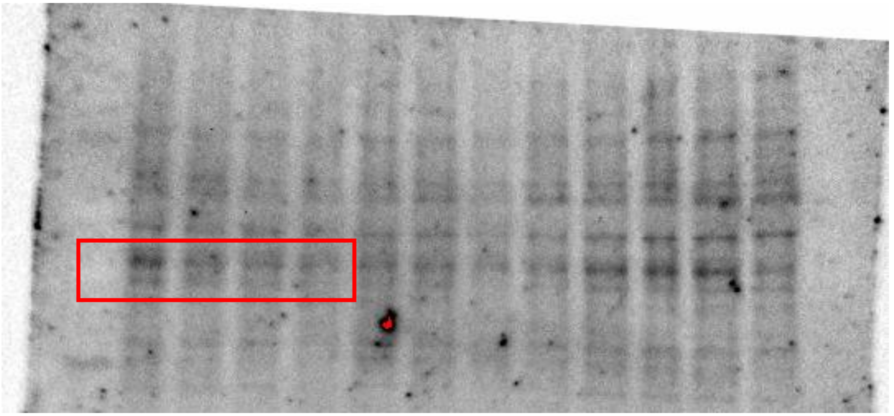

Fig.2 E. β-III-tubulin

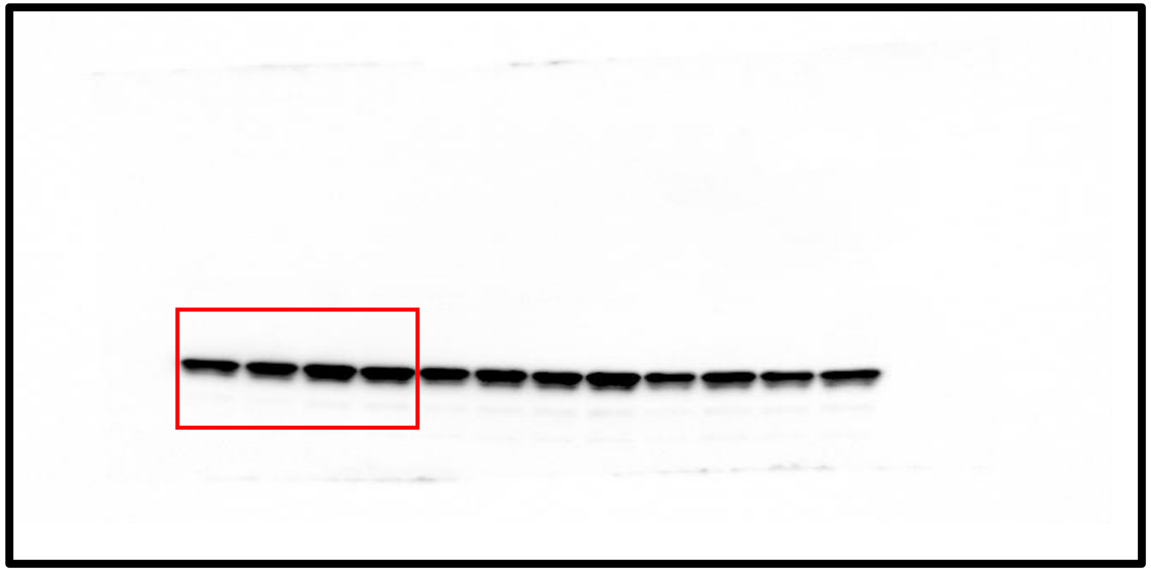

Merge with the marker

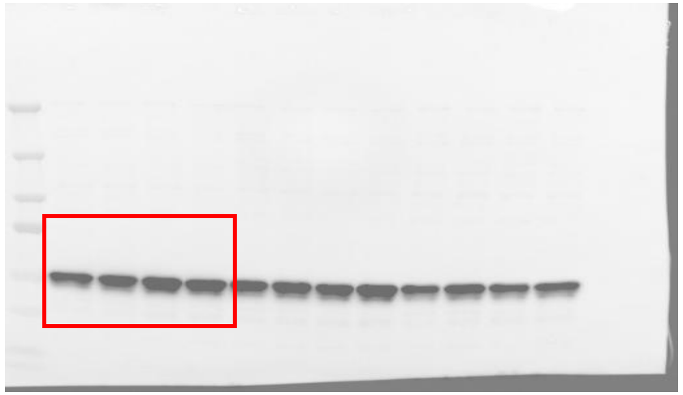

Fig.2 E. GAPDH

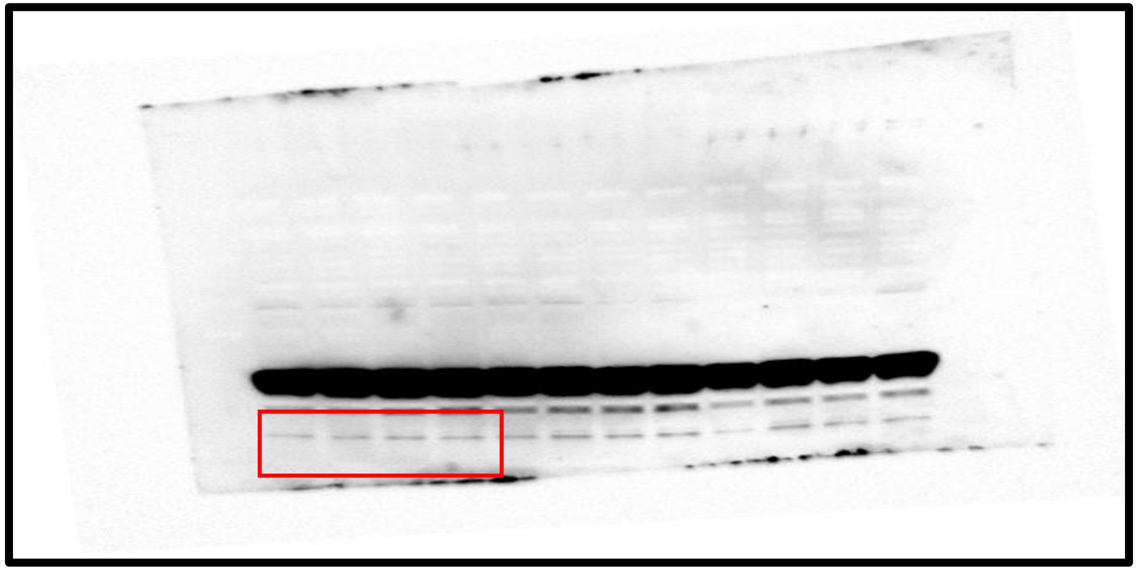

Merge with the marker

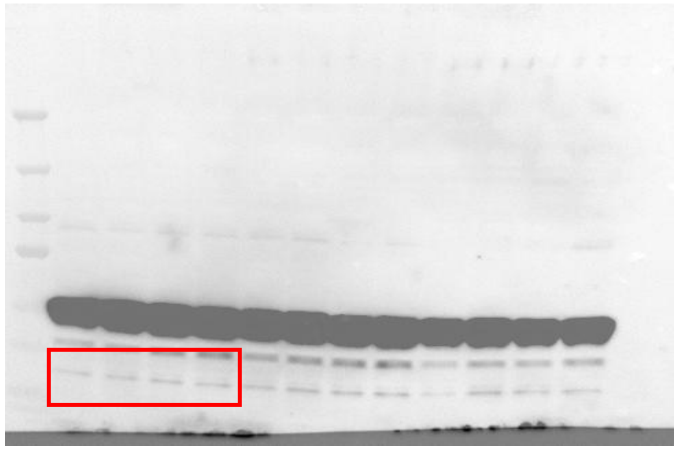

Fig.2 G. HTRA1

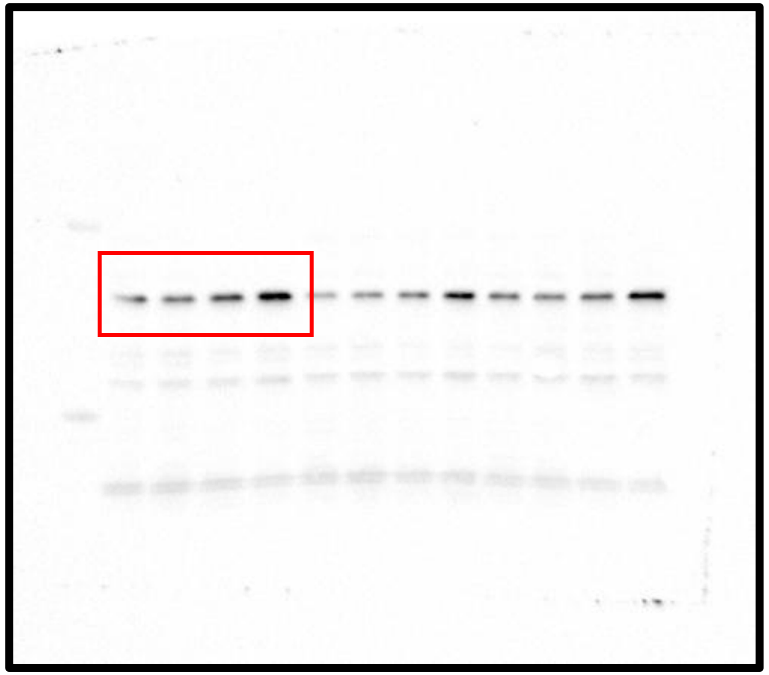

Merge with the marker

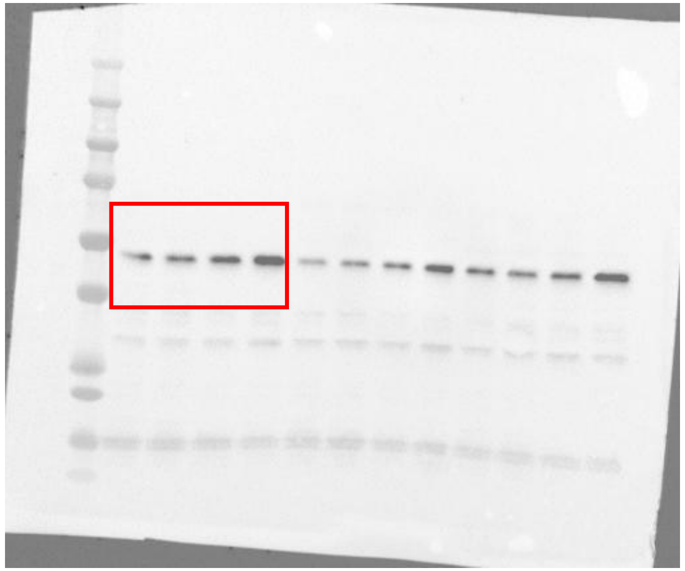

Fig.2 G. Gel staining

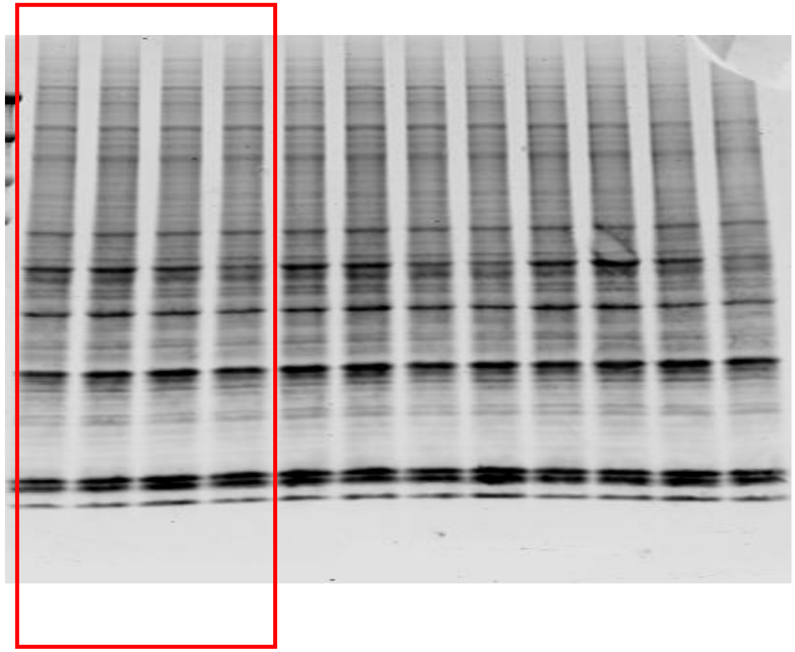

Fig.2 I. HTRA1

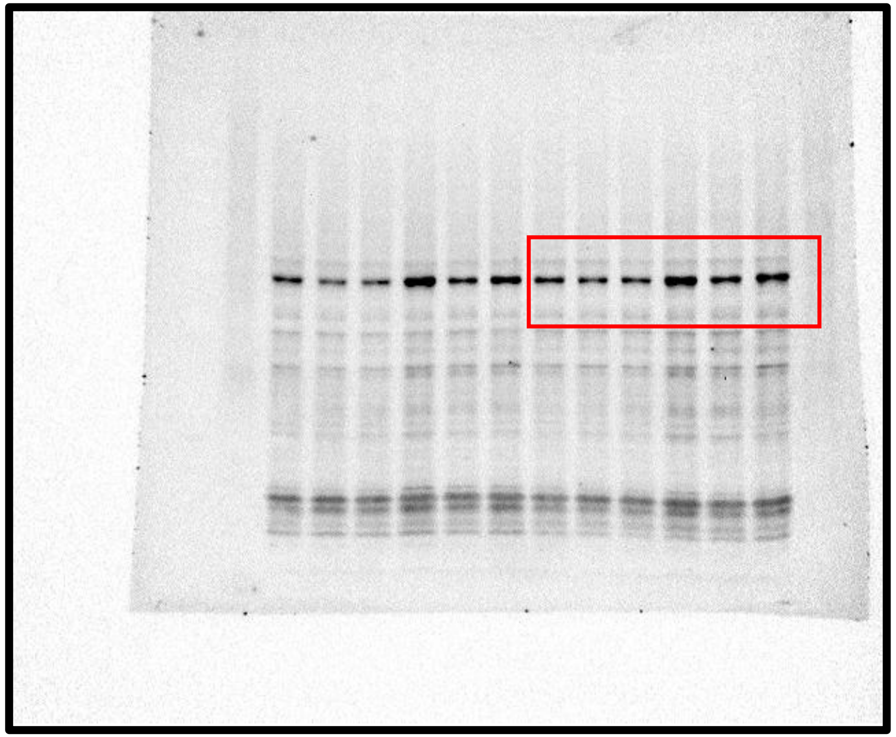

Merge with the marker

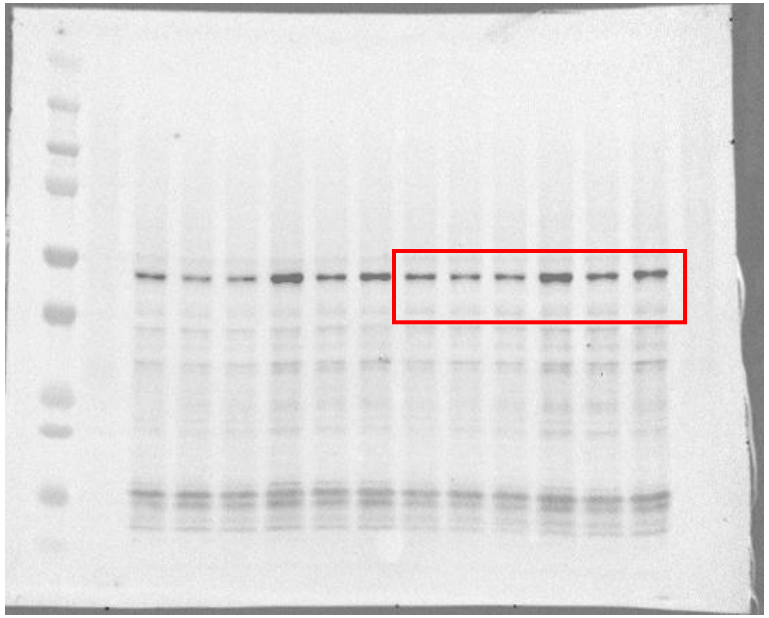

Fig.2 I. pS129-syn

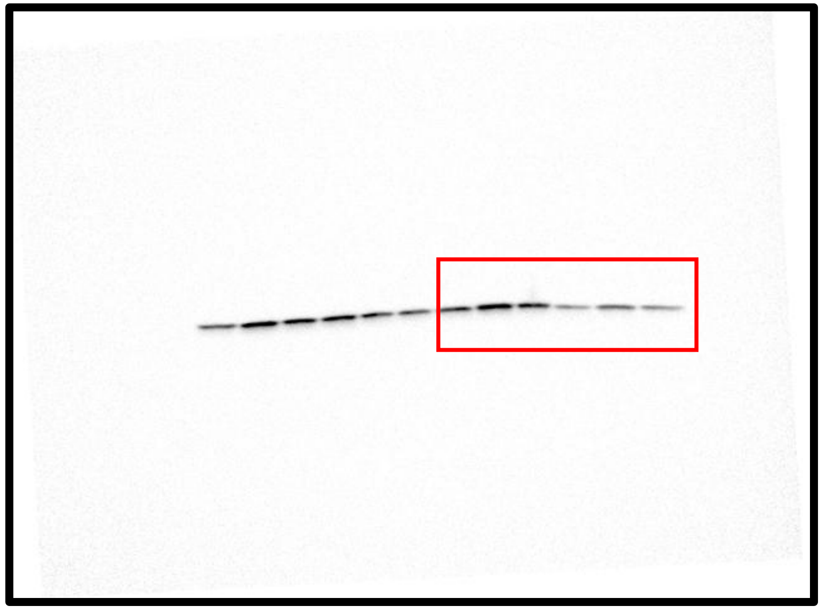

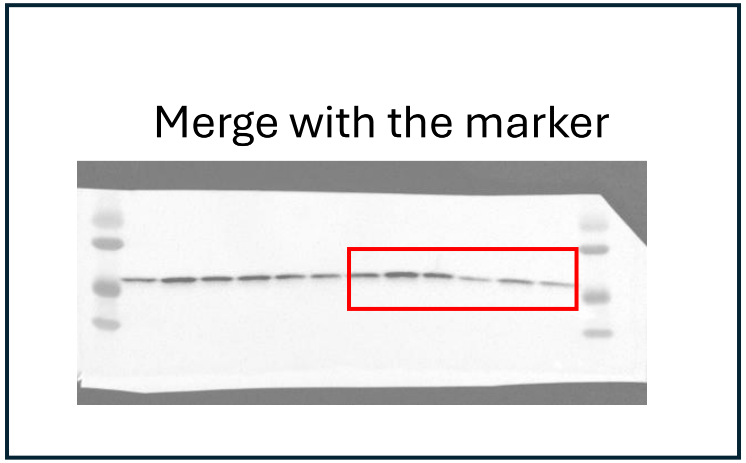

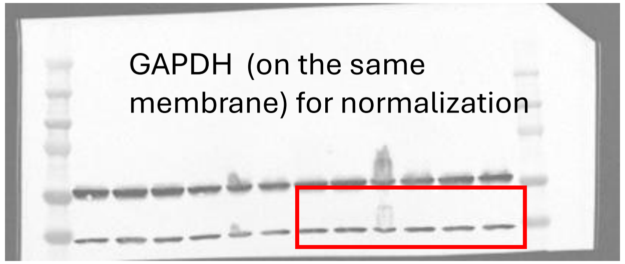

Fig.2 I. β-III-tubulin

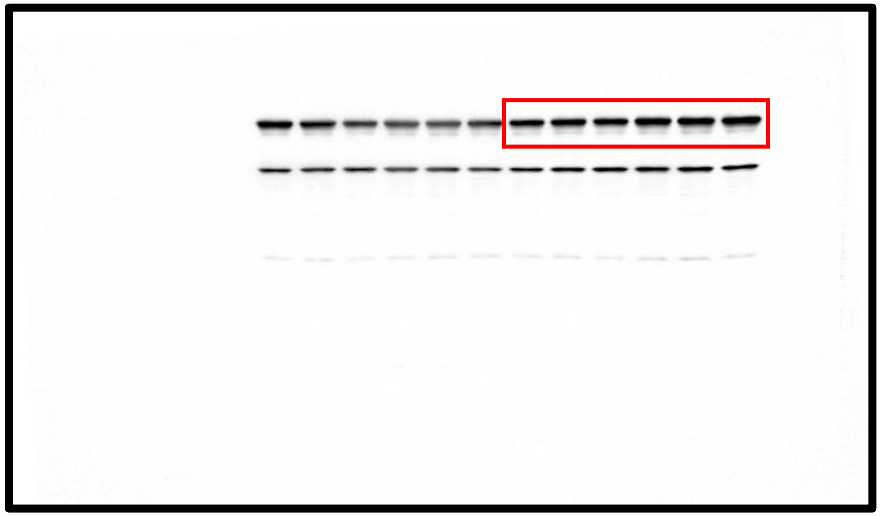

Merge with the marker

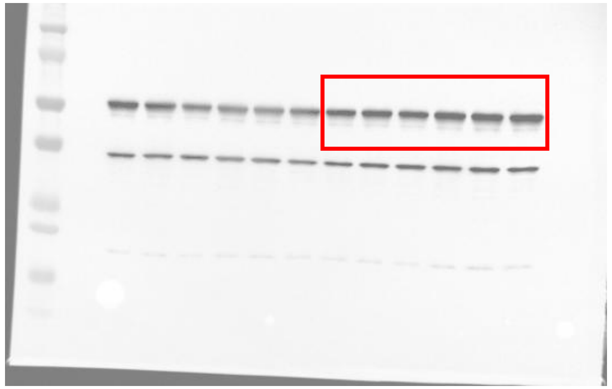

Fig.2 I. GAPDH

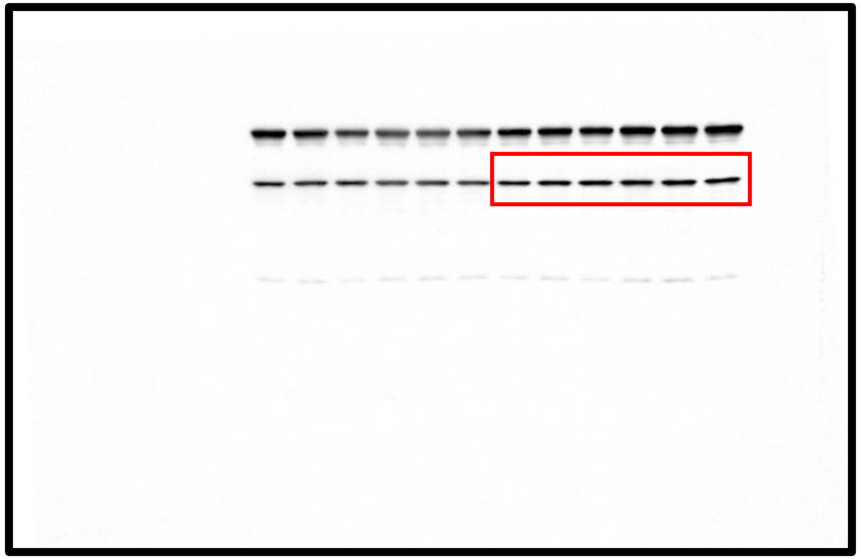

Merge with the marker

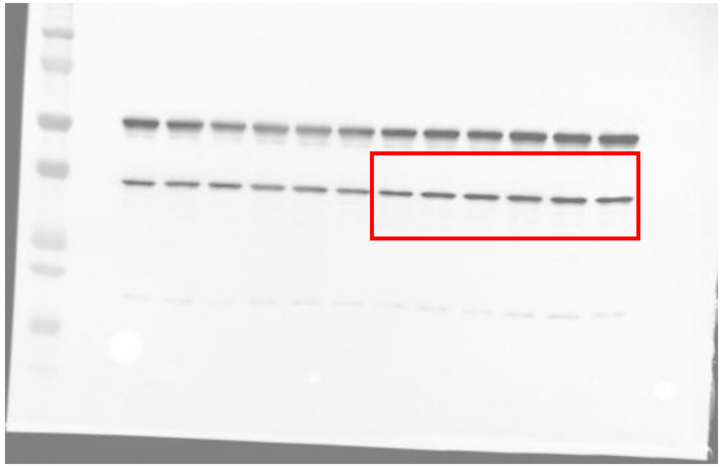

Fig.3 A. DAT

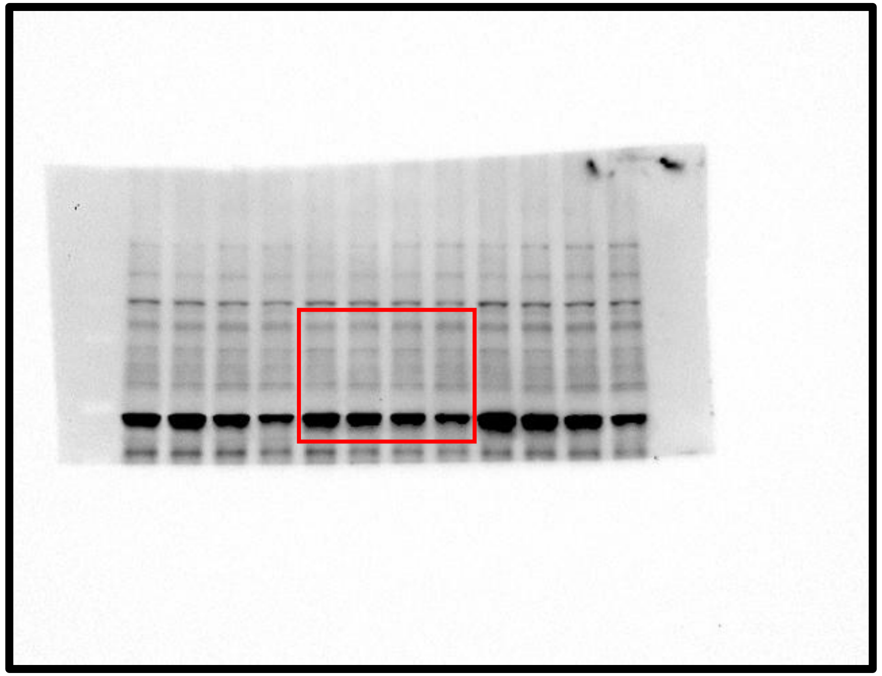

Merge with the marker

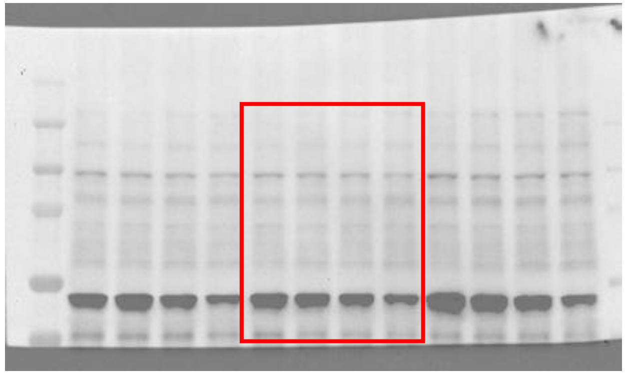

Fig.3A. β-III-tubulin

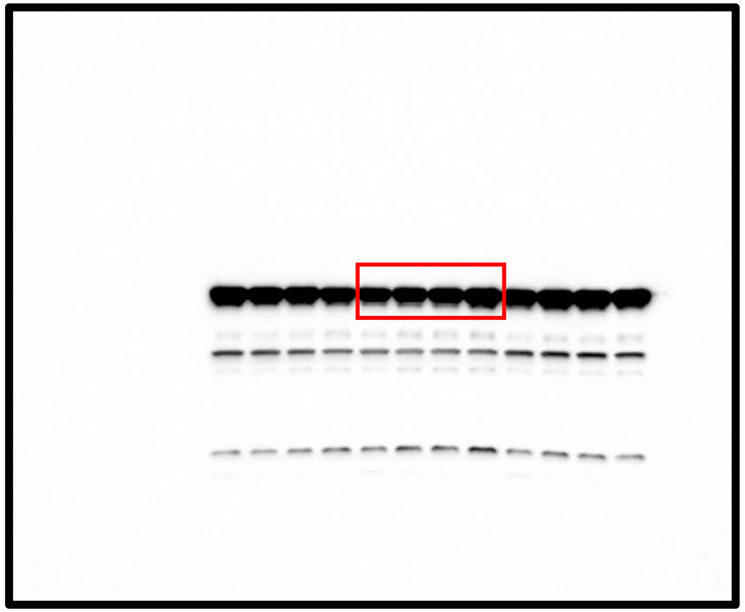

Merge with the marker

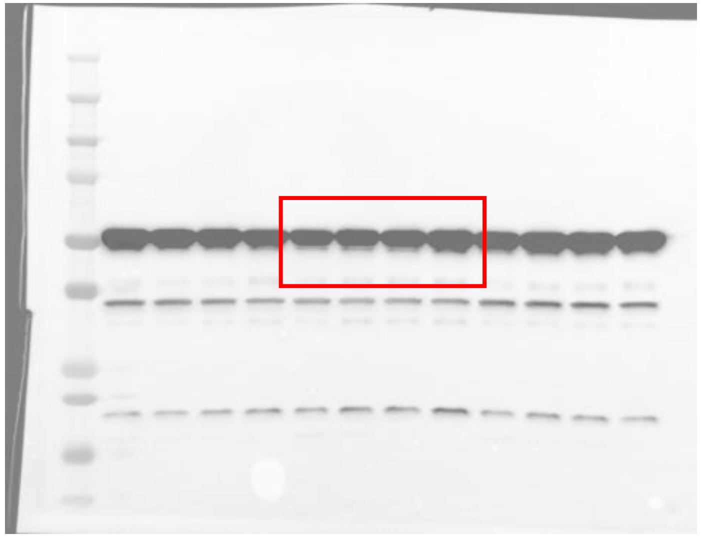

Fig.3A. GAPDH

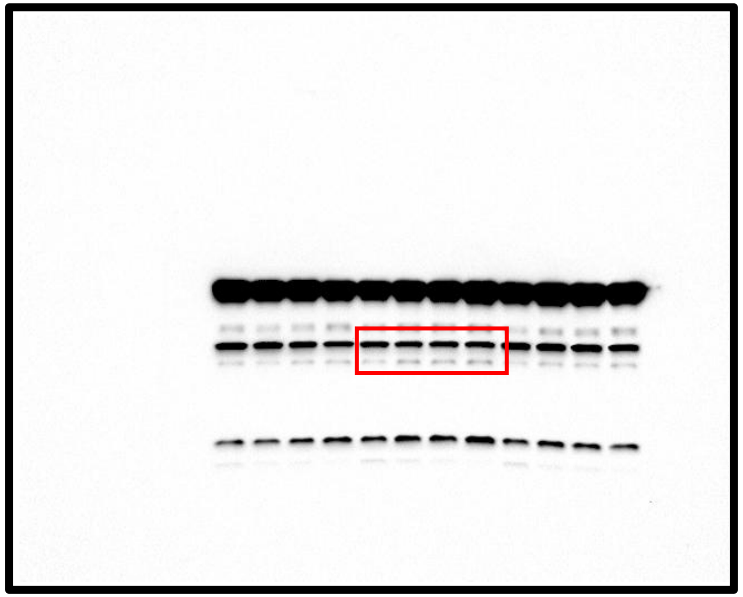

Merge with the marker

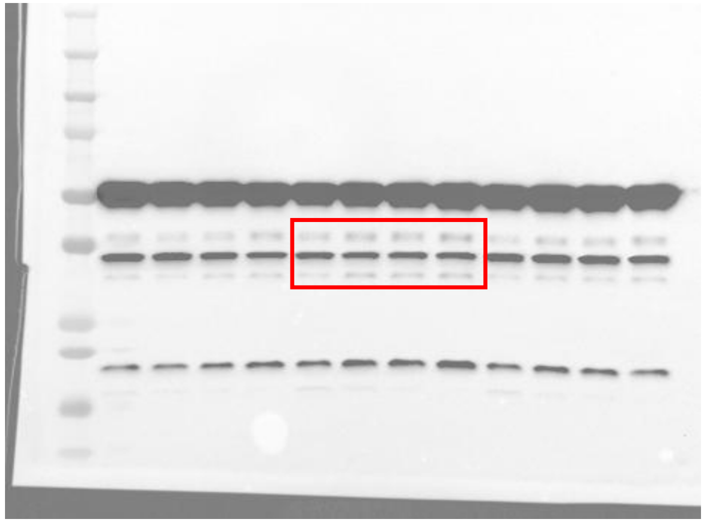

Fig.3 C. Parkin

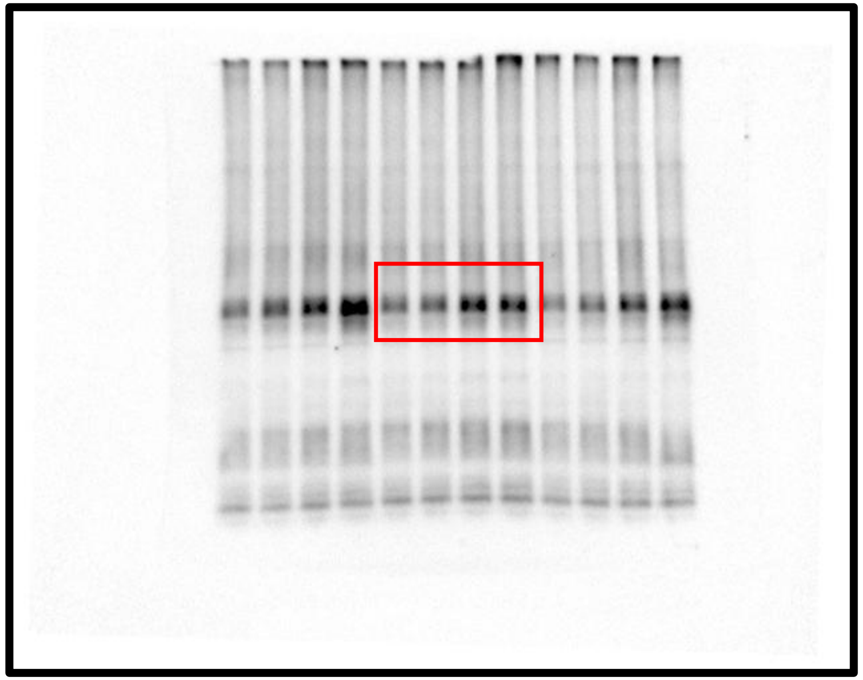

Merge with the marker

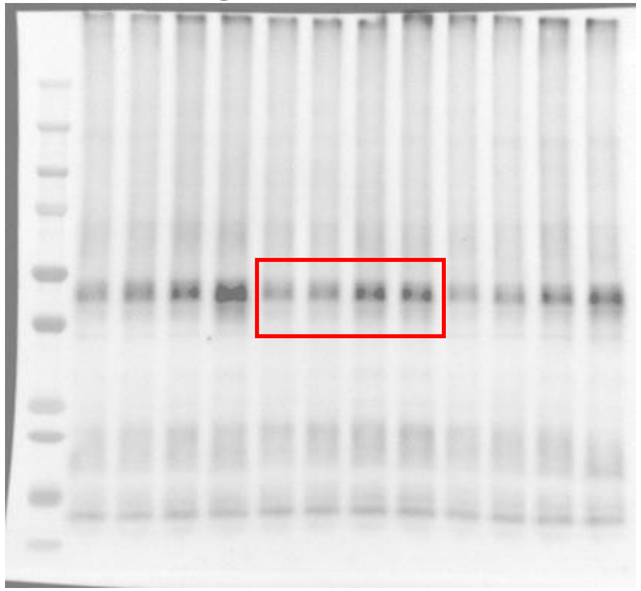

Fig.3C. β-III-tubulin

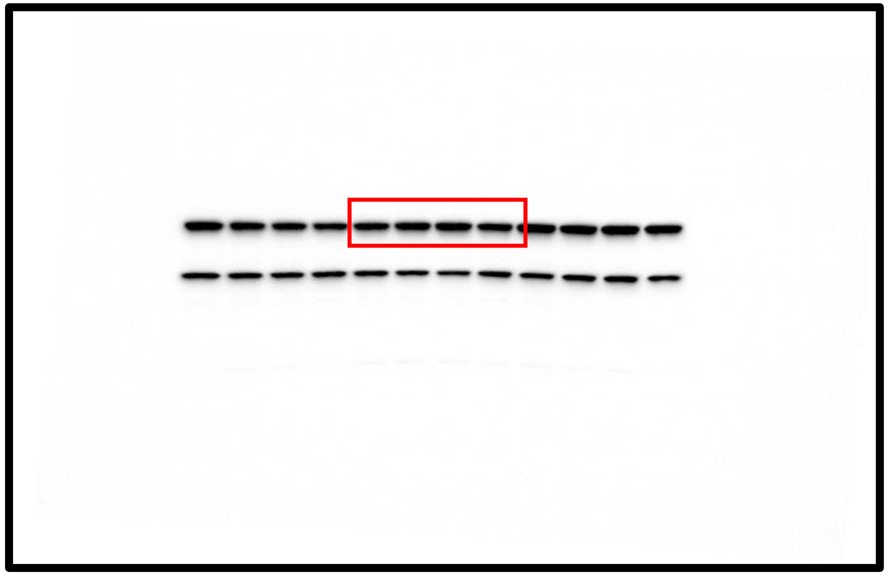

Merge with the marker

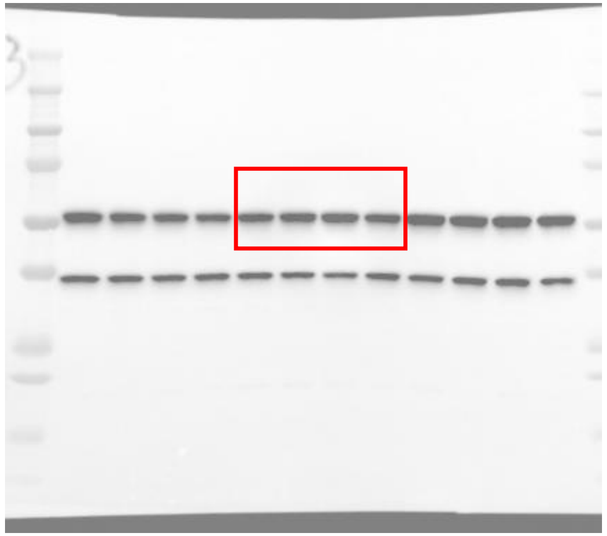

Fig.3C. GAPDH

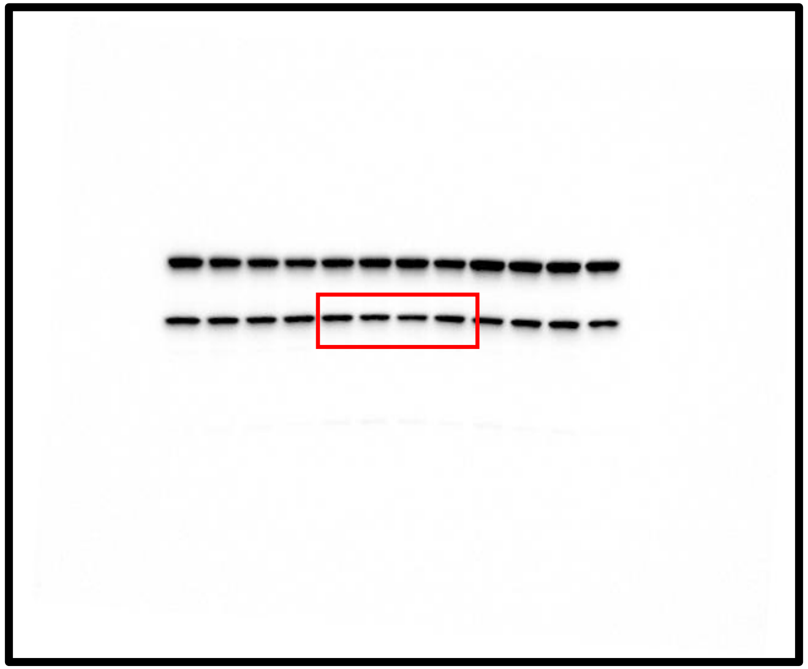

Merge with the marker

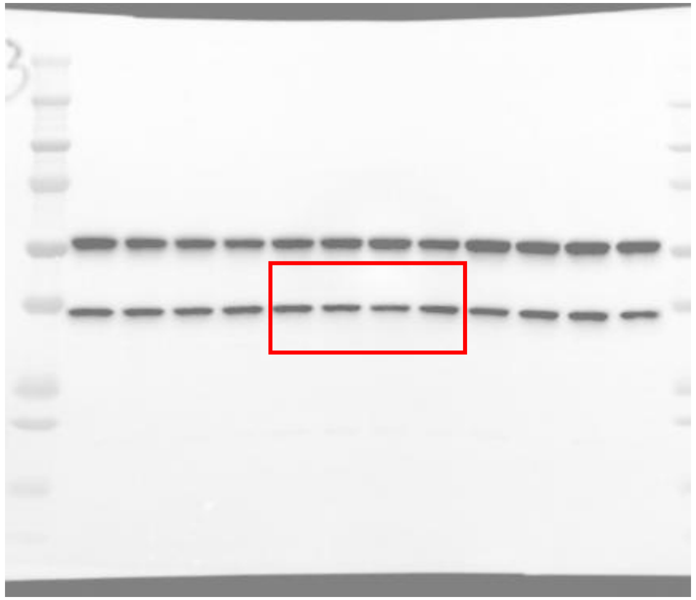

Fig.3 E. SYNJ1

Merge with the marker

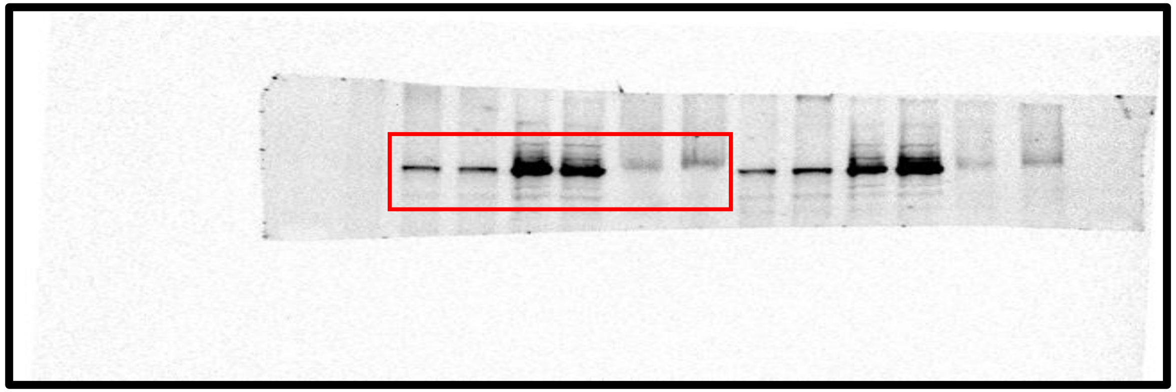

The membrane had been cut below 75kD before imaging

Fig.3E. SYP

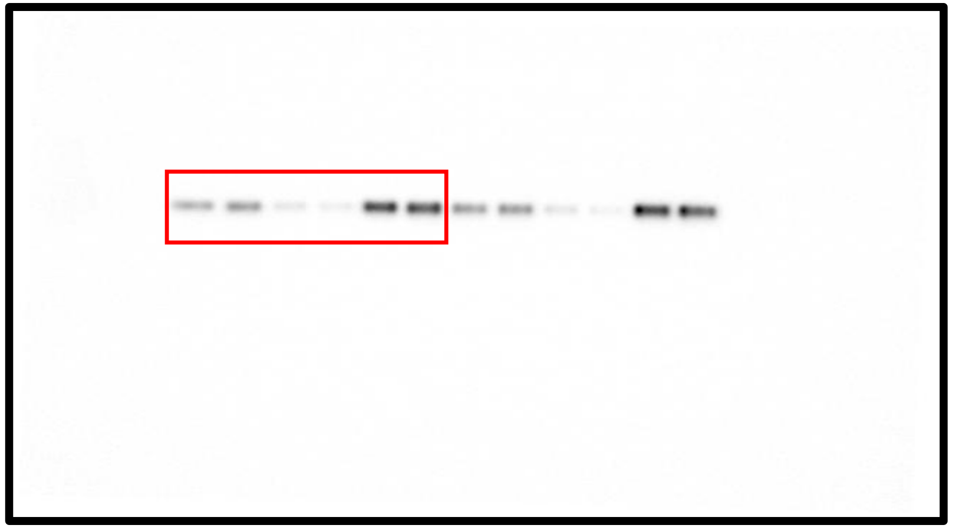

Merge with the marker

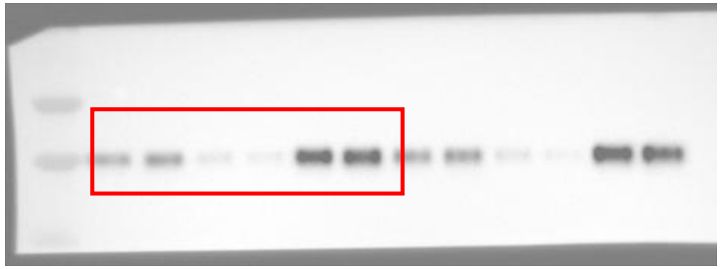

The membrane had been cut above 75kD before imaging

Fig.3E. GAPDH

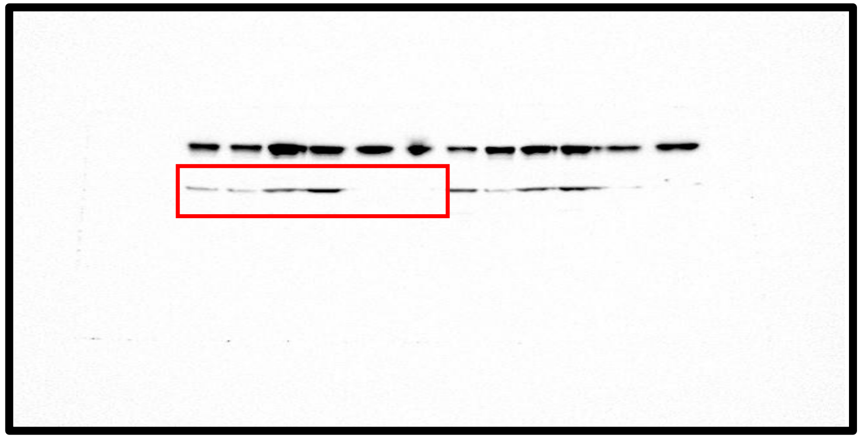

Merge with the marker

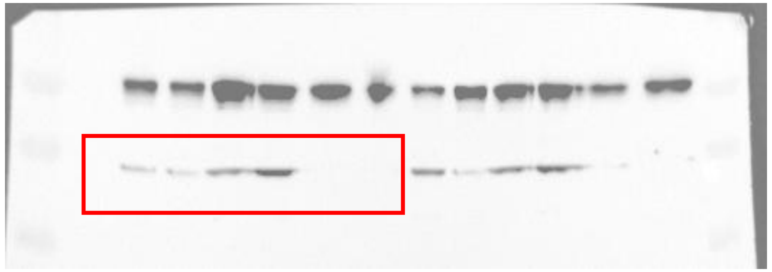

The membrane had been cut above 75kD before imaging

Fig.3 G. SYNJ1

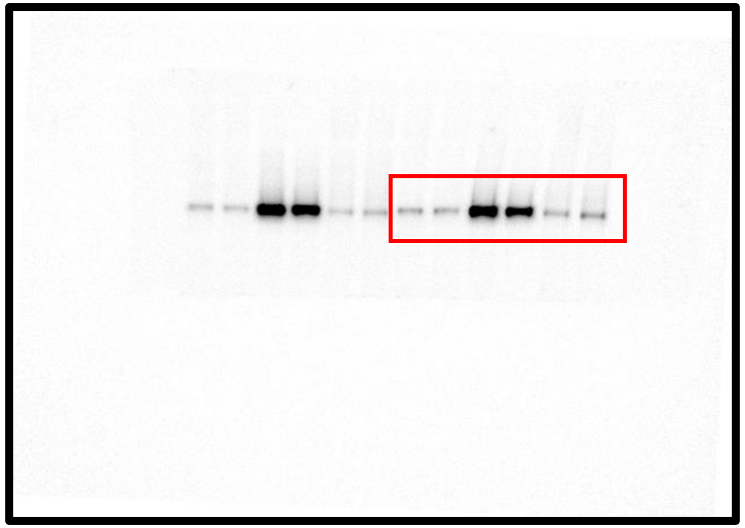

Merge with the marker

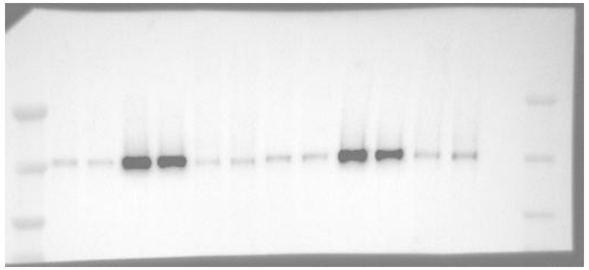

The membrane had been cut below 75kD before imaging

Fig.3G. SYP

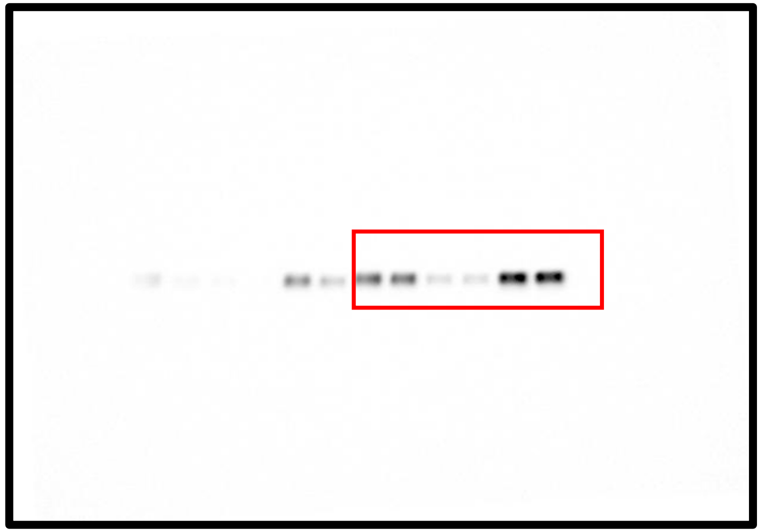

Merge with the marker

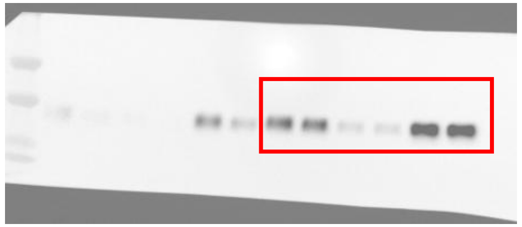

The membrane had been cut above75kD before imaging

Fig.3G. GAPDH

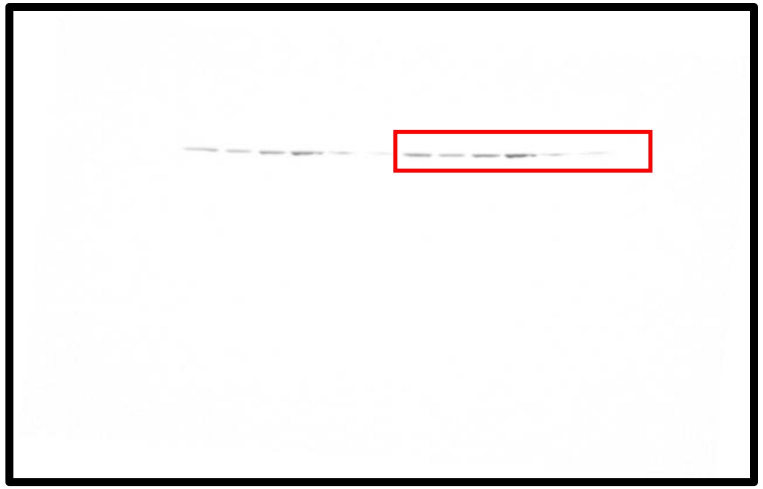

Merge with the marker

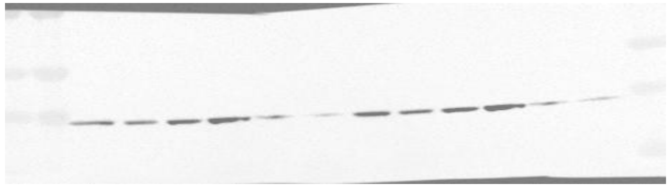

The membrane had been cut above75kD before imaging

Fig.3 I. SYNJ1

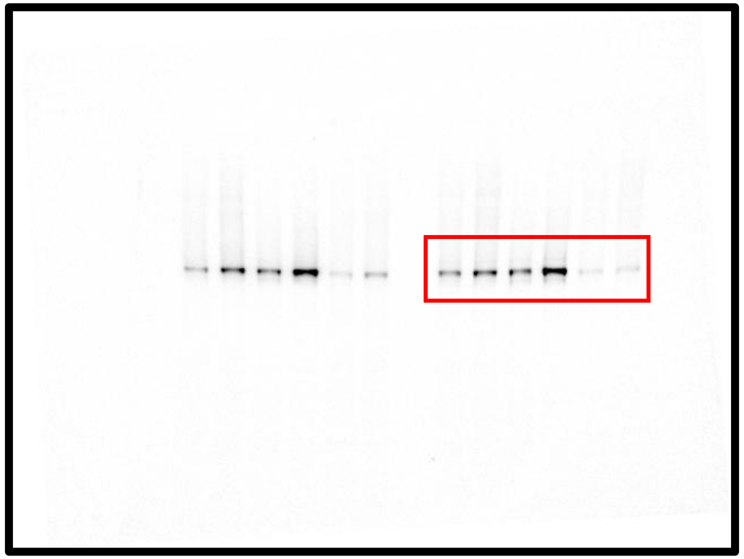

Merge with the marker

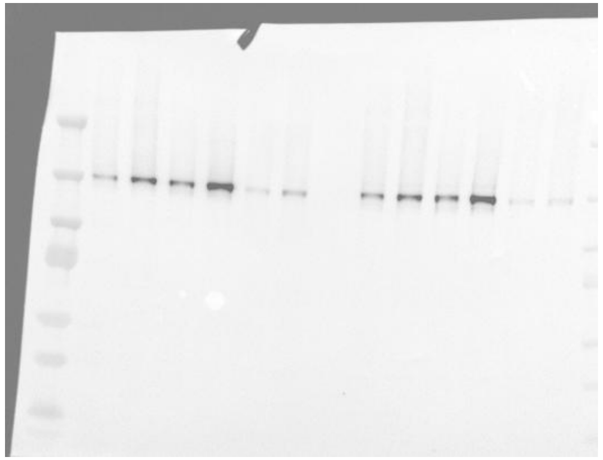

Fig.3I. SYP

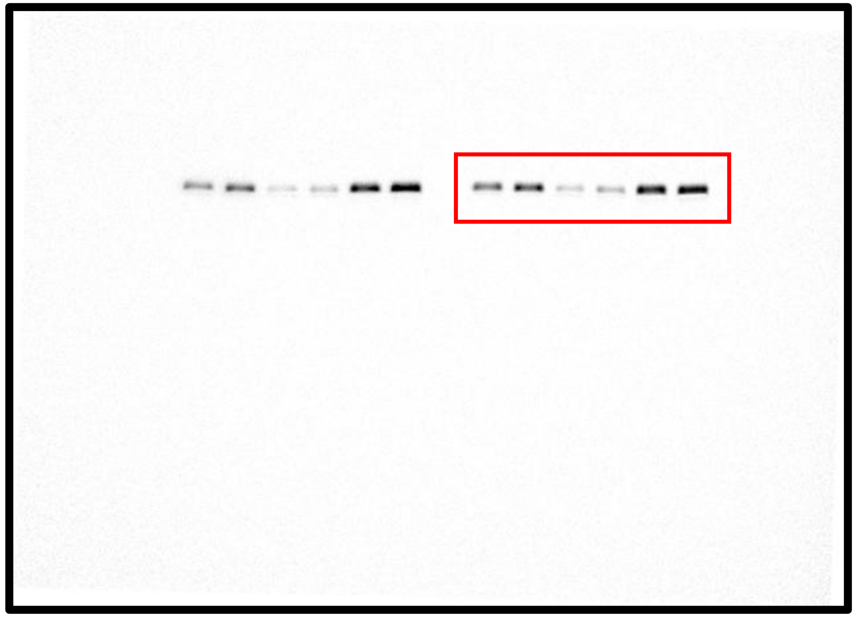

Merge with the marker

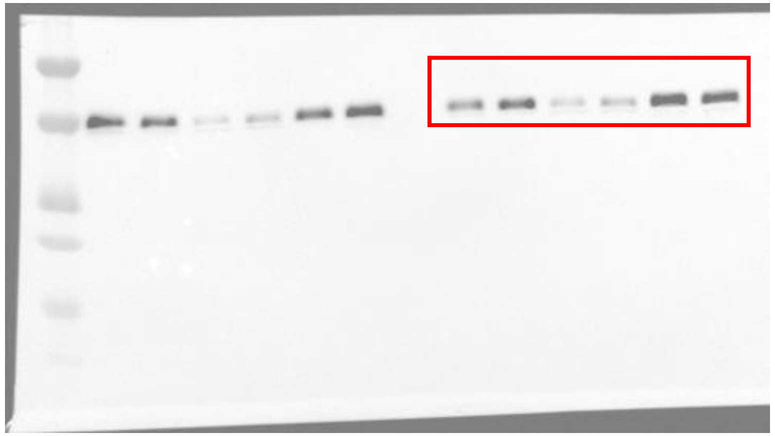

The membrane had been cut above75kD before imaging

Fig.3I. GAPDH

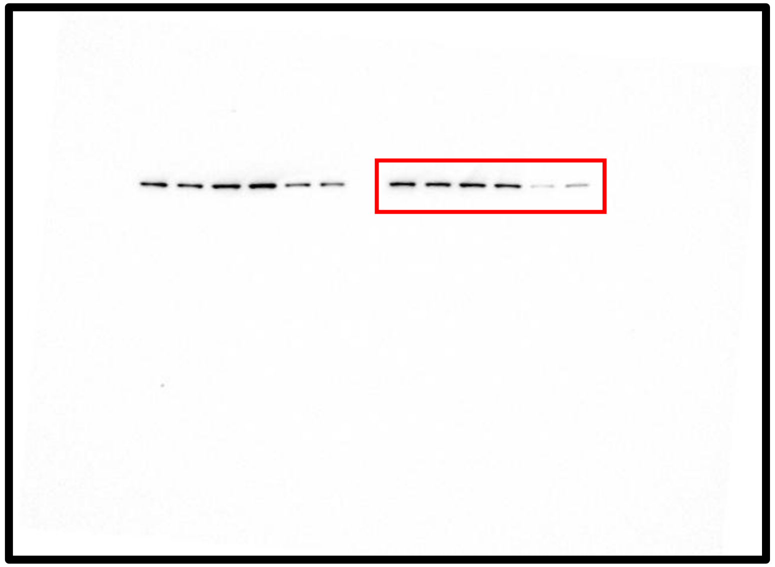

Merge with the marker

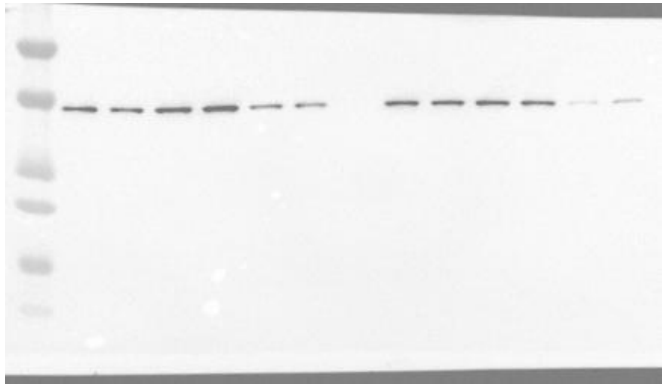

The membrane had been cut above75kD before imaging

Fig.4 A. pS129-syn

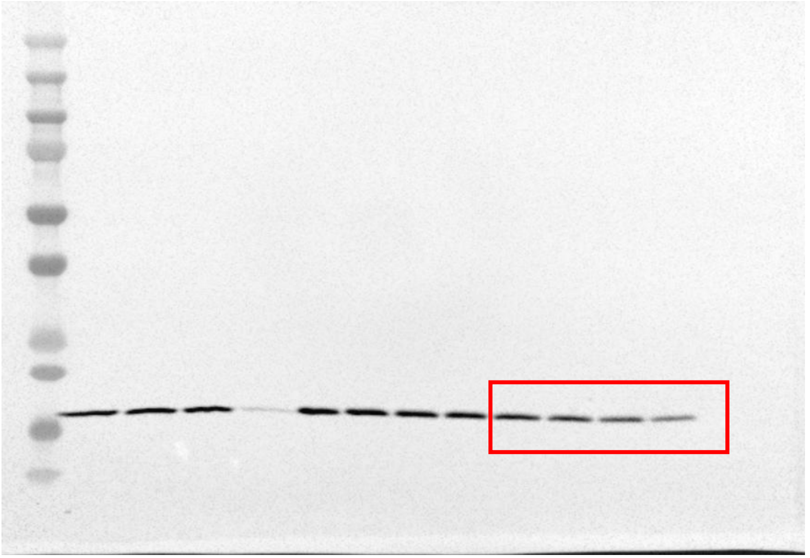

Fig.4 A. Parkin

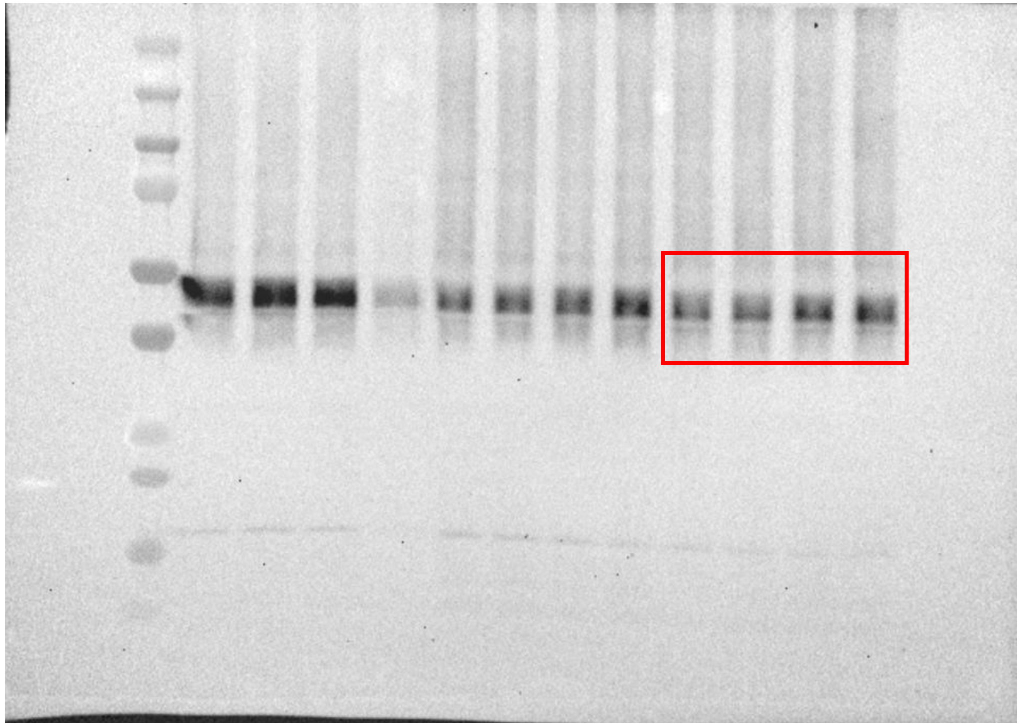

Fig.4 A. β-III-tubulin

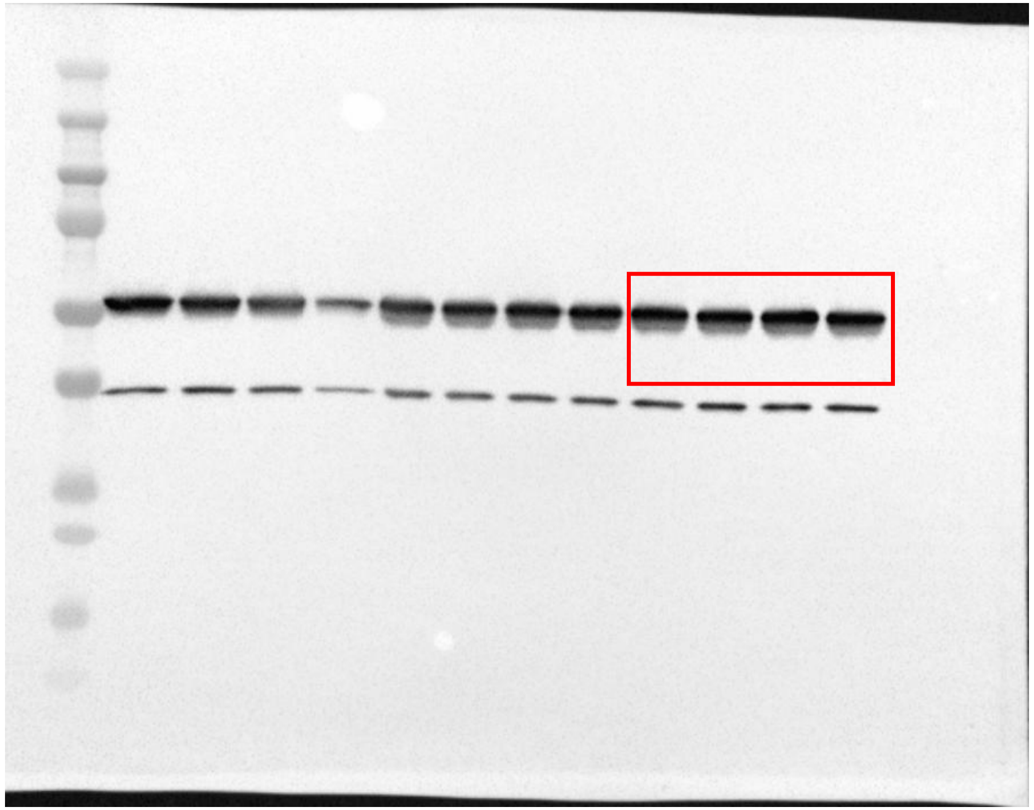

Fig.4 A. GAPDH

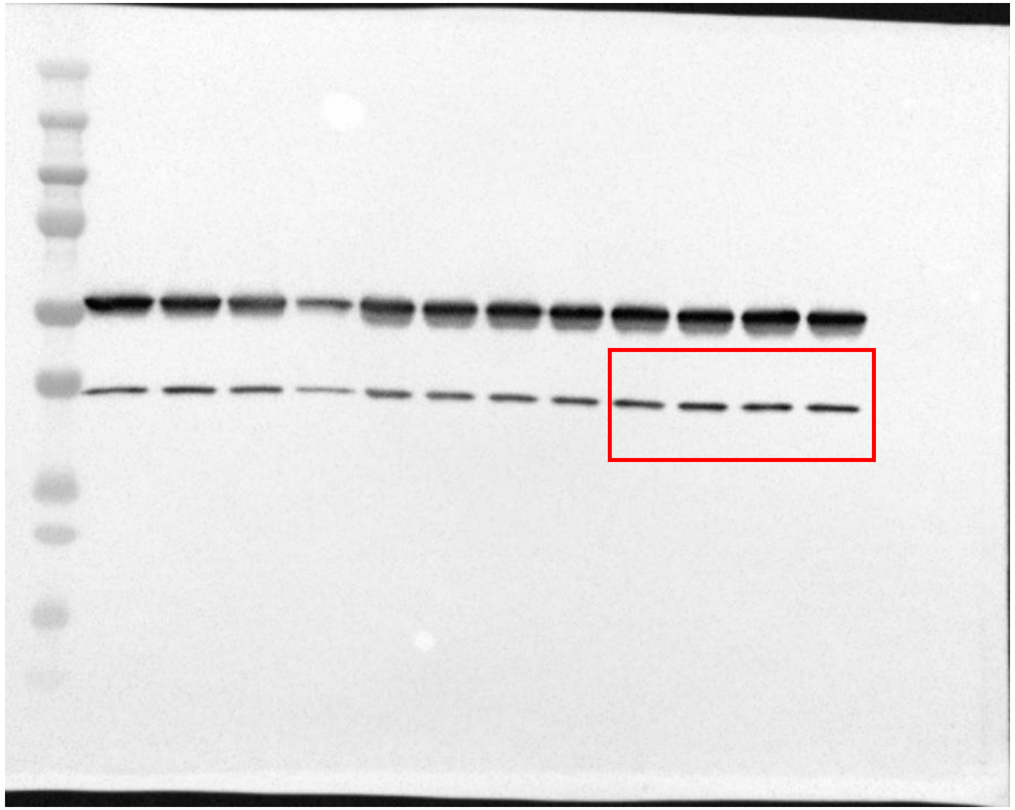

Fig.4 C. pS129-syn

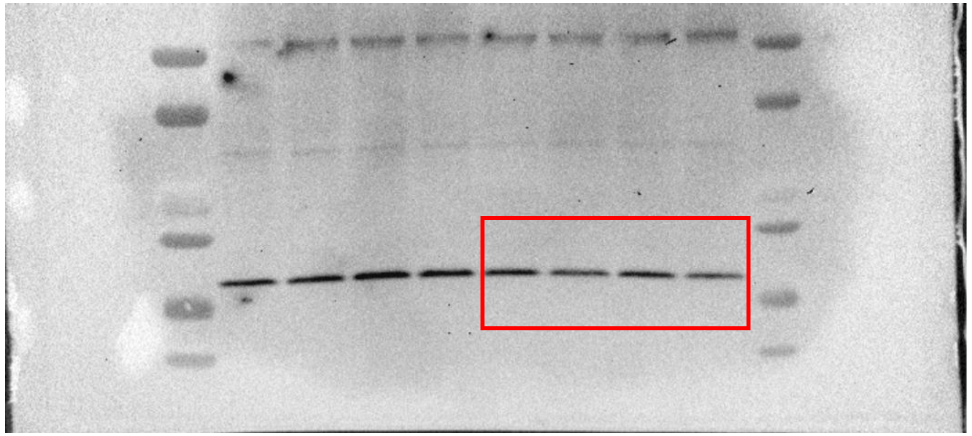

Fig.4 C. HTRA1

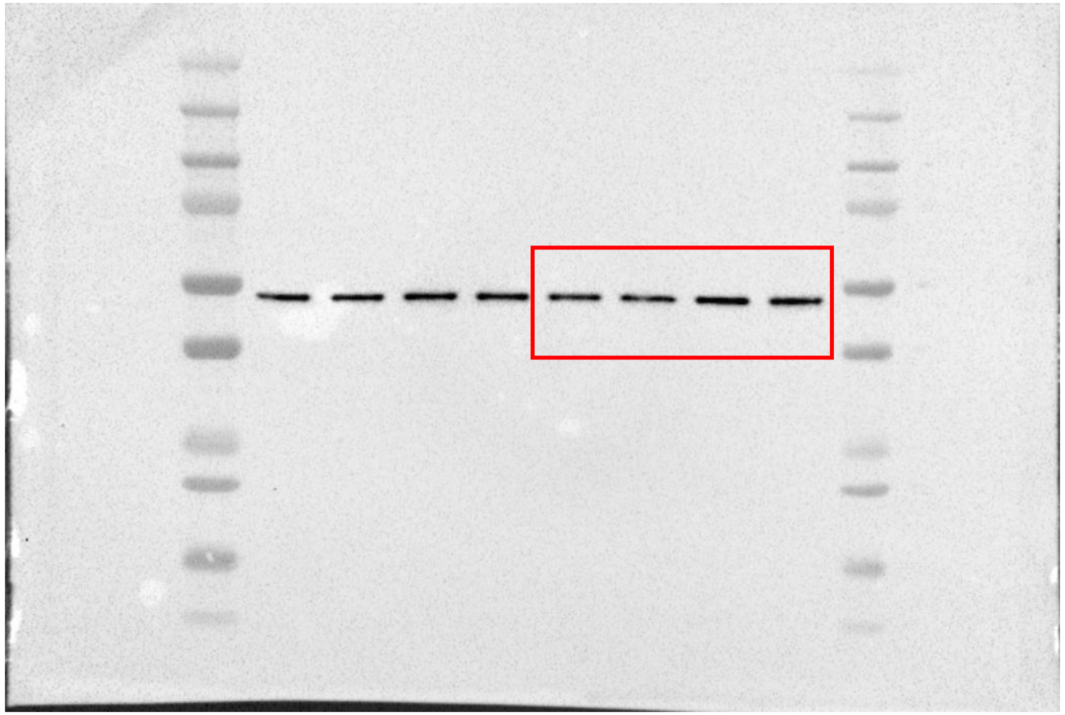

Fig.4 C. Gel staining

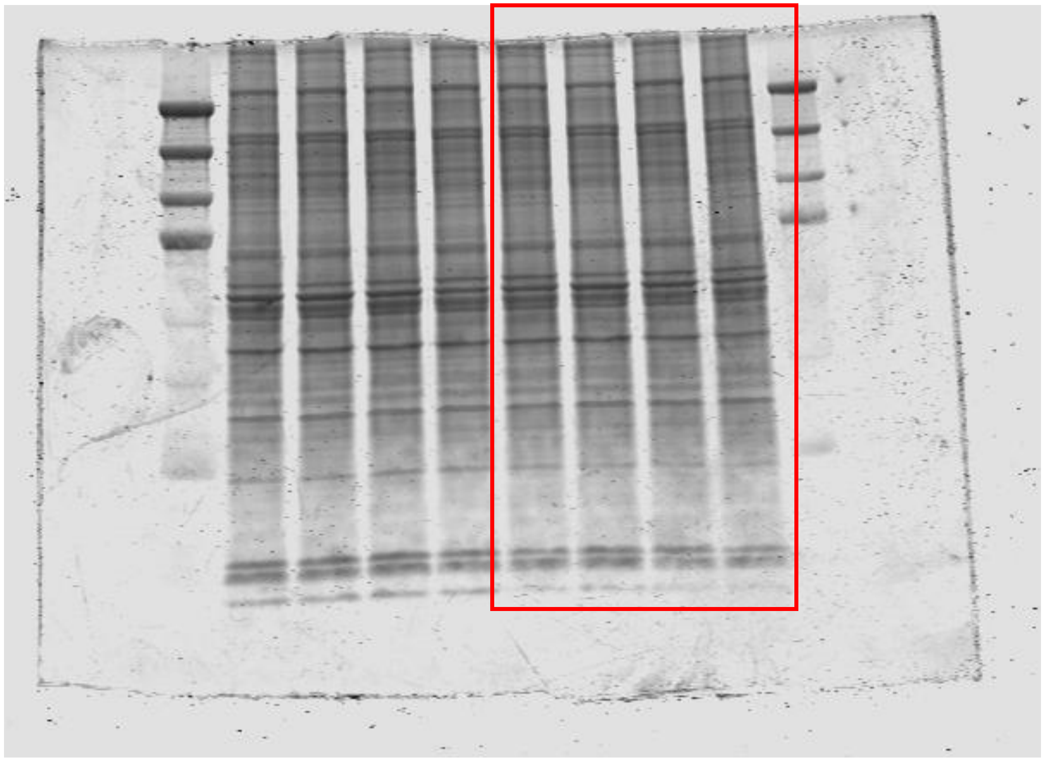

Fig.4 E. pS129-syn

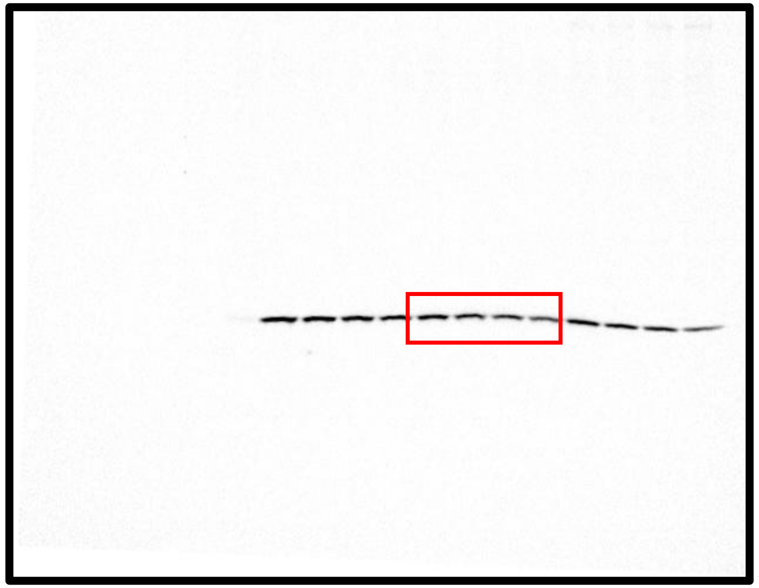

Merge with the marker

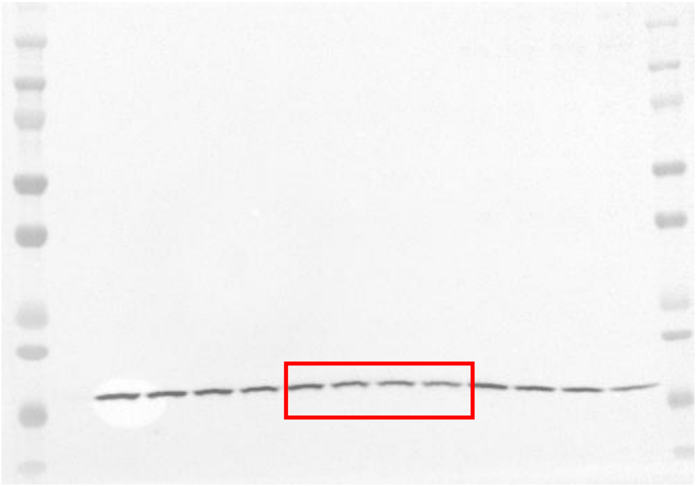

Fig.4 E. Parkin

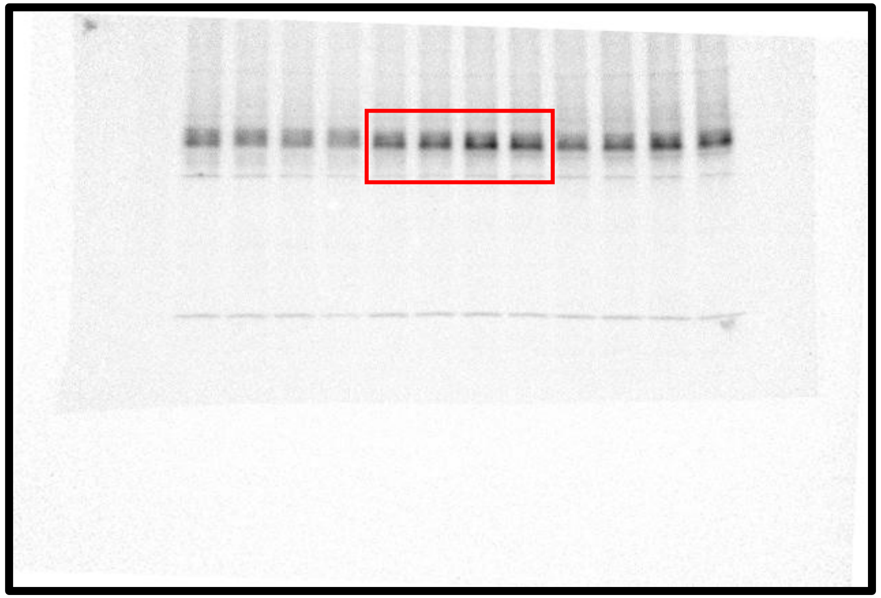

Merge with the marker

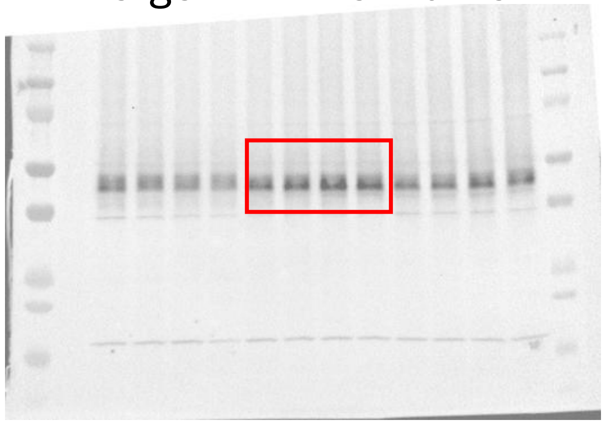

Fig.4 E. β-III-tubulin

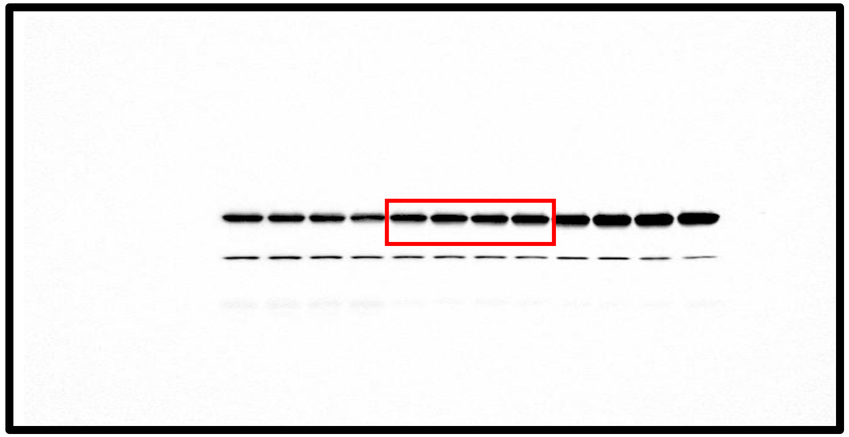

Merge with the marker

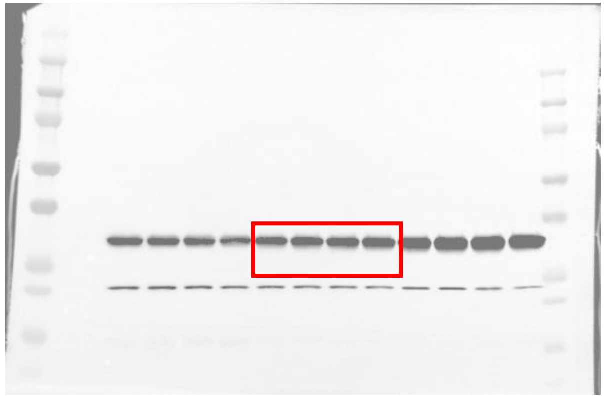

Fig.4 E. GAPDH

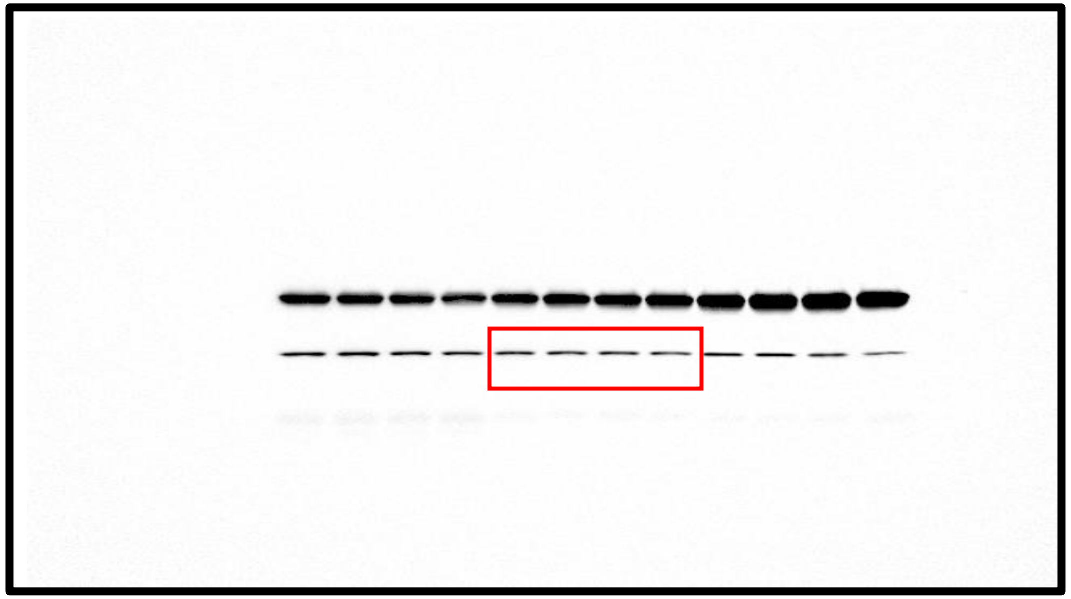

Merge with the marker

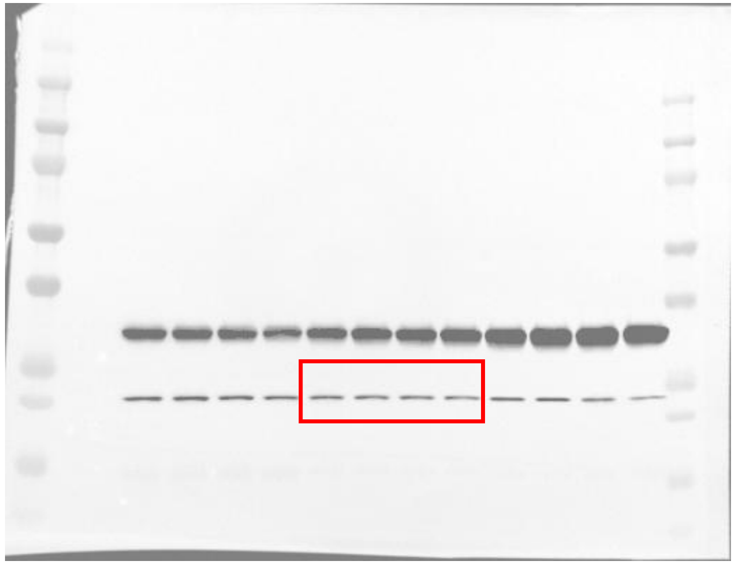

Fig.4 G. pS129-syn

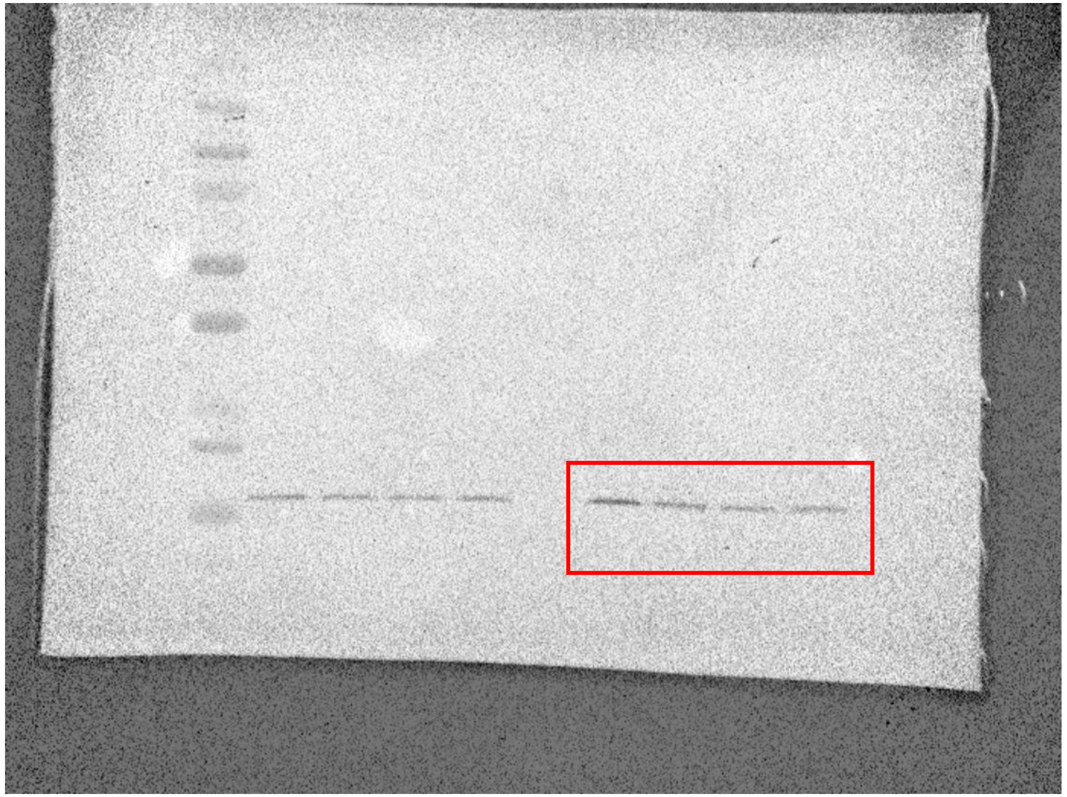

Fig.4 G. HTRA1

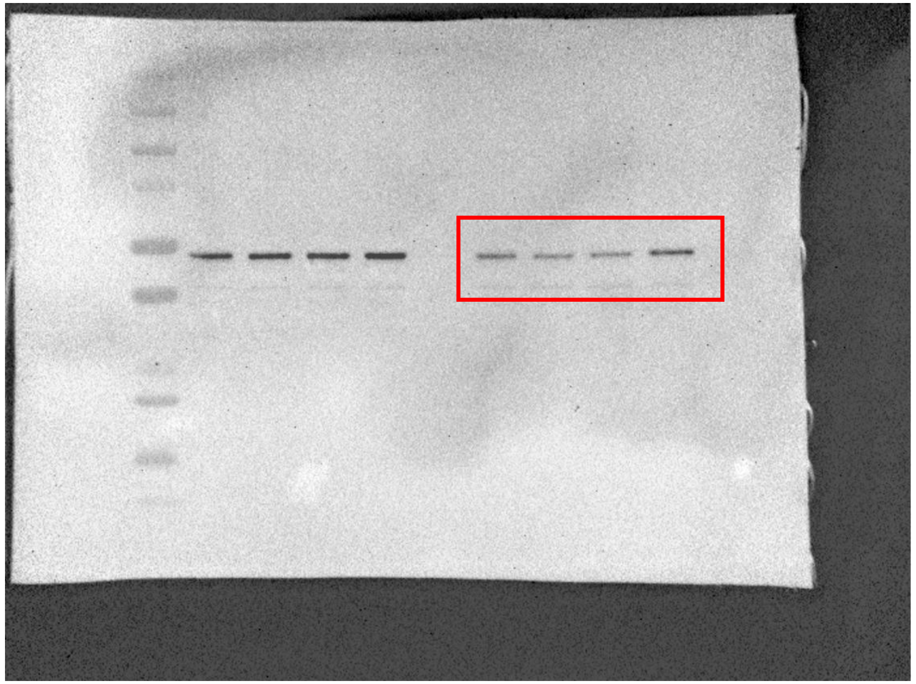

Fig.4 G. Gel staining

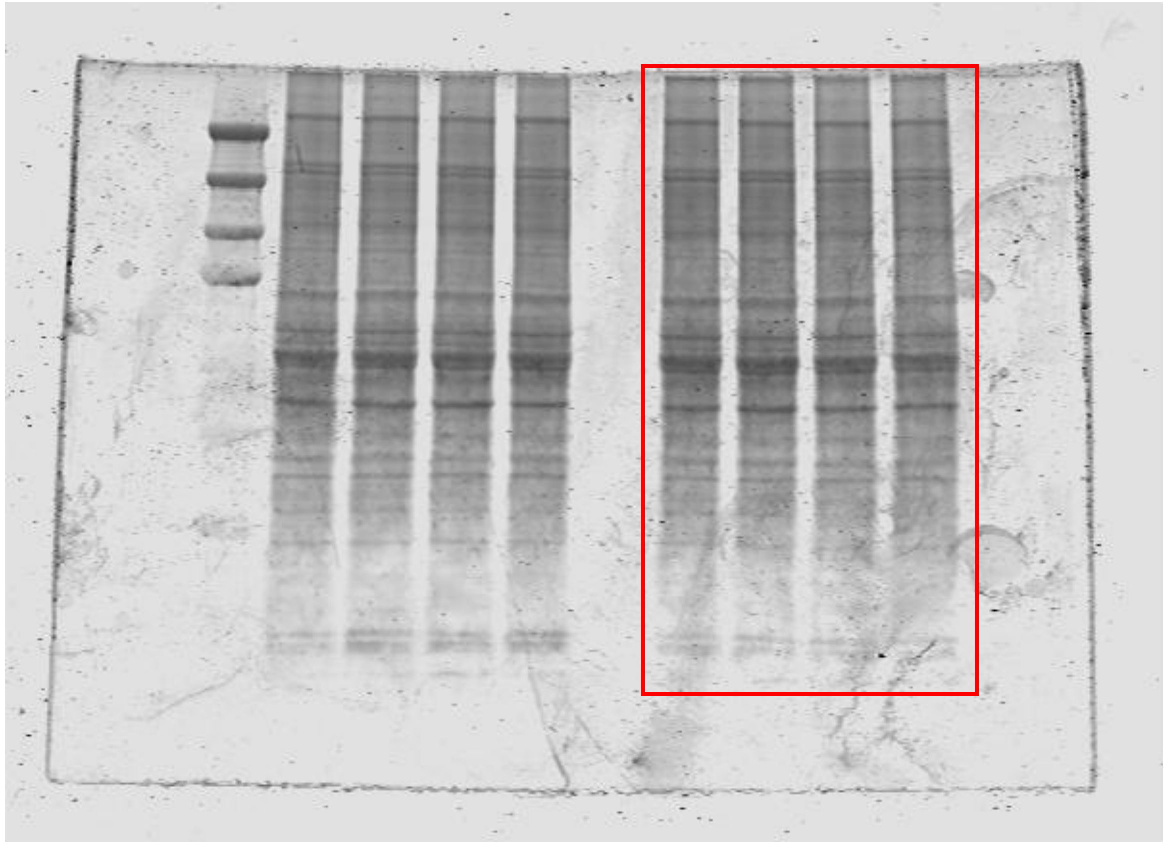

Fig.4I. pS129-syn

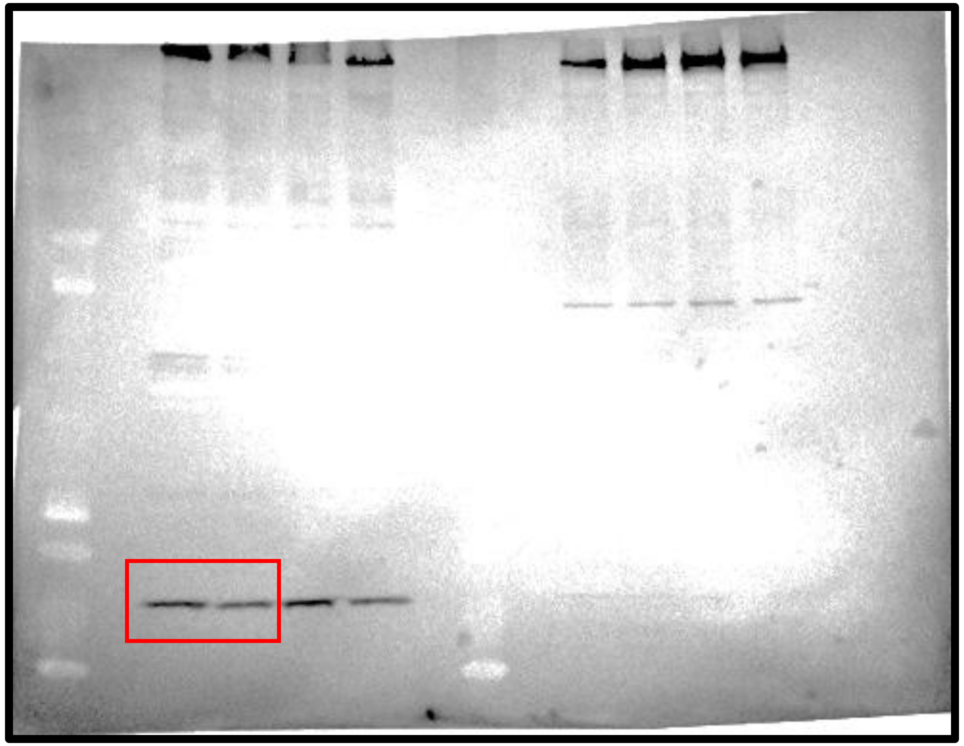

Fig.4I. Parkin

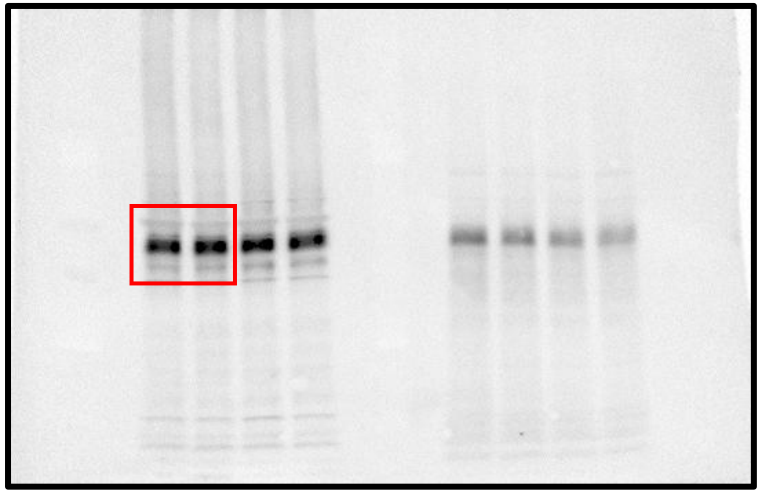

Merge with the marker

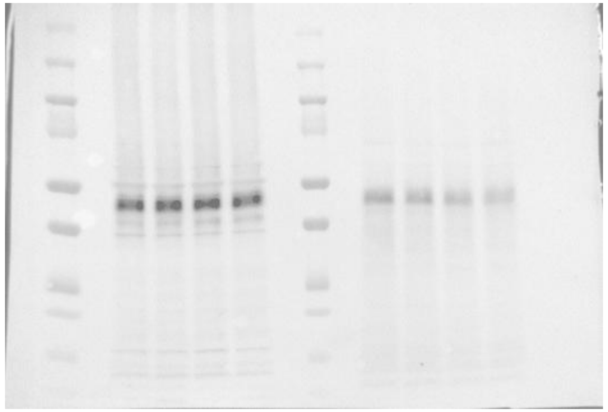

Fig.4I. β-III-tubulin

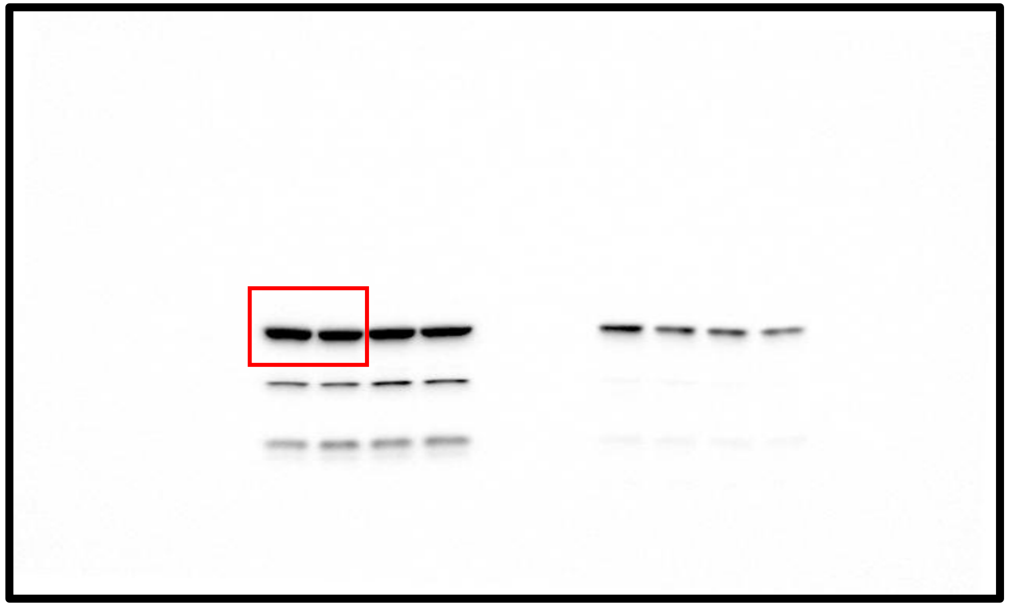

Merge with the marker

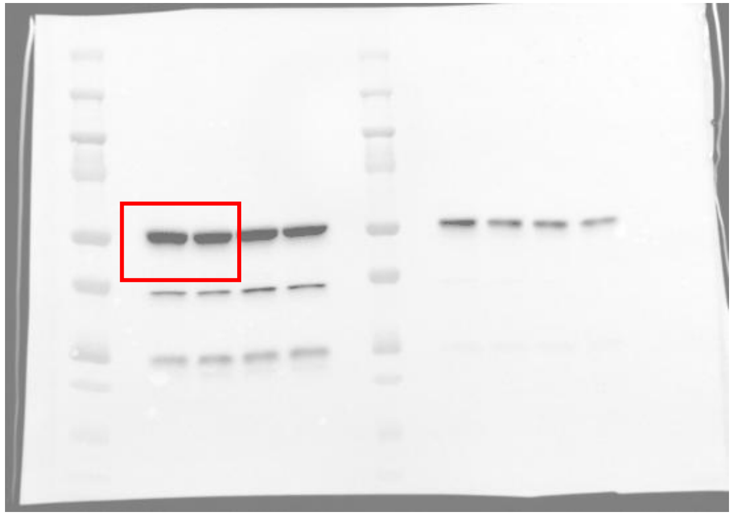

Fig.4I. GAPDH

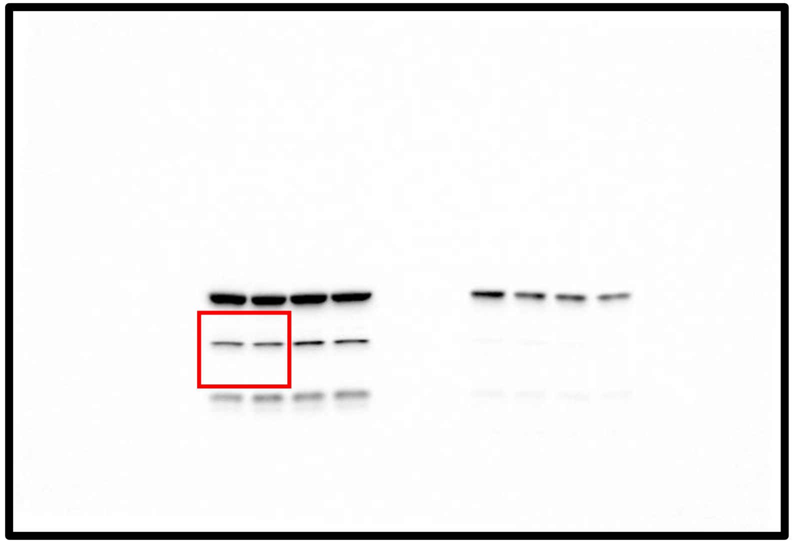

Merge with the marker

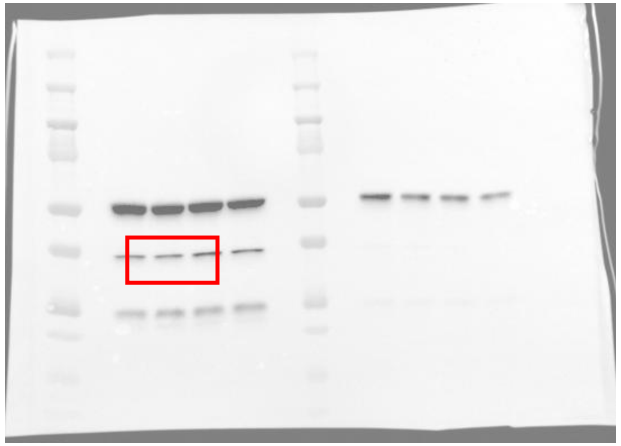

Fig.4K. pS129-syn

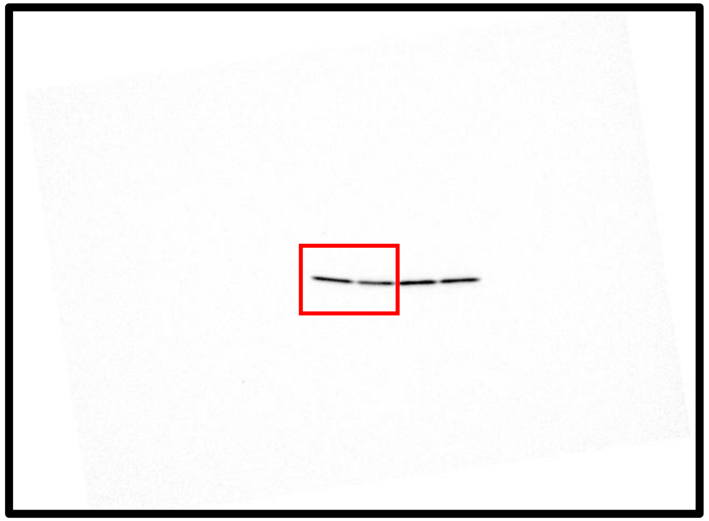

Merge with the marker

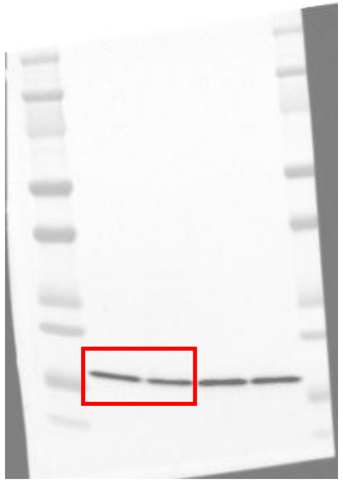

Total protein (the same membrane) for normalization

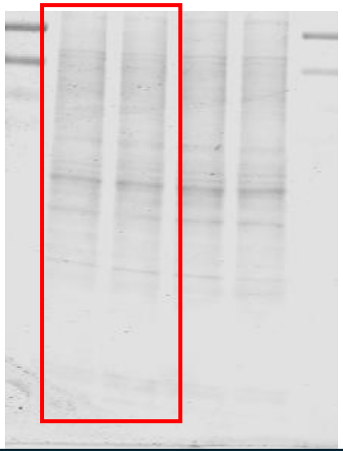

Fig.4K. HTRA1

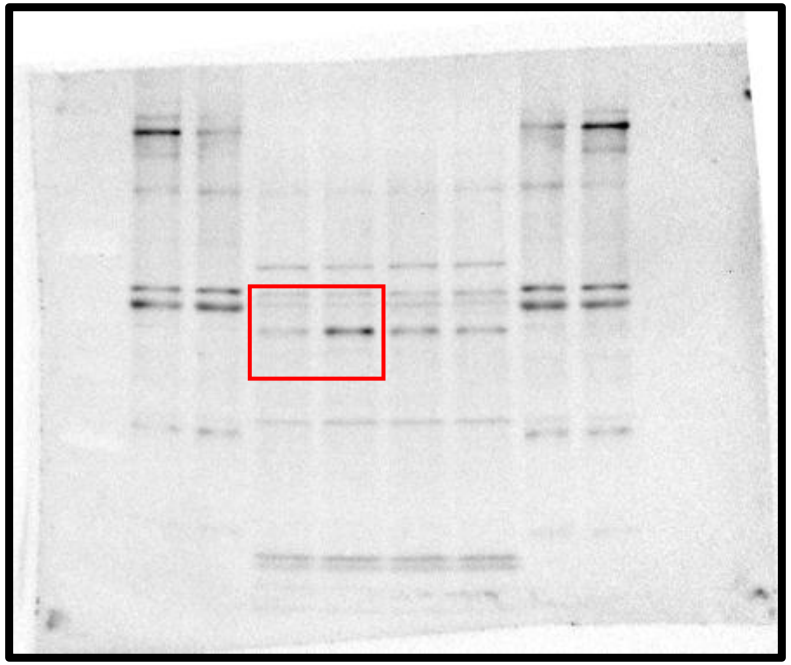

Merge with the marker

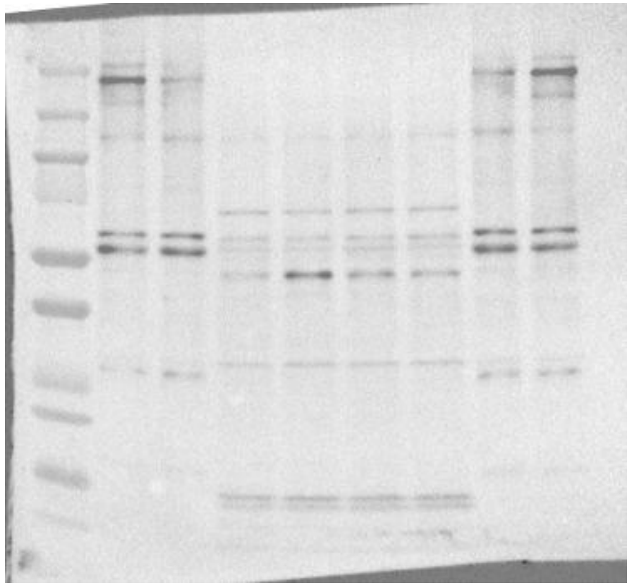

Fig.4K. Gel staining

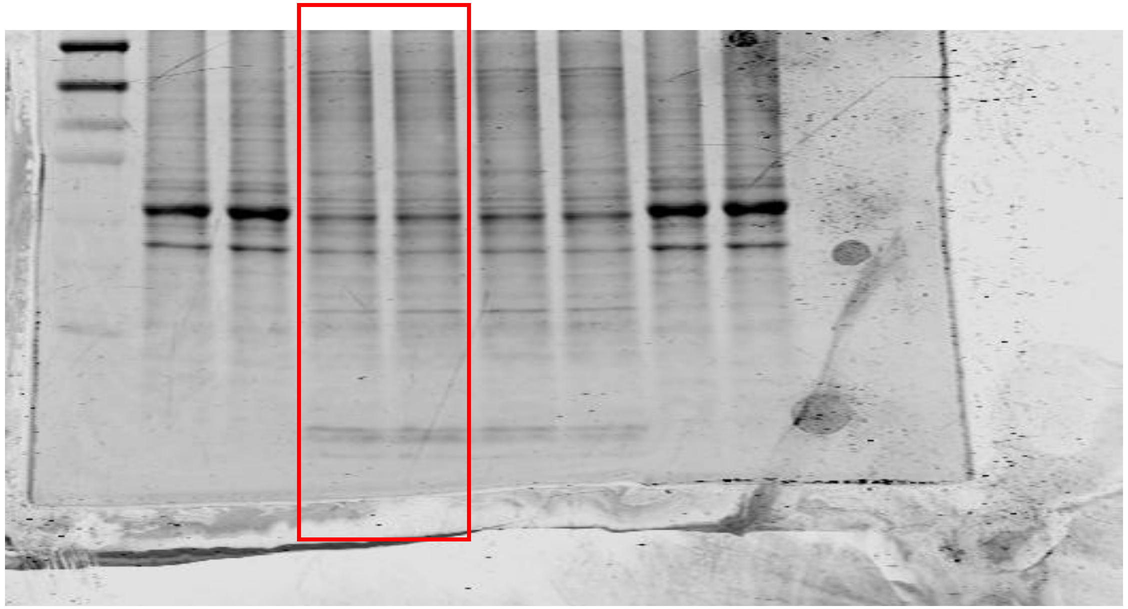

Fig.4M. pS129-syn

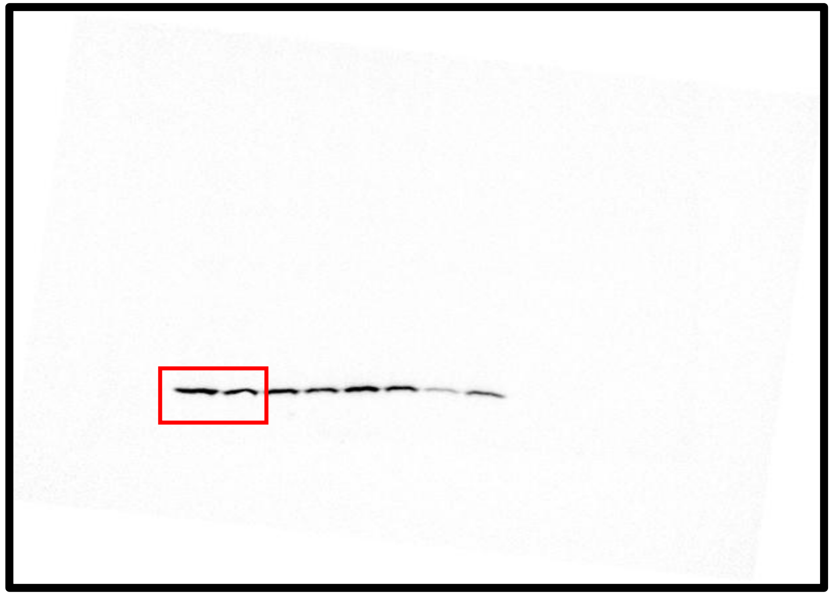

Merge with the marker

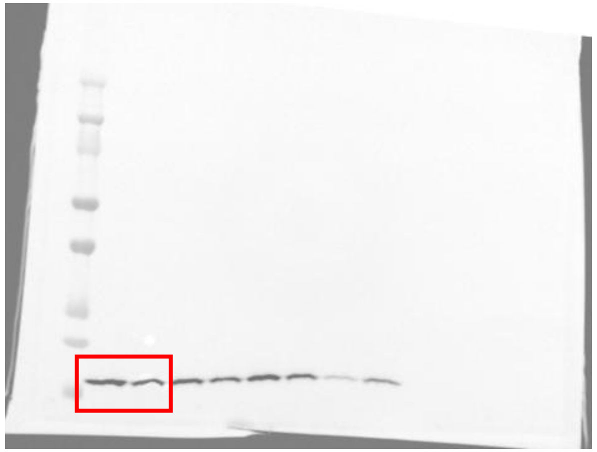

Fig.4M. Parkin

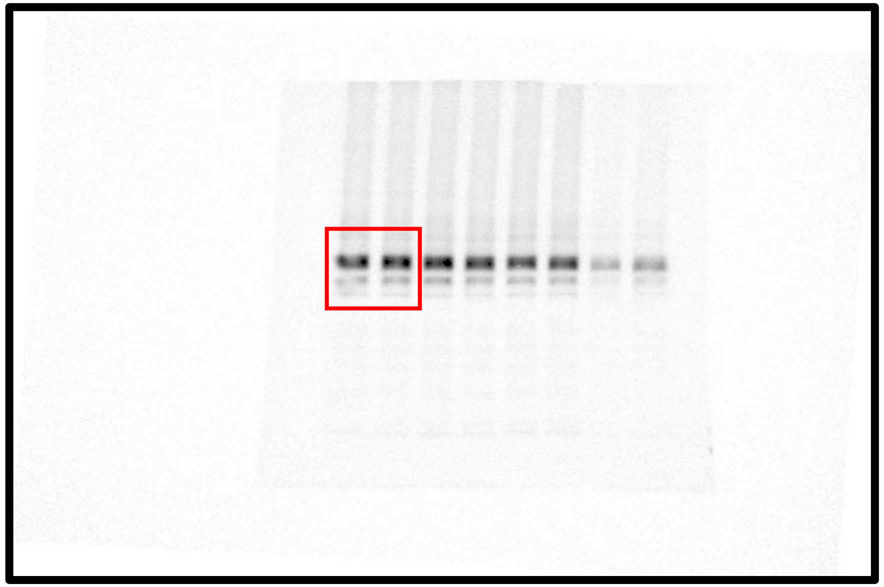

Merge with the marker

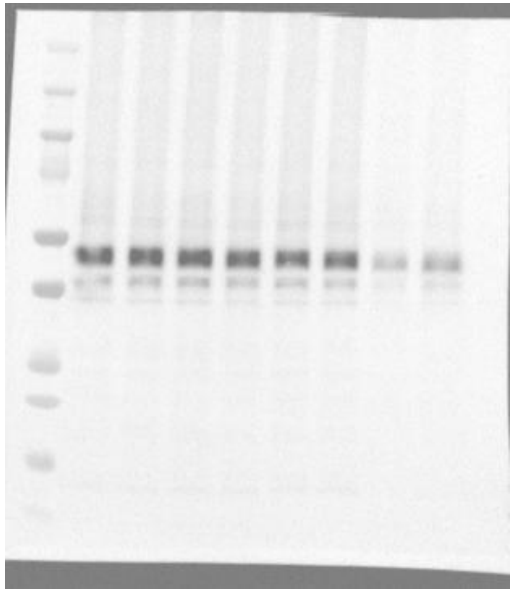

Fig.4M. β-III-tubulin

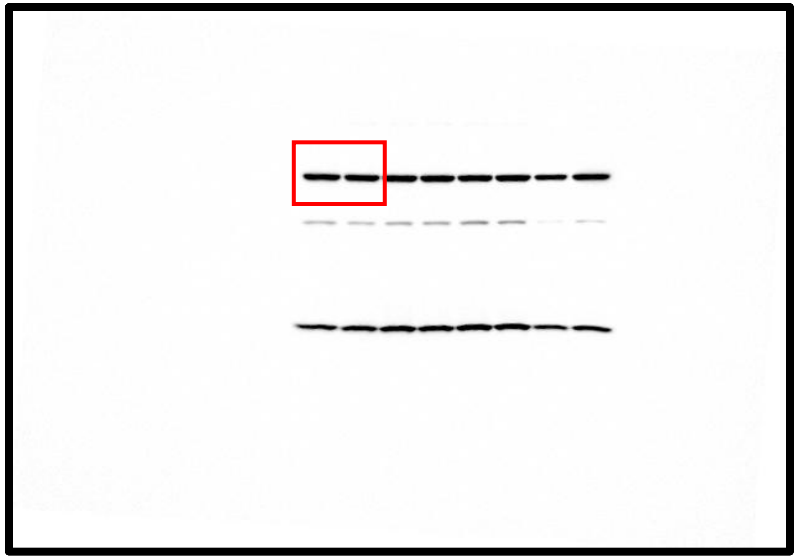

Merge with the marker

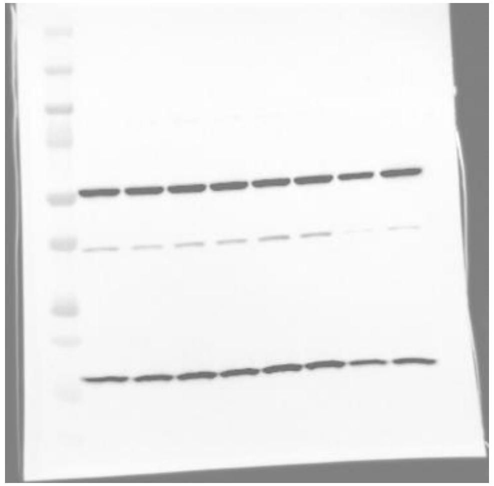

Fig.4M. GAPDH

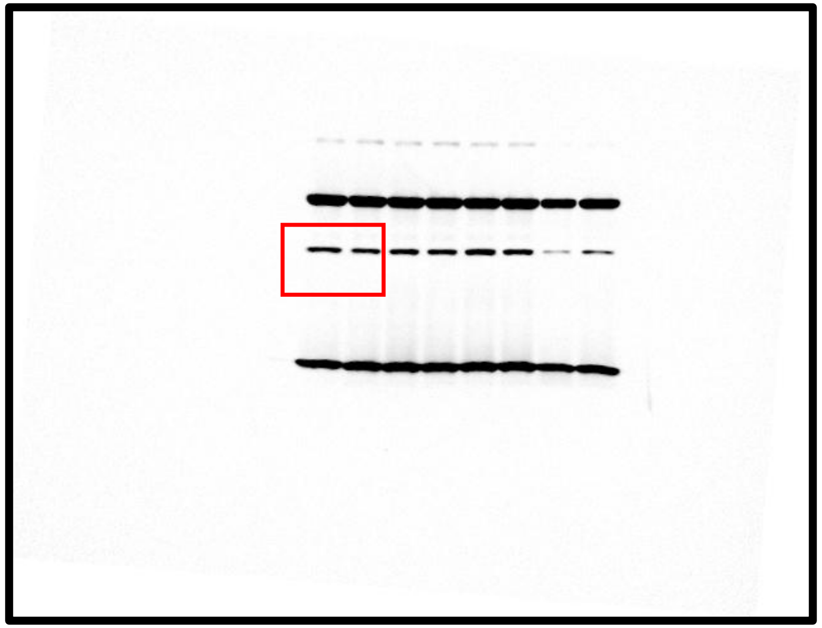

Merge with the marker

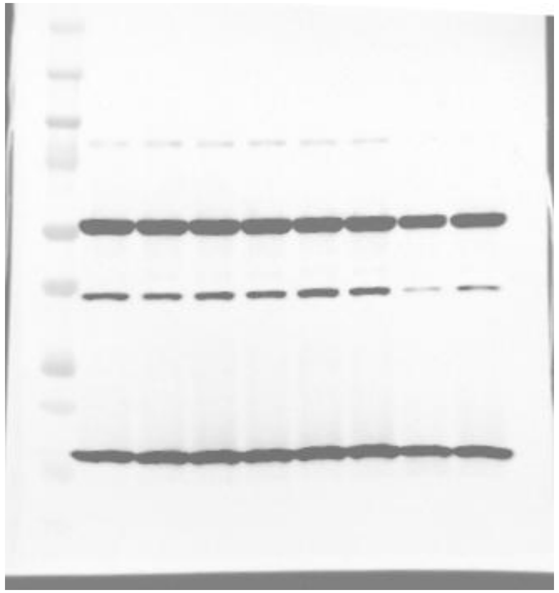

Fig.4O. pS129-syn

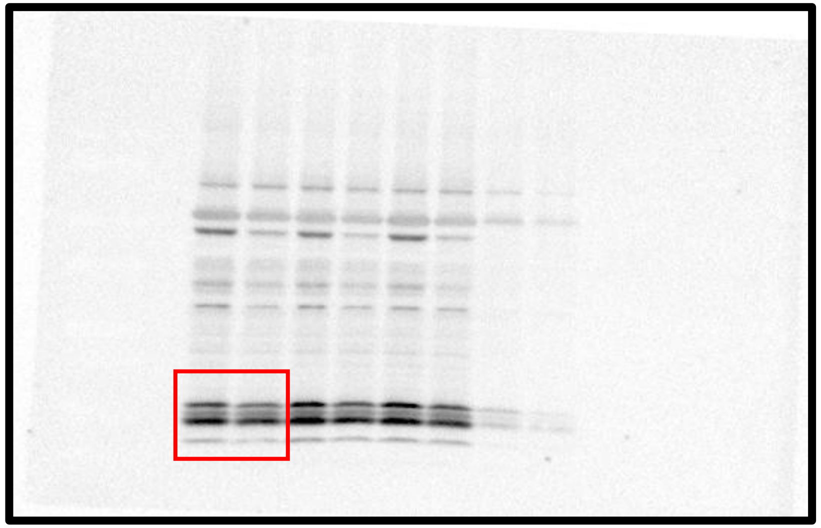

Merge with the marker

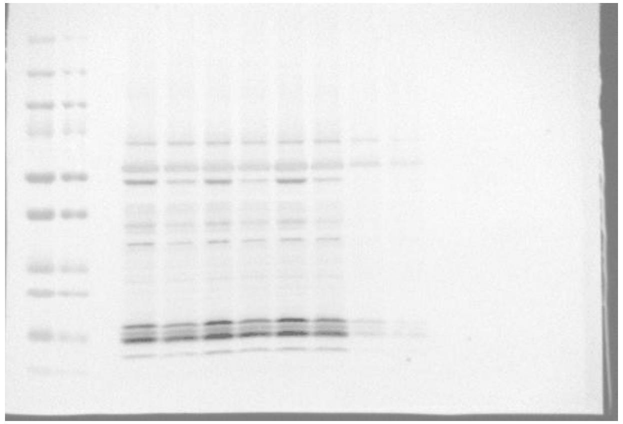

Fig.4O. HTRA1

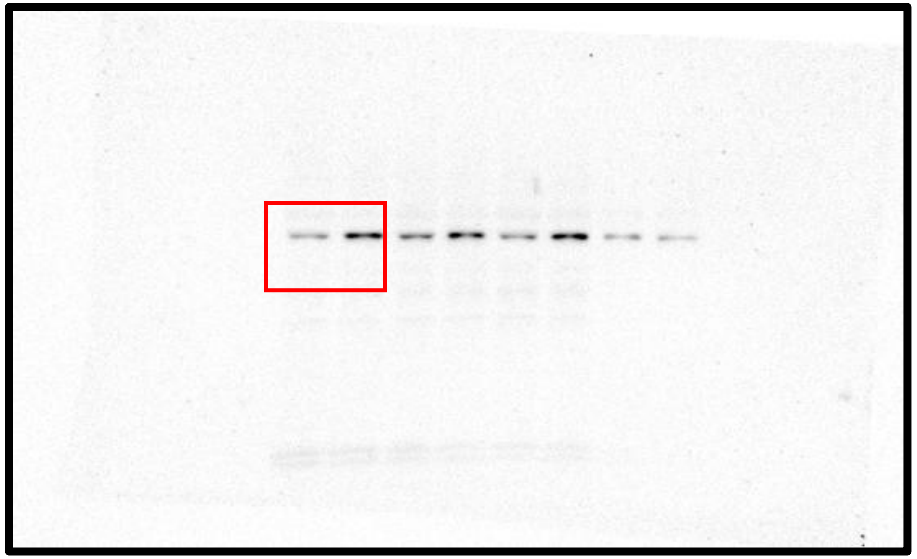

Merge with the marker

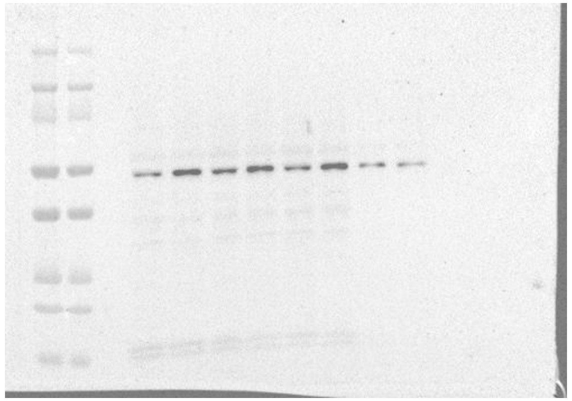

Fig.4O. Gel staining

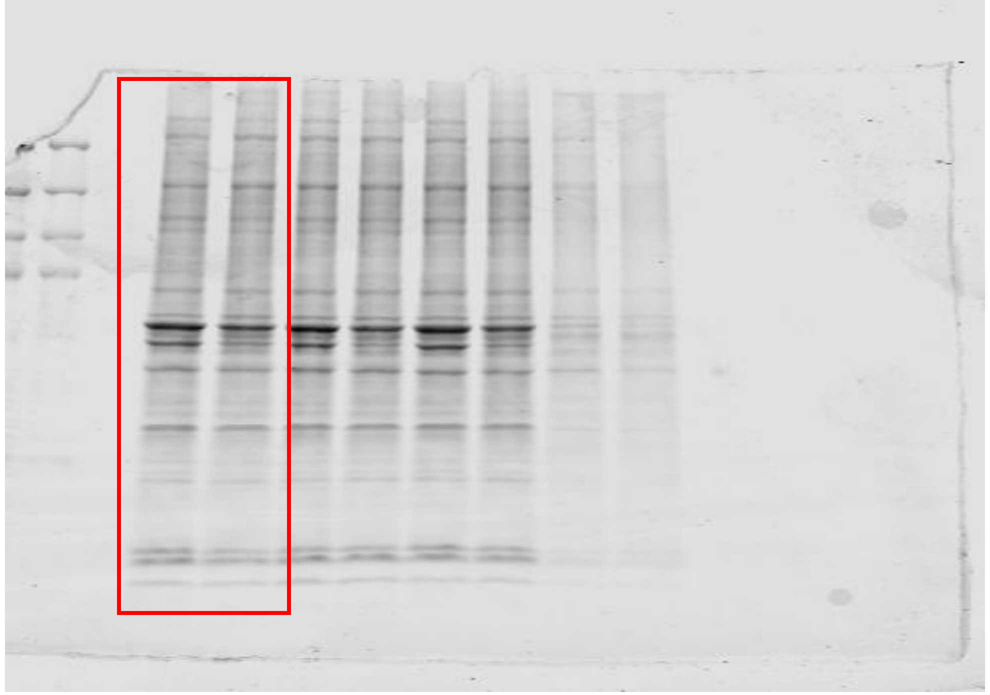

Fig. 5A. pS129-syn

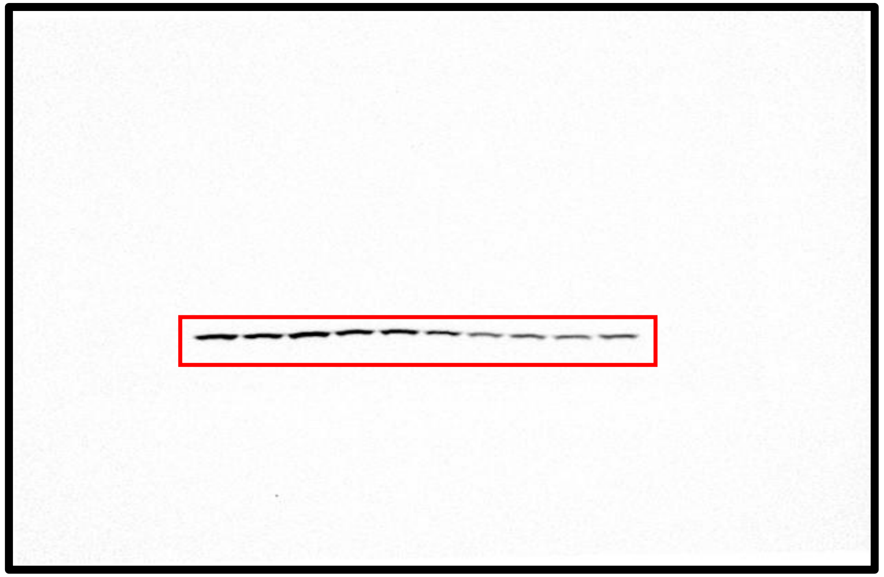

Merge with the marker

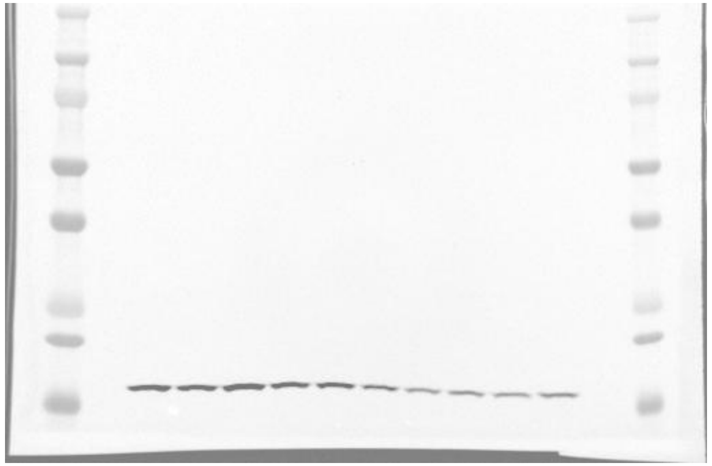

Fig.5A. Parkin

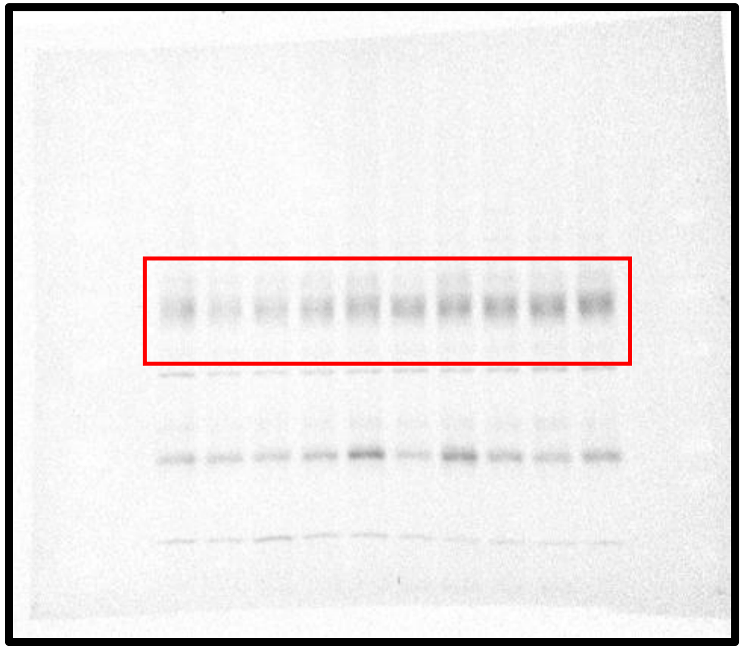

Merge with the marker

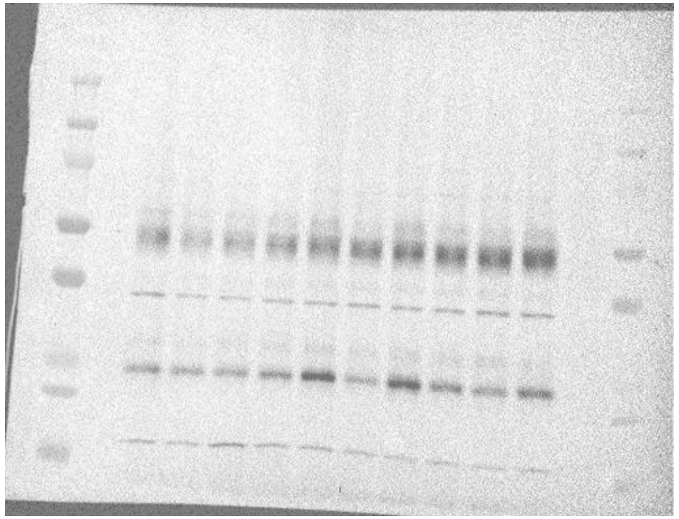

Fig.5A. HTRA1

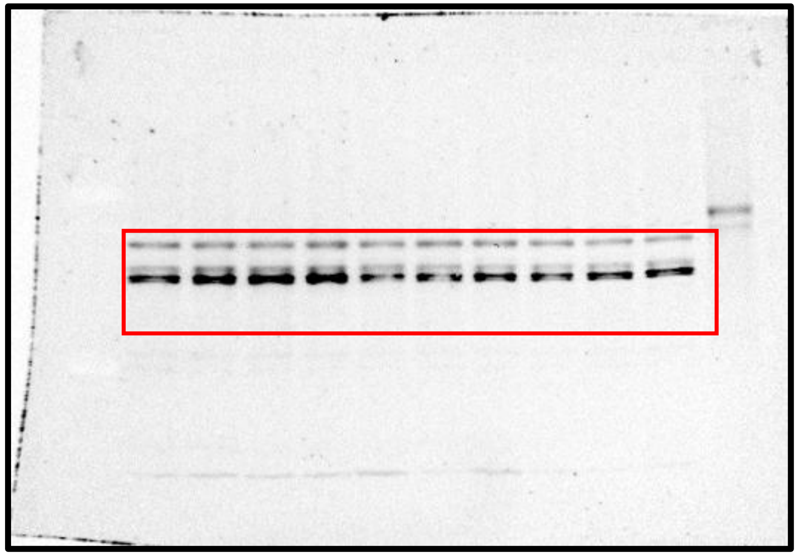

Merge with the marker

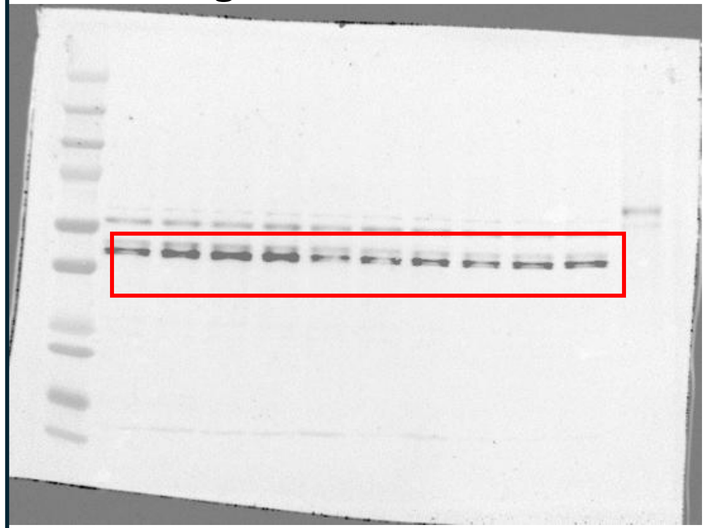

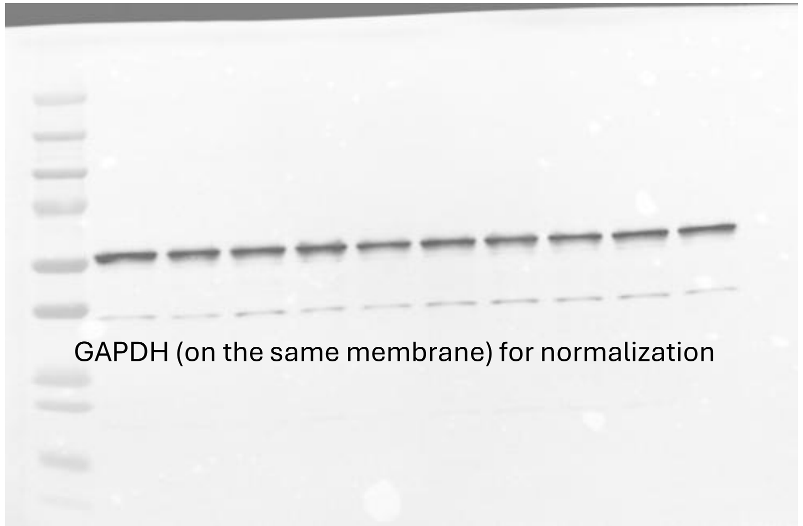

Fig.5A. β-III-tubulin

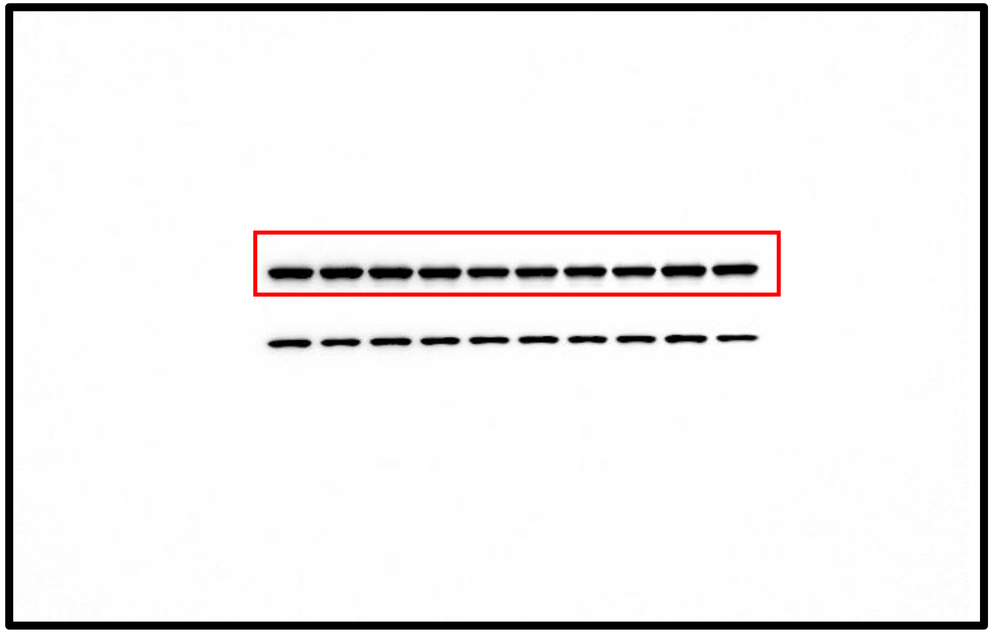

Merge with the marker

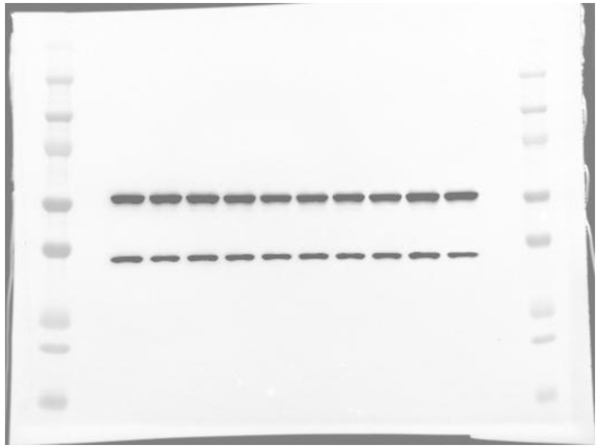

Fig.5A. GAPDH

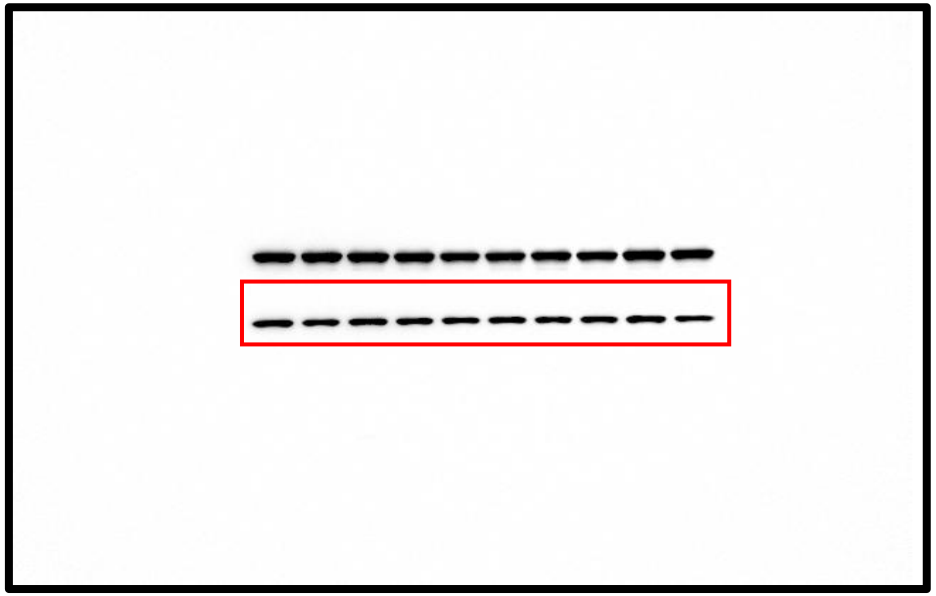

Merge with the marker

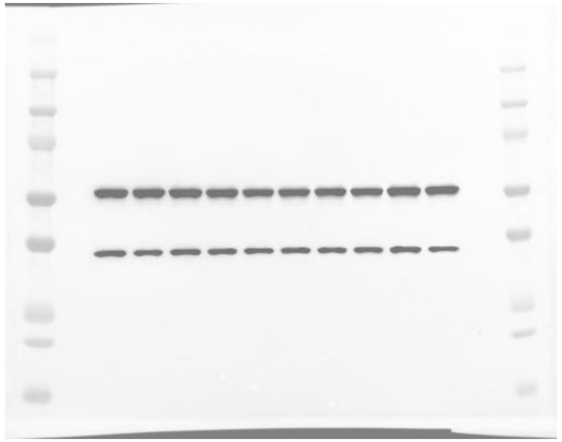

Fig.5C. pS129-syn

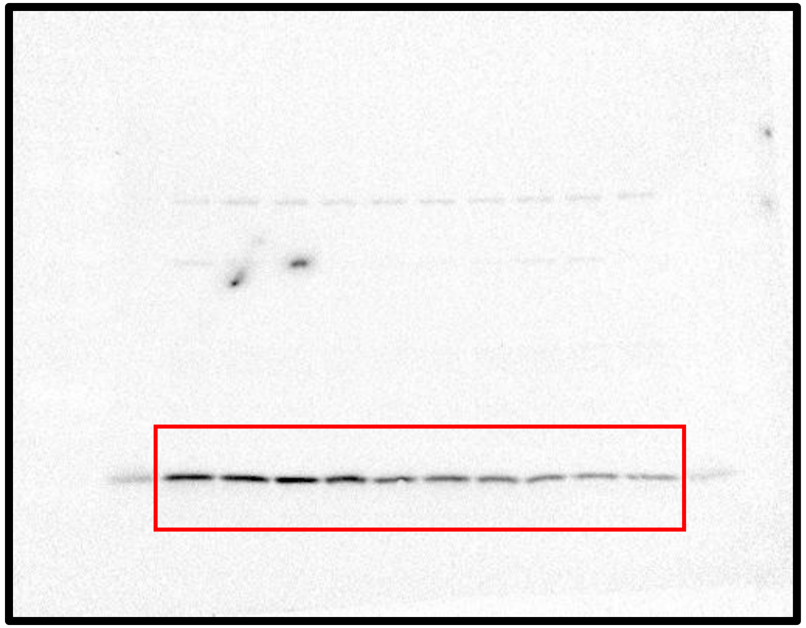

Merge with the marker

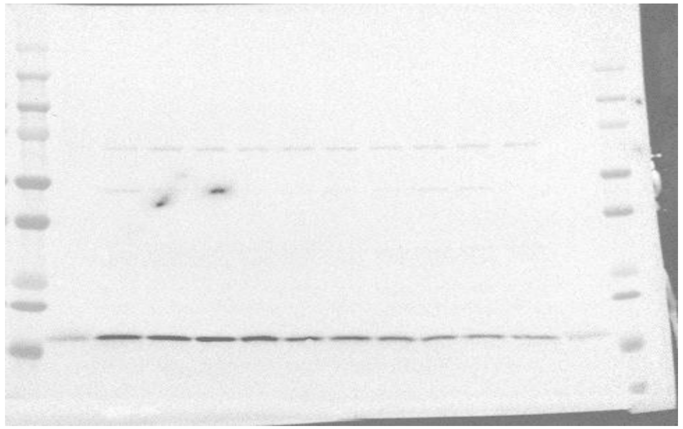

Fig.5C. HTRA1

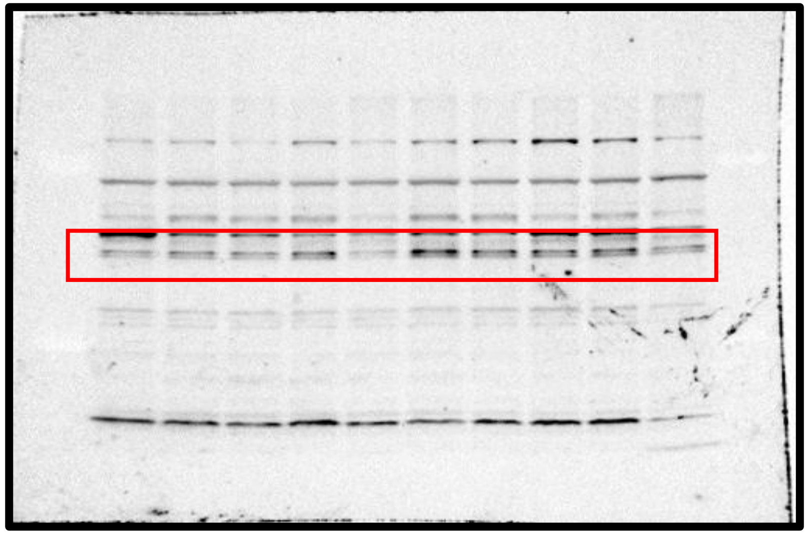

Merge with the marker

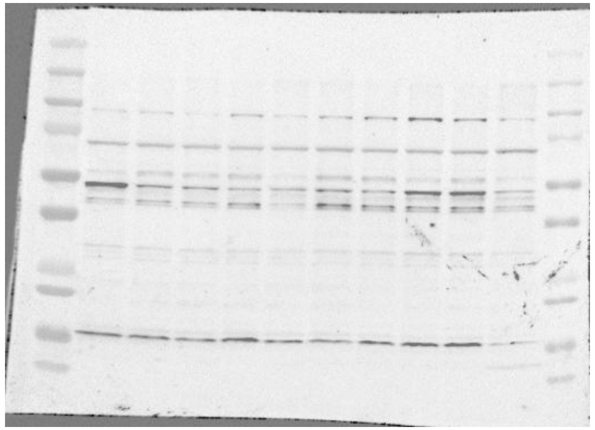

Fig.5C. Gel staining

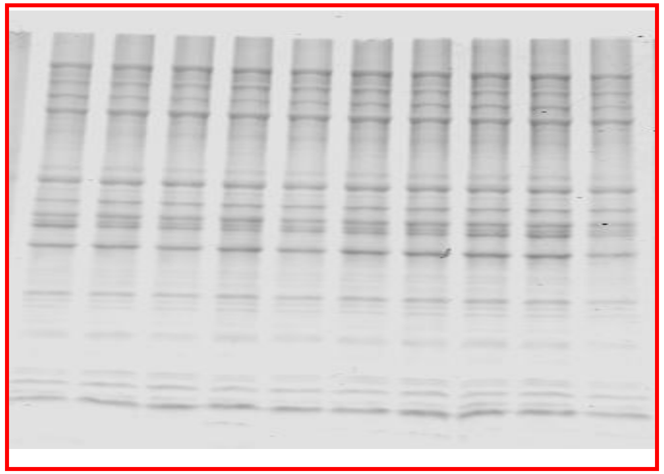

Fig.S1 A. pS129-syn

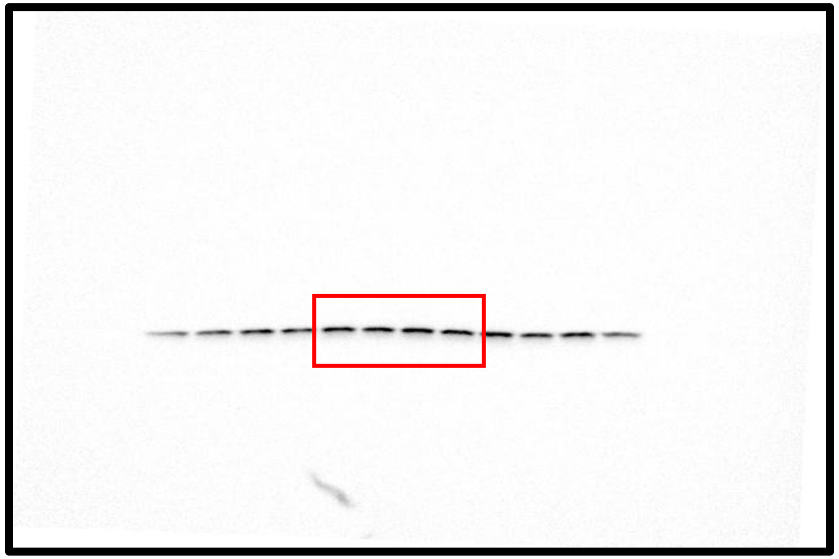

Merge with the marker

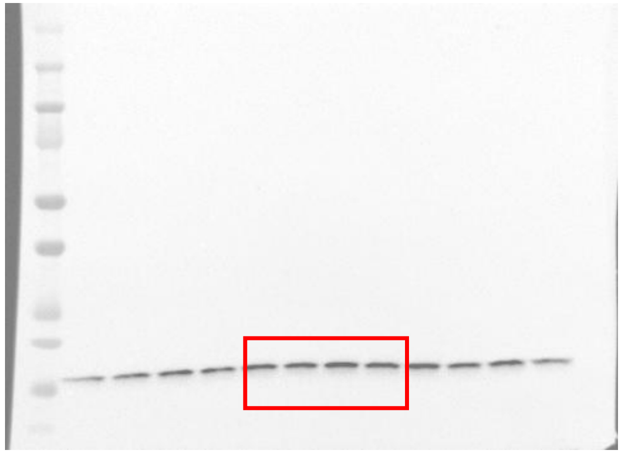

Fig.S1 A. Syn

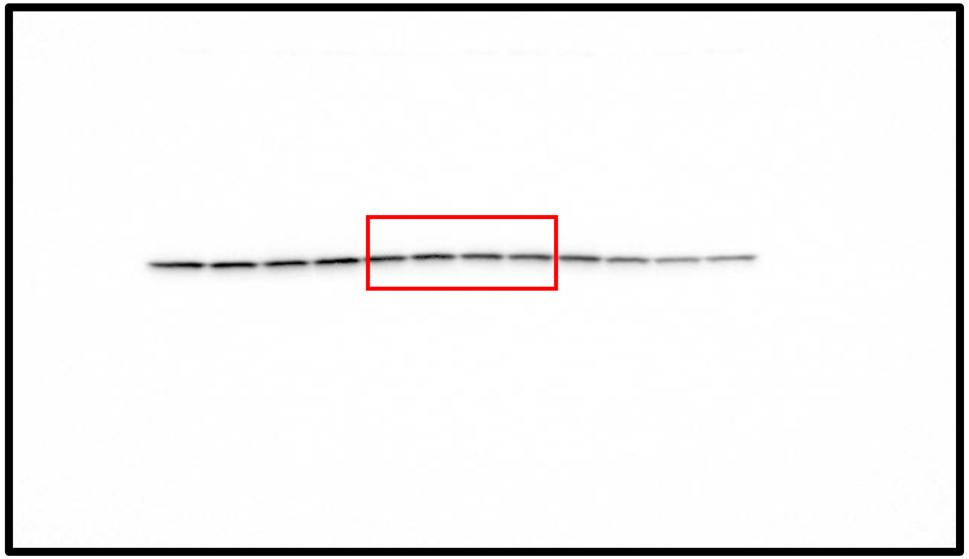

Merge with the marker

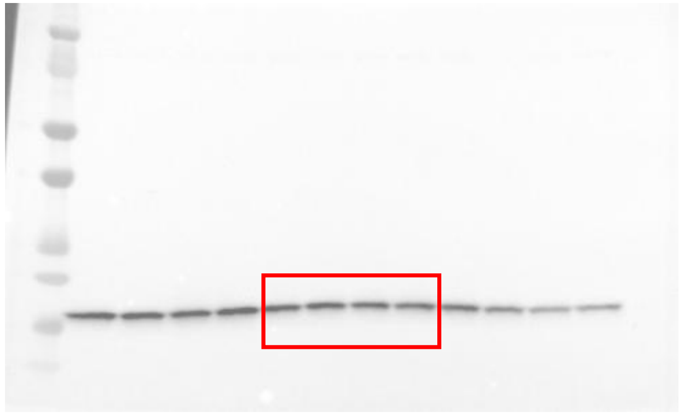

Fig.S1 A. β-III-tubulin

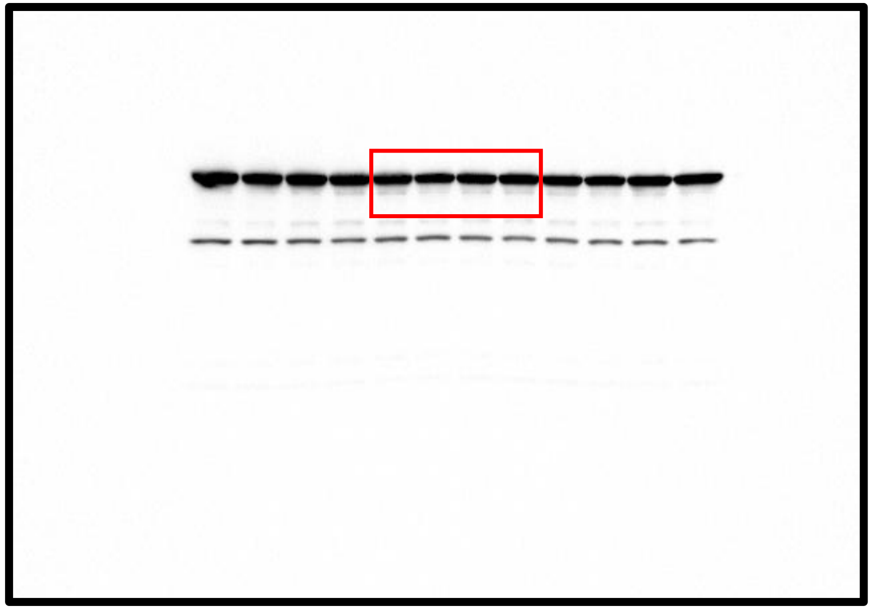

Merge with the marker

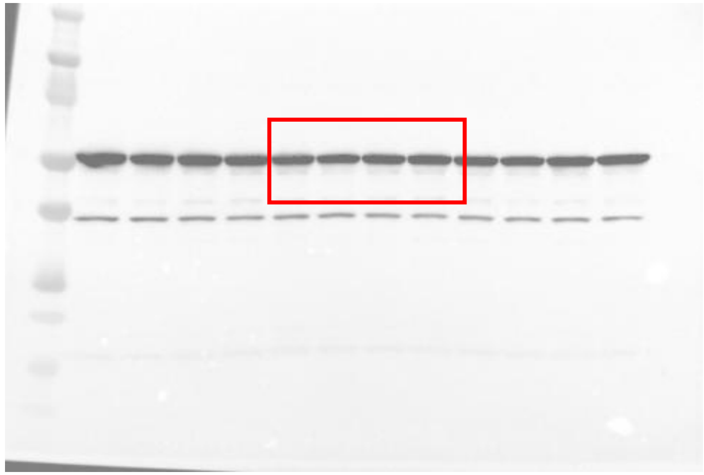

Fig. S1 A. GAPDH

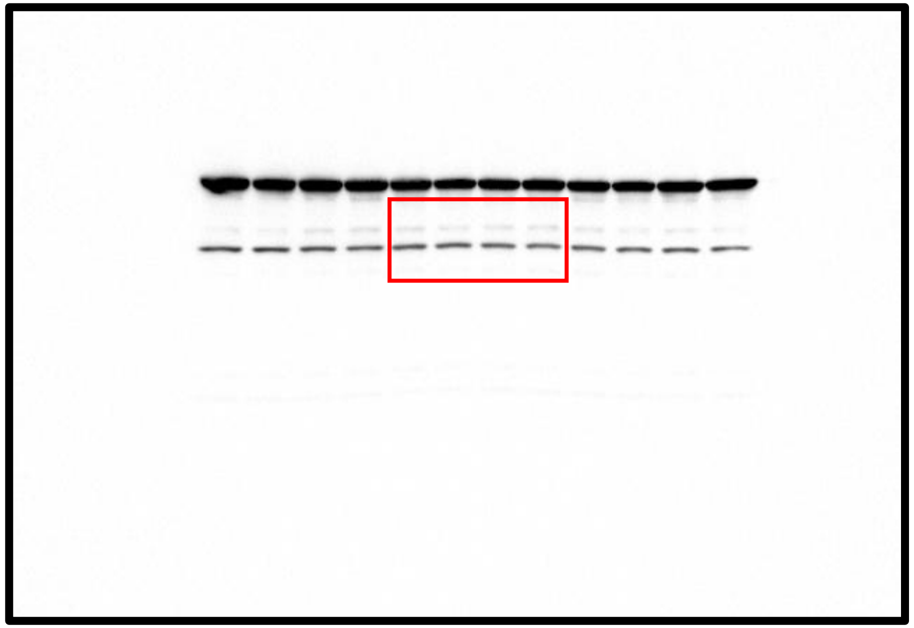

Merge with the marker

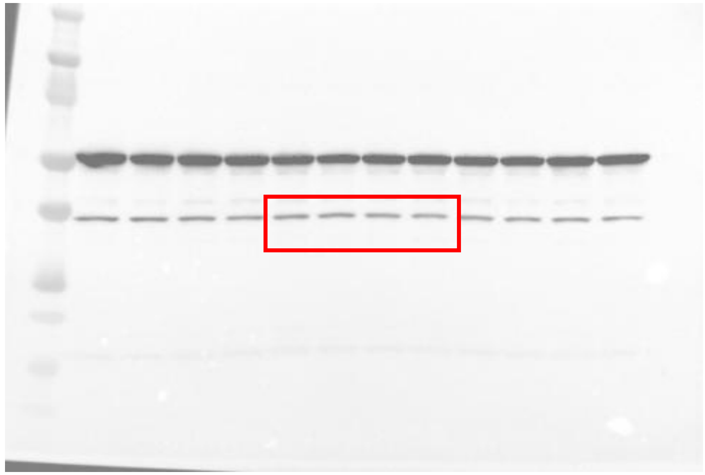

Fig. S1 C. pS129-syn

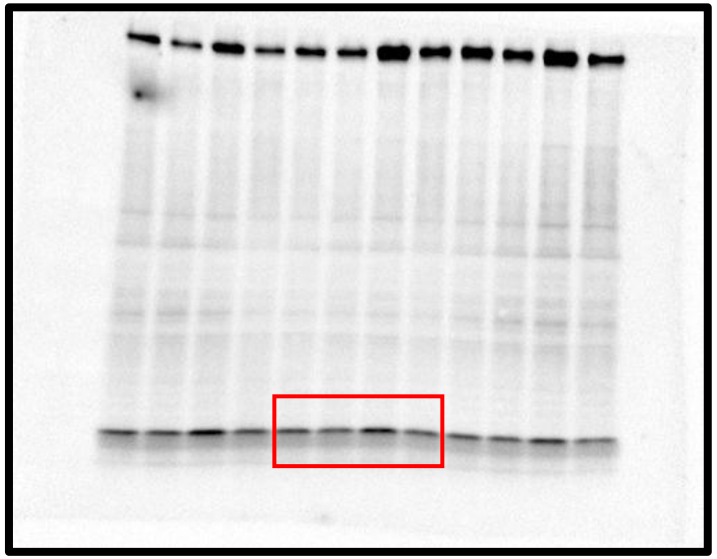

Merge with the marker

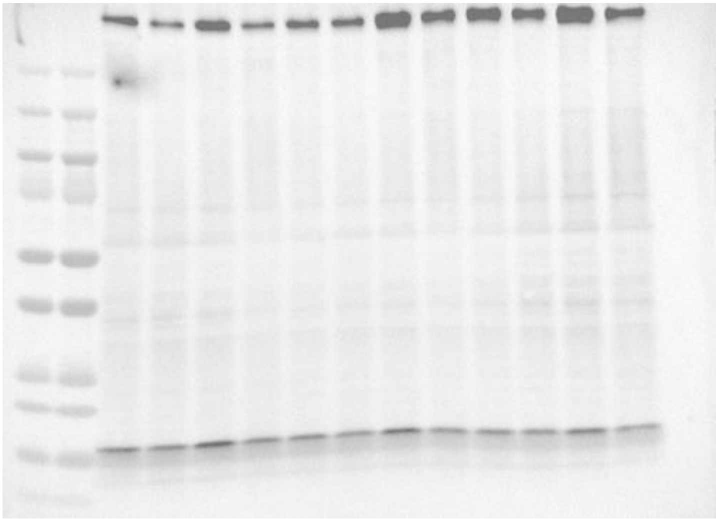

Fig. S1 C. Syn

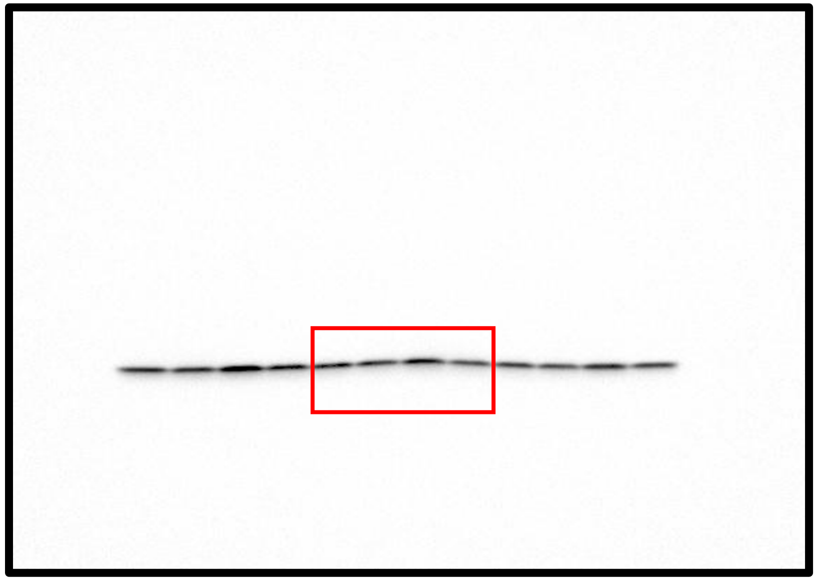

Merge with the marker

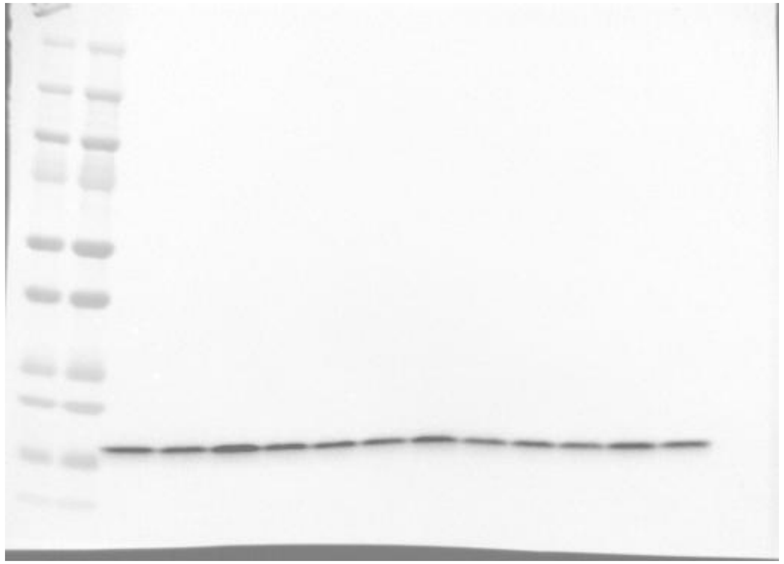

Fig. S1 C. Gel staining

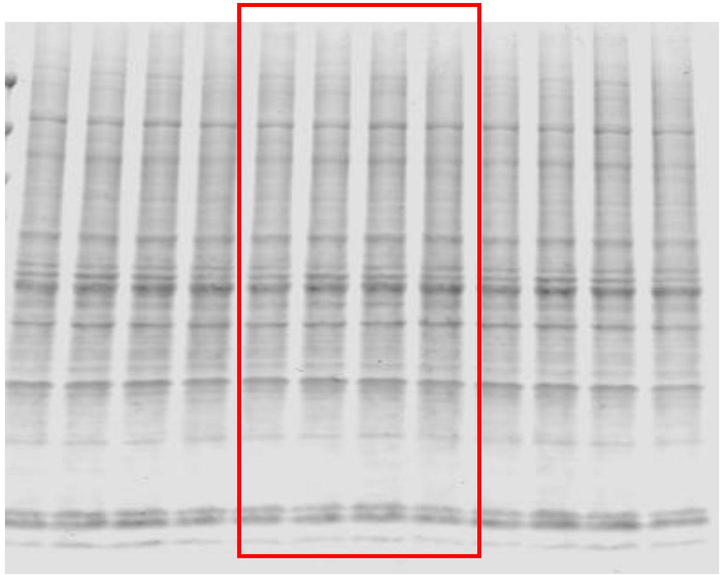

Fig. S3 E. Caspase 3

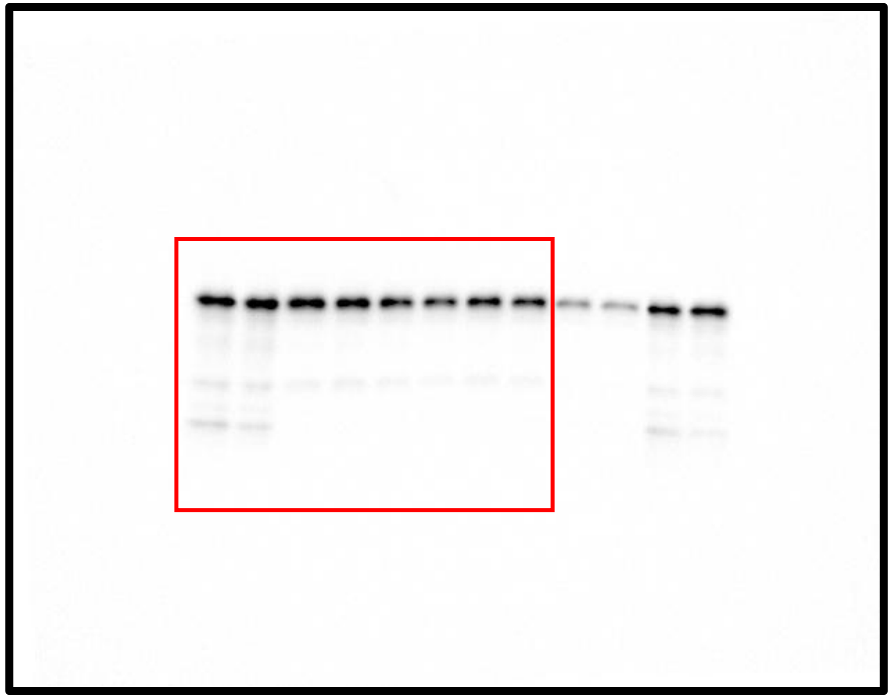

Merge with the marker

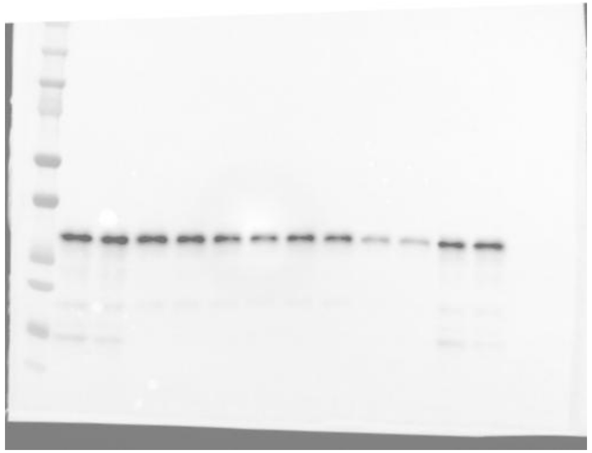

Fig. S3 E. Cleaved Caspase 3

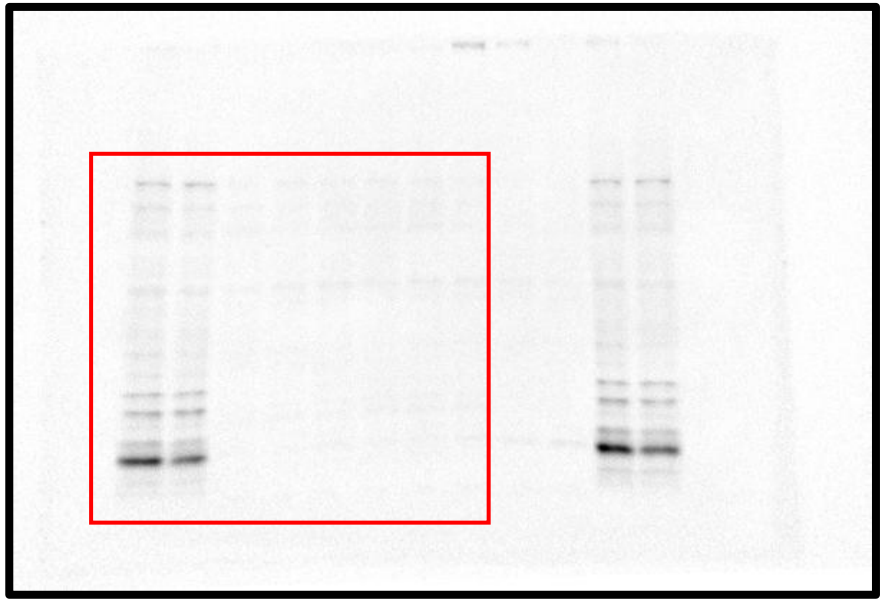

Merge with the marker

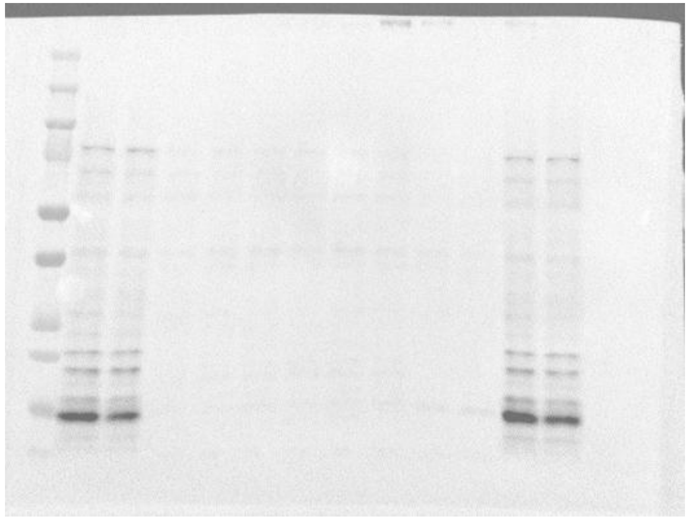

Fig.S3 E. β-III-tubulin

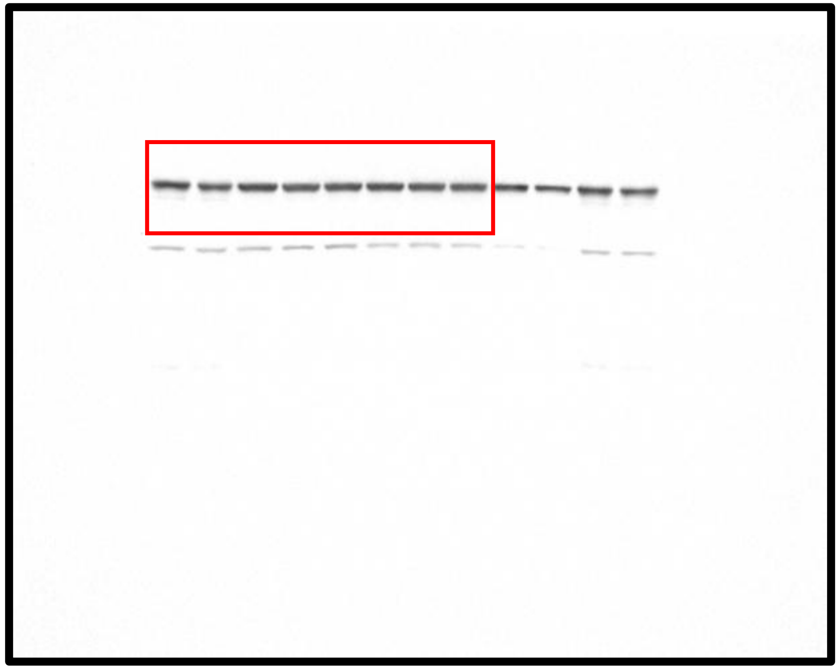

Merge with the marker

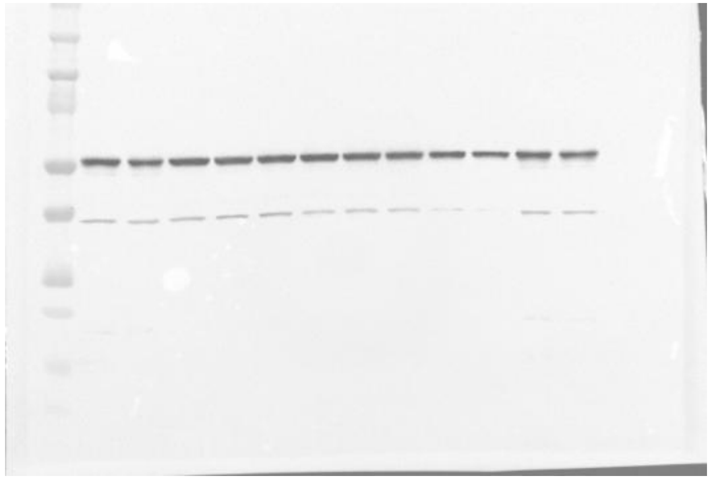

Fig. S3 E. GAPDH

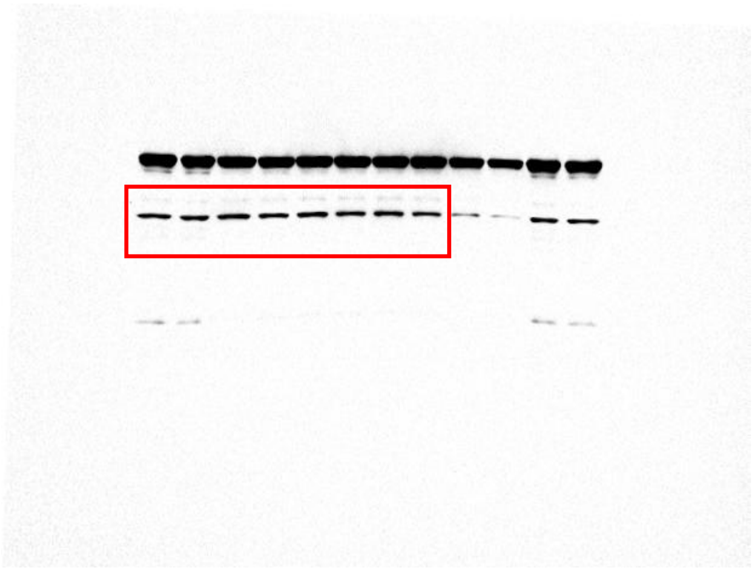

Merge with the marker

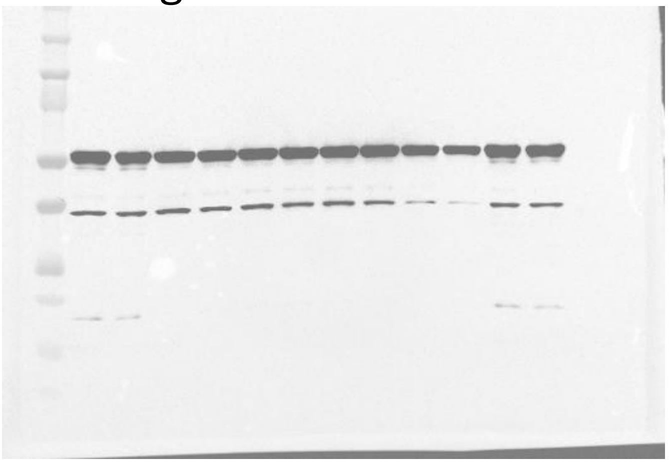

Fig.S5 B. pS129-syn

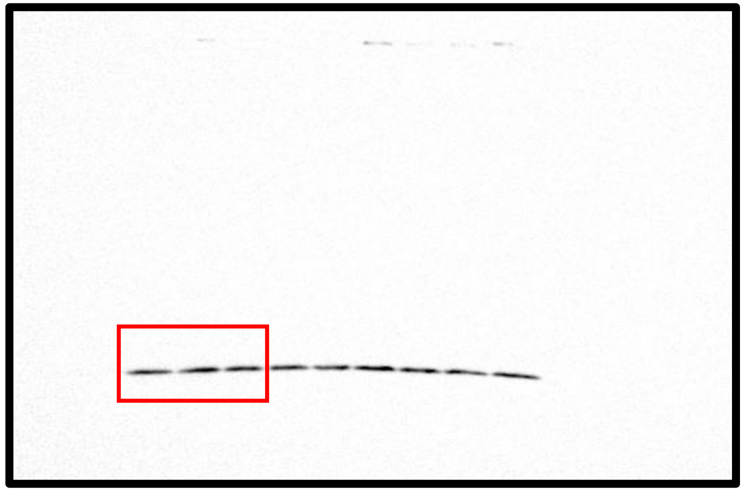

Merge with the marker

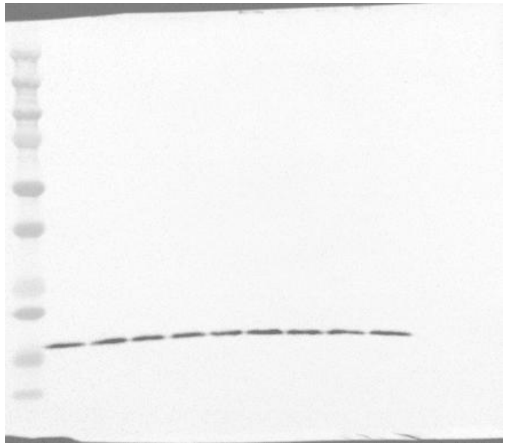

Fig. S5B. GAPDH

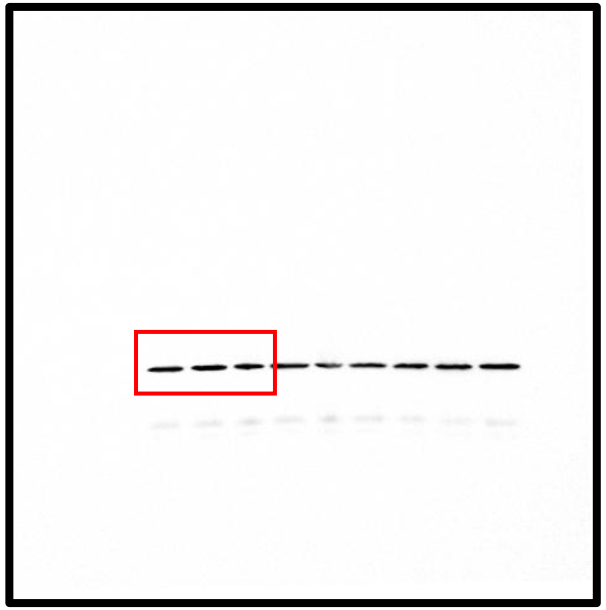

Merge with the marker

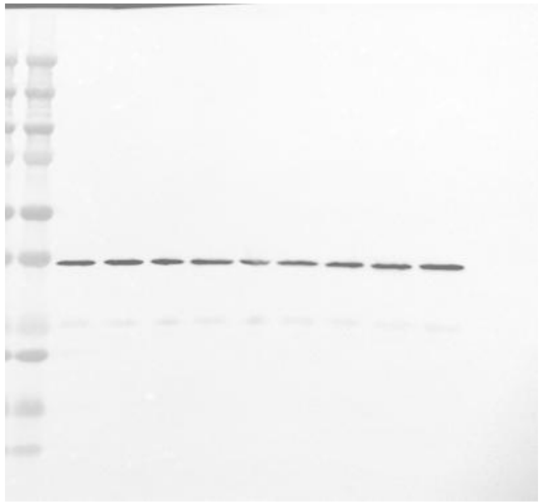

Fig.S5 D. HTRA1

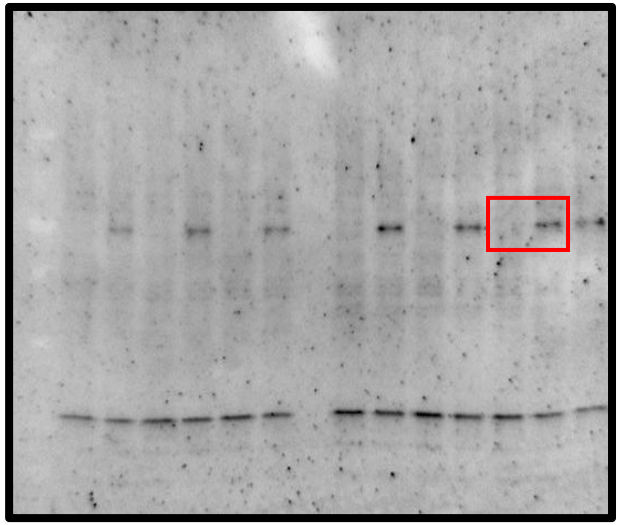

Merge with the marker

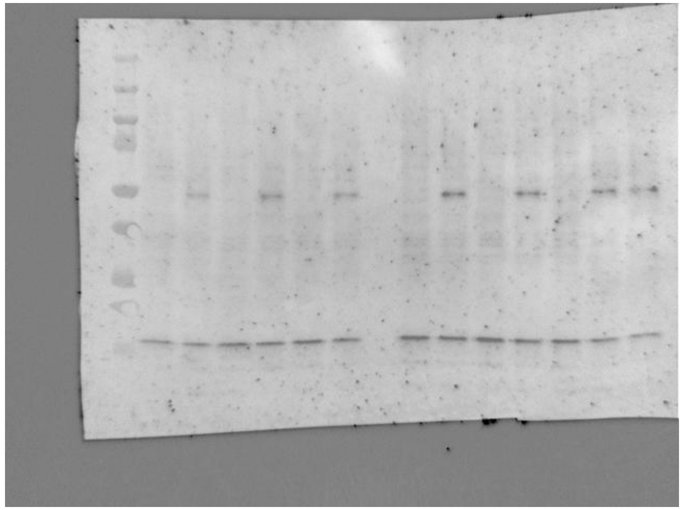

Fig. S5D. pS129-syn

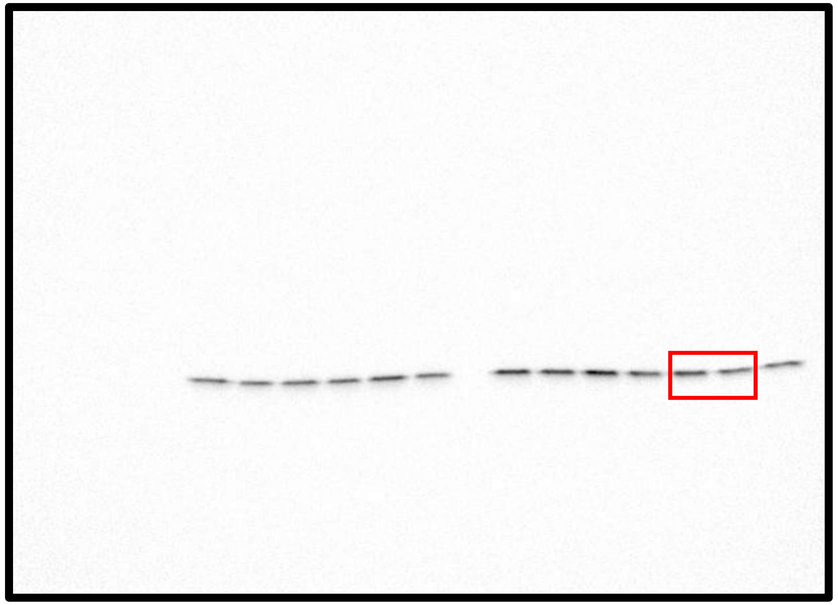

Merge with the marker

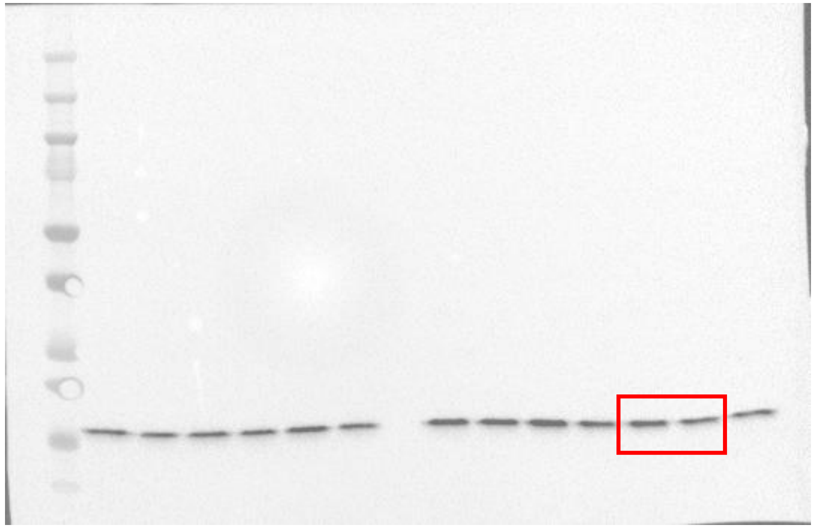

Fig. S5 D. GAPDH

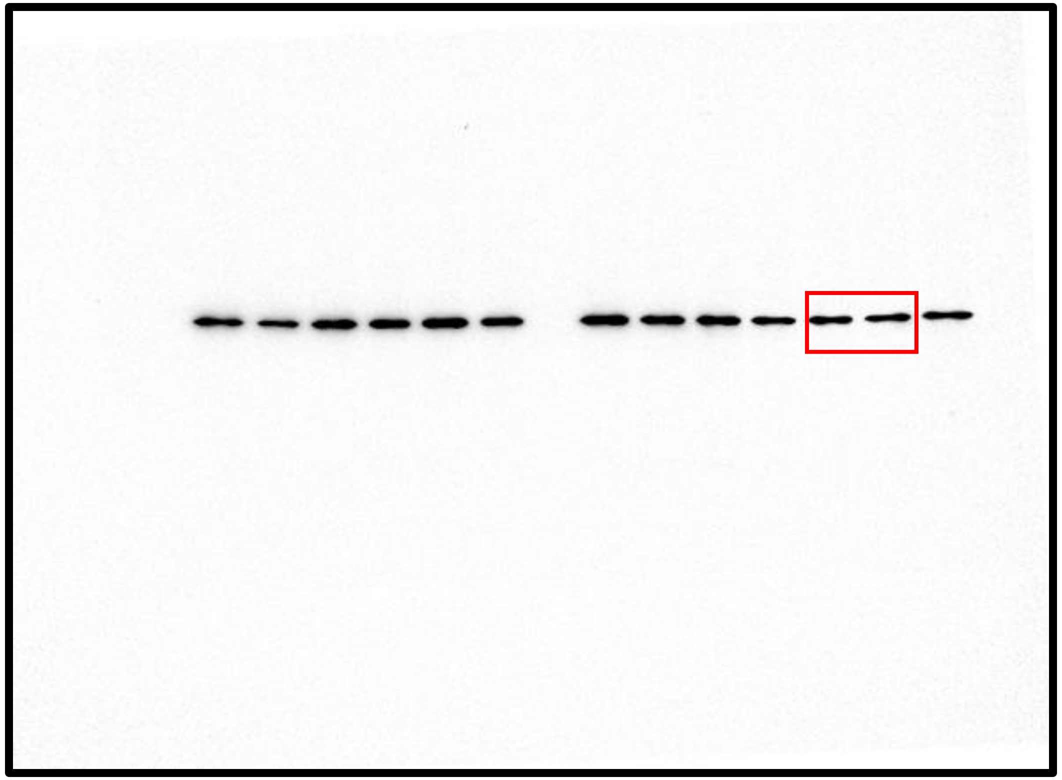

Merge with the marker

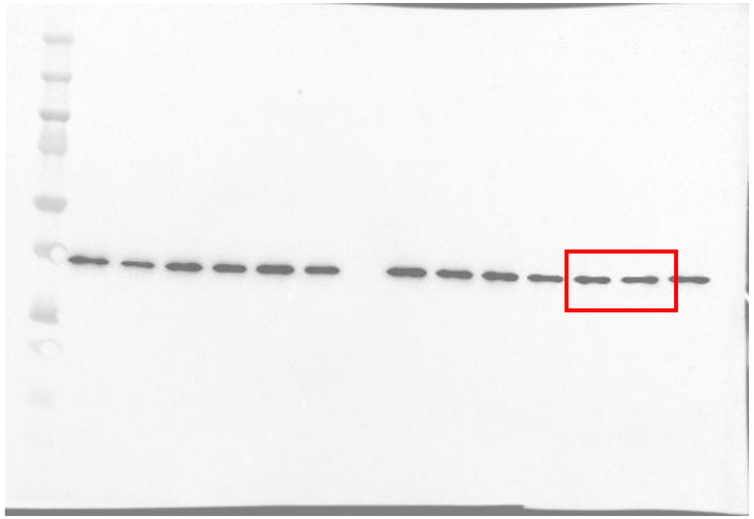

Fig.S6 B. DAT

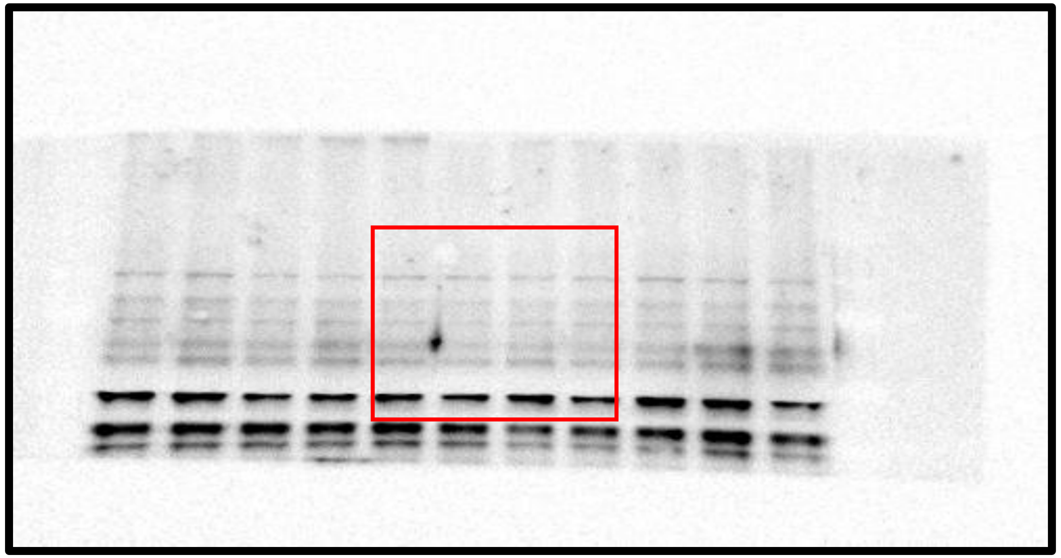

Merge with the marker

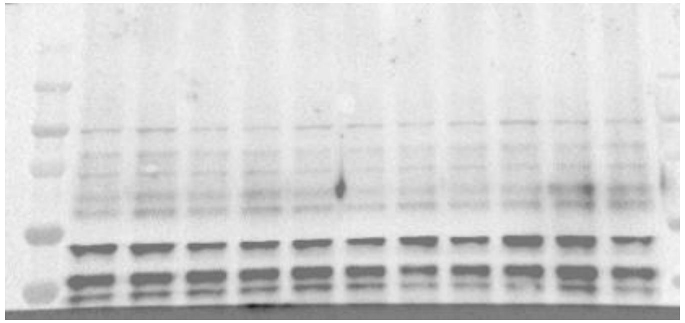

The membrane had been cut below 50kD before imaging

Fig.S6B. β-III-tubulin

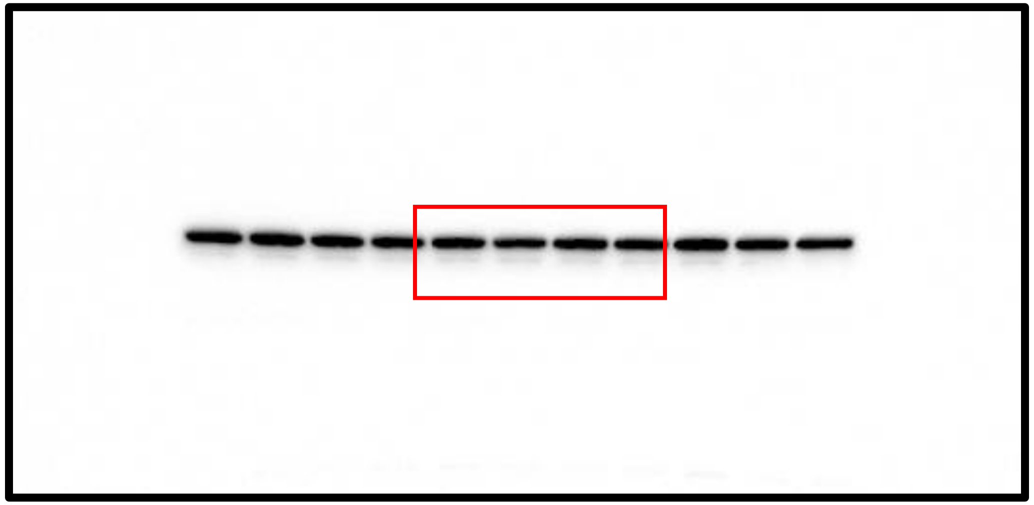

Merge with the marker

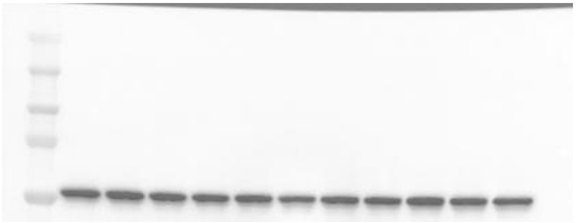

The membrane had been cut below 50kD before imaging

Fig.S8 A. pS129-syn

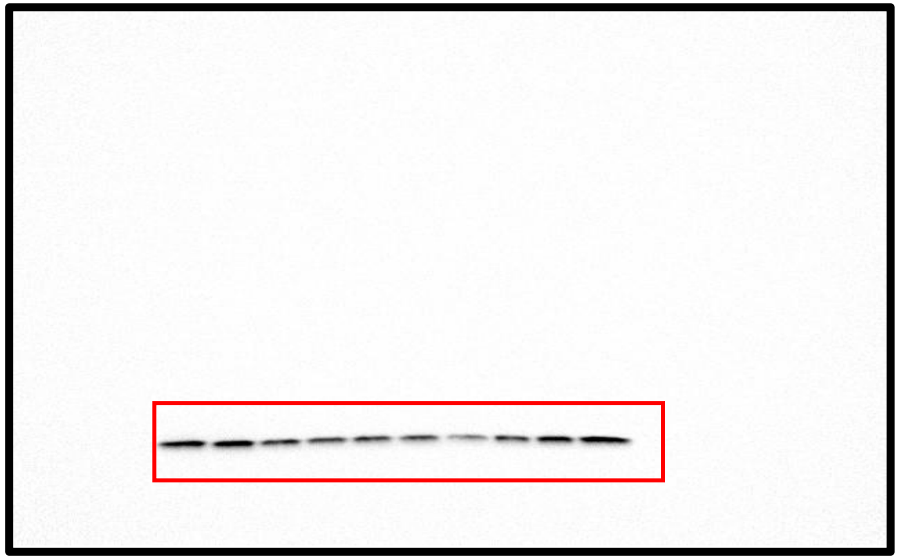

Merge with the marker

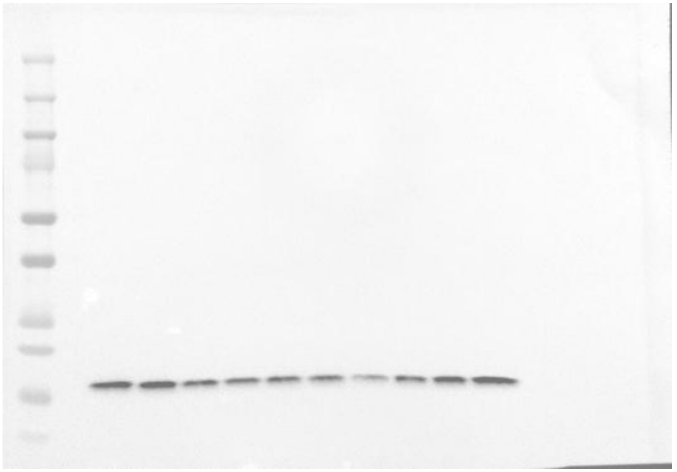

Fig.S8 A. β-III-tubulin

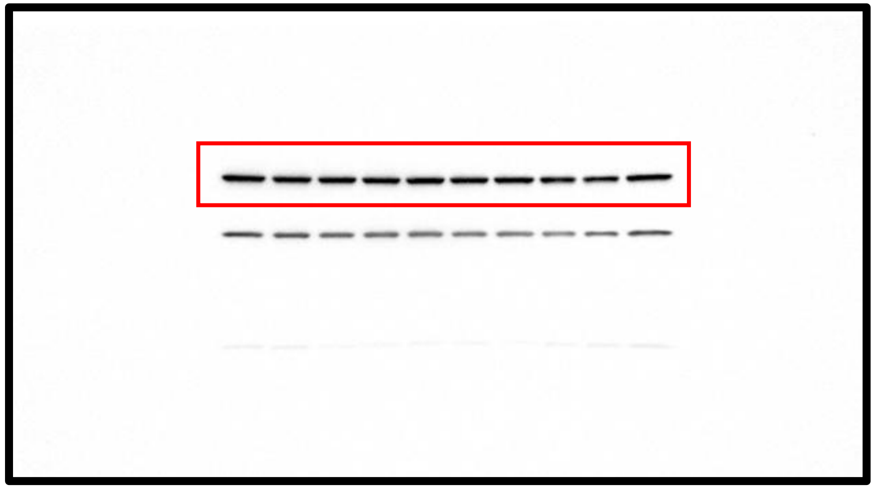

Merge with the marker

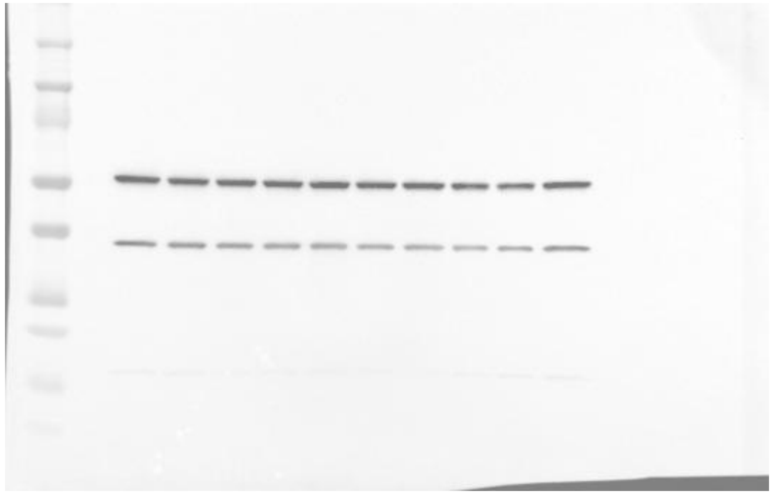

Fig. S8A. GAPDH

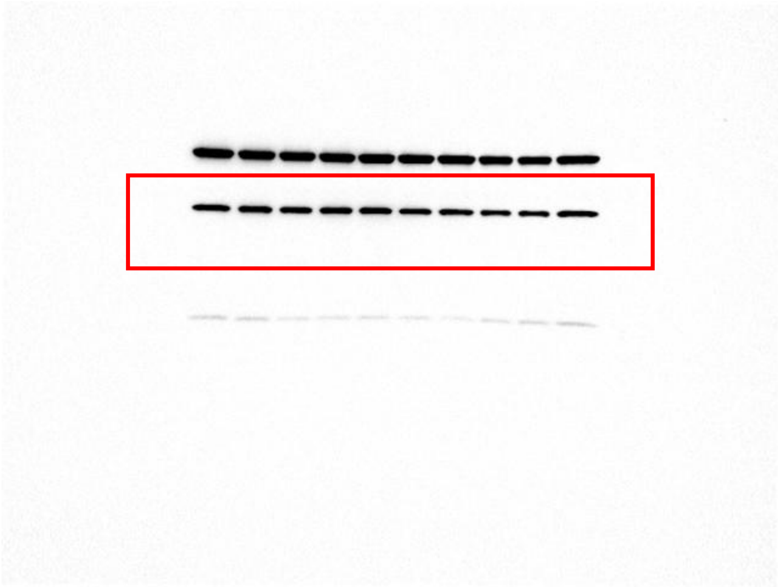

Merge with the marker

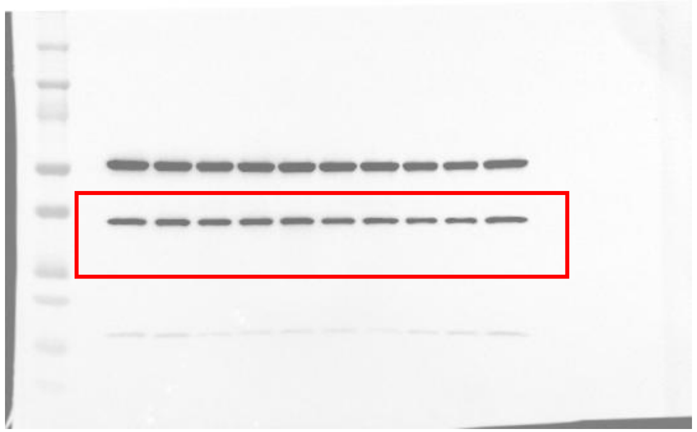

Fig. S8 C. pS129-syn

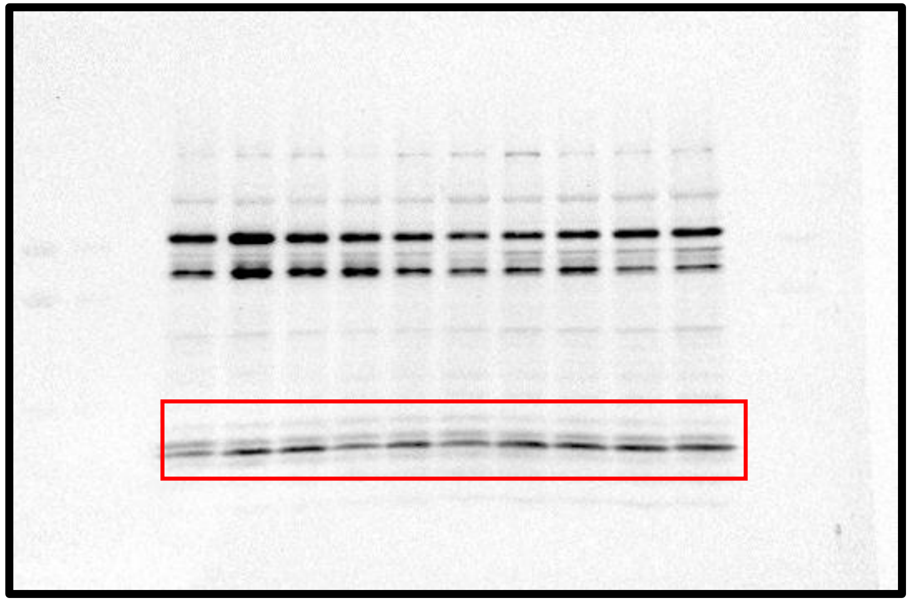

Merge with the marker

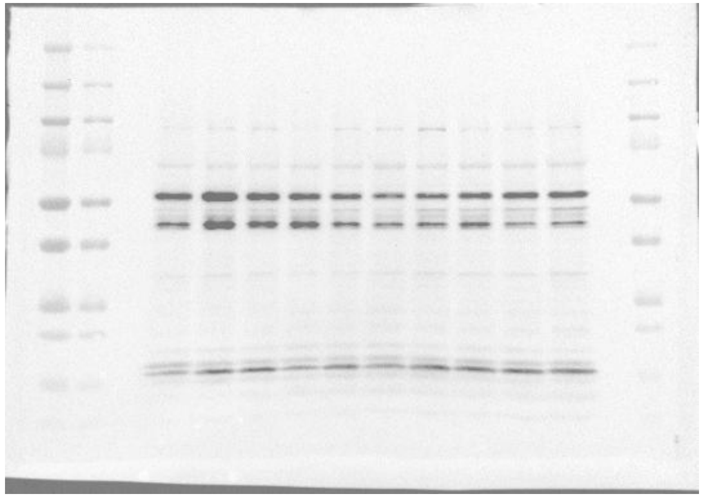

Fig. S8 C. Gel staining

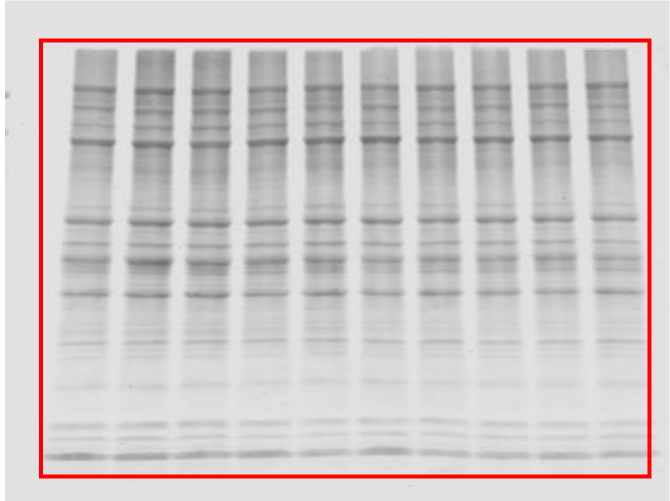

Fig. S8 E. HTRA1

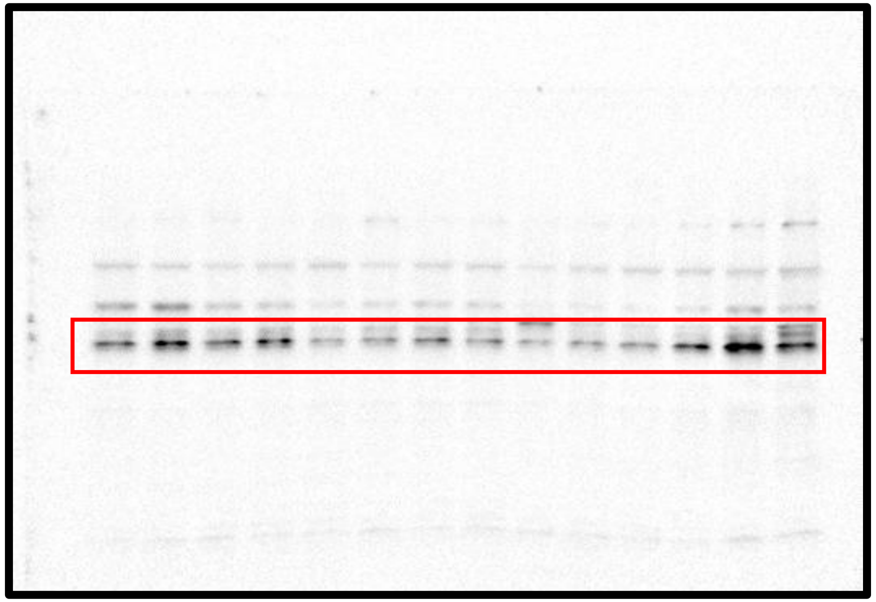

Merge with the marker

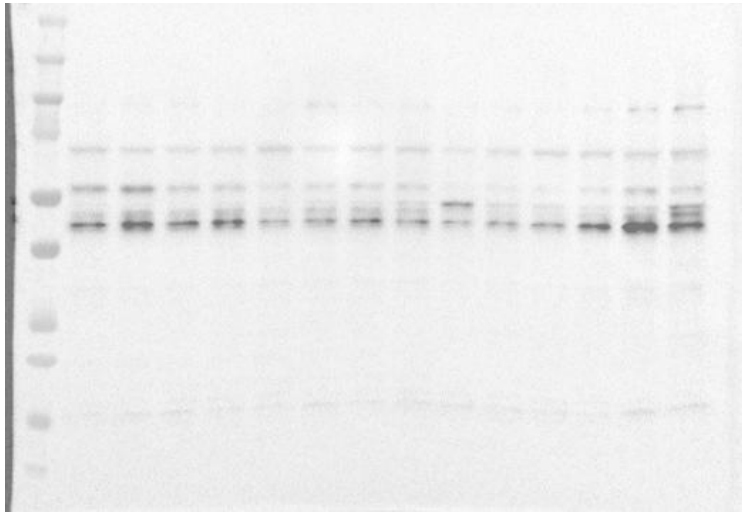

Fig. S8 E. Gel staining

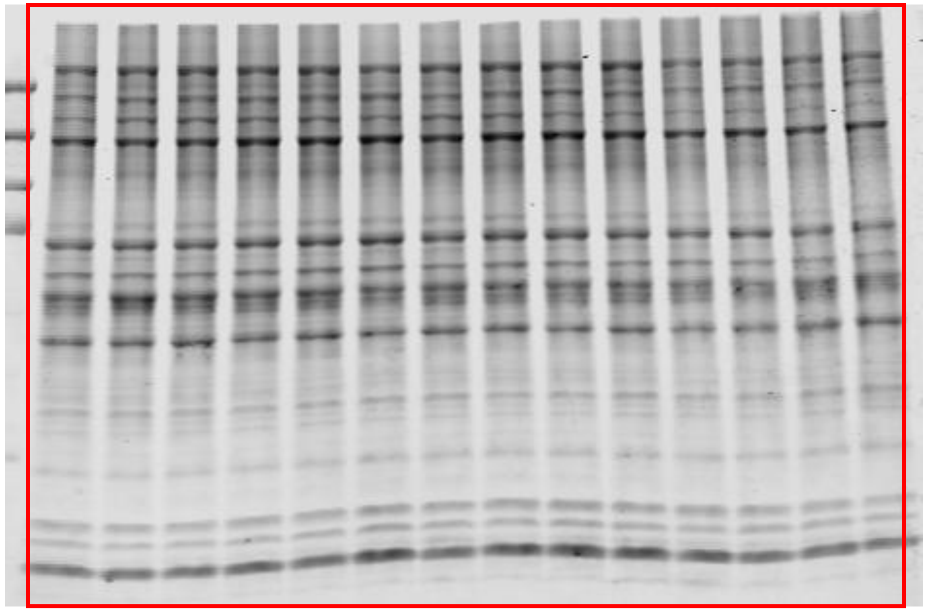

Supplement: Unedited blot and gel images [file jci-136-196137-s171.pdf]
